# Gene ARMADA: an integrated multi-analysis platform for microarray data implemented in MATLAB

**DOI:** 10.1186/1471-2105-10-354

**Published:** 2009-10-27

**Authors:** Aristotelis Chatziioannou, Panagiotis Moulos, Fragiskos N Kolisis

**Affiliations:** 1 Metabolic Engineering and Bioinformatics Group, Institute of Biological Research and Biotechnology, National Hellenic Research Foundation, 48 Vassileos Constantinou ave., 11635, Athens, Greece

## Abstract

**Background:**

The microarray data analysis realm is ever growing through the development of various tools, open source and commercial. However there is absence of predefined rational algorithmic analysis workflows or batch standardized processing to incorporate all steps, from raw data import up to the derivation of significantly differentially expressed gene lists. This absence obfuscates the analytical procedure and obstructs the massive comparative processing of genomic microarray datasets. Moreover, the solutions provided, heavily depend on the programming skills of the user, whereas in the case of GUI embedded solutions, they do not provide direct support of various raw image analysis formats or a versatile and simultaneously flexible combination of signal processing methods.

**Results:**

We describe here Gene ARMADA (Automated Robust MicroArray Data Analysis), a MATLAB implemented platform with a Graphical User Interface. This suite integrates all steps of microarray data analysis including automated data import, noise correction and filtering, normalization, statistical selection of differentially expressed genes, clustering, classification and annotation. In its current version, Gene ARMADA fully supports 2 coloured cDNA and Affymetrix oligonucleotide arrays, plus custom arrays for which experimental details are given in tabular form (Excel spreadsheet, comma separated values, tab-delimited text formats). It also supports the analysis of already processed results through its versatile import editor. Besides being fully automated, Gene ARMADA incorporates numerous functionalities of the Statistics and Bioinformatics Toolboxes of MATLAB. In addition, it provides numerous visualization and exploration tools plus customizable export data formats for seamless integration by other analysis tools or MATLAB, for further processing. Gene ARMADA requires MATLAB 7.4 (R2007a) or higher and is also distributed as a stand-alone application with MATLAB Component Runtime.

**Conclusion:**

Gene ARMADA provides a highly adaptable, integrative, yet flexible tool which can be used for automated quality control, analysis, annotation and visualization of microarray data, constituting a starting point for further data interpretation and integration with numerous other tools.

## Background

Functional genomics represent a hot topic in biological research nowadays, embracing the analysis of large datasets, through the quantitative measurement of the genomic expression of the organisms probed, under numerous conditions. Gene expression microarrays represent an established, high-throughput measurement technology. It is an indispensable tool for genome-wide inspection of changes in the total gene expression of an organism, which proved to be a major discovery tool in biological research. Important goals of global gene expression monitoring experiments are, among others, the identification of significant alterations in transcript levels resulting from the exposure of a living system to a given agent at a specific administration regime [1] or the derivation of prognostic and diagnostic genetic signatures. Additionally, high-throughput gene expression profiling is used in clinical studies in order to: i) identify and categorize diagnostic or prognostic biomarkers ii) classify diseases, e.g. tumours with different prognosis that are indistinguishable by microscopic examination iii) monitor the response to therapy and iv) understand the mechanisms involved in the genesis of disease processes [2]. However, the vast amount of data produced from even a single microarray slide, requires powerful computational tools in order to mine valuable information. With statistical scaling and proper normalization, Differentially Expressed (DE) genes can be selected on a statistical basis and provide investigators with a sound basis for meta-analysis and further research focused on specific biological processes or pathways.

In a typical microarray gene profiling experiment, mRNA is extracted from cells or tissues of interest, reverse-transcribed, labeled and hybridized onto the array surface. Subsequently, washing under stringent protocols diminishes through dilution the possibility of non-specific hybridization. The next step is image acquisition and segmentation. Thus, quantitative estimates of the relative fluorescence intensities of each spot/probeset, related to a gene, are obtained. The signal values thus acquired form a dataset, which must undergo pre-processing for the mitigation of several systematic measurement errors. Typical pre-processing steps are, image background noise correction to adjust for non-specific hybridization, presence of array artifacts, washing issues or quantum fluctuations, filtering procedures to eliminate non-informative genes and data normalization [3].

Although a growing number of commercial or open source microarray data analysis software packages has already been developed, most of them are either closed black-box tools (e.g. GeneSpring™, Agilent Technologies) precluding any further intervention or modification of their inner logic, or contrary, they are open source routines, which usually perform very specific and small steps of the analysis, lacking a standard and consistent framework of parameterization for their coordinated use, given that they are also addressed to experimental biologists [4]. Although numerous software tools have been developed [5-10], a major limitation in most of them, is the absence of predefined analysis protocols or batch processes, encompassing all analysis stages from raw image analysis up to the rational derivation of DE gene lists. Exceptions to this rule exist, such as MIDAS [11], where the definition of analysis workflows in forms of batch procedures is possible and FlexArray [12], which does not support 2-colour microarrays and practically reaches up only to the point of extraction of DE gene lists. Only in its most recent versions it has started providing only basic clustering functions.

Specifically, as the field of microarray data analysis field is continuously expanding also in terms of available software, one would expect that tools developed recently, would tackle and standardize several important issues. Reduced software complexity for the novice user, multiple microarray platform support, expansion capabilities coupled with friendly interfaces, customizable ad hoc, to a wide range of users (non-expert to expert users), and predefined or interactively built analysis workflows are only few to mention. Nevertheless, recent tools either lack support for multiple microarray platforms, or they focus only on parts of the analysis, or ultimately do not properly apply data filtering steps to each specific dataset [9,12]. For example, in FlexArray, although the user is able to construct basic analysis workflows, it is not possible to combine for example the results of the MAS5 algorithm for detecting absent probesets in Affymetrix arrays, in order to directly filter the dataset and proceed to statistical analysis with a less noisy dataset. As a consequence, the user has to import two different outputs from FlexArray to spreadsheet or database software (e.g. MS Access, MySQL) in order to construct filtering queries and then re-import the data to the main program for further analysis.

Another trend in recent software [8], seems to be the inclusion of as many statistical methodologies as possible, under the umbrella of one sophisticated tool, with enhanced graphical functionalities. Even though many alternative options are provided in one tool, our experience indicates that users with limited experience, regarding the correct application of statistical methodologies, can find themselves befuddled in an endless series of analysis scenarios. This is further exacerbated by the lack of pre-defined or interactive analysis workflows. Furthermore, the complexity of this category of tools as well as of those that focus more on data management and less on data analysis [10], often necessitates the organization of special training sessions for the potential users, a rather undesired side-effect from a user group's perspective that craves for quick yet cost and time effective solutions.

Moreover, many of the current analysis packages are provided only as sets of routines, extremely unwieldy to biologists with limited programming or scripting skills. The most characteristic example is Bioconductor [4]. Despite the fact that it represents, undoubtedly, one of the richest repositories of statistical algorithms and has become, by all means, a standard for microarray data analysis, its command line interface limits its usability to many, wet-lab oriented, biological experts. Several Bioconductor packages have been assembled through Graphical User Interfaces, developed with the use of Tcl/Tk modules of R [13,14]. However, these GUI solutions lack uniformity, something that often confuses inexperienced users [8]. In addition, they are often insufficiently documented. Finally, in the case of GUI embedded solutions, lack of direct support of various raw image analysis formats is often observed, thus imposing several rounds of manual transformation first.

Taking into account the aforementioned limitations, Gene ARMADA was developed with the scope to provide both a step by step and a batch mode analysis environment, beginning from the import of raw image analysis microarray data. Thus it supports several widely used microarray image analysis formats as well as more flexible user-defined formats that ends in annotated DE gene lists, gene clusters and classifier models. Alternatively, circumventing one or more analysis steps, it can further analyze already processed data, providing the same sets of aforementioned results. Emphasis is placed equally on automation and flexibility. In this sense, analyses can be fully automated by using the batch programming module, or adjusted to ensure specific data manipulations through a very handy GUI. Depending on the user's programming skills, the open-source nature of the software allows further adjustment of analysis workflows and the addition of new analysis algorithms. Apart from the batch programming module, experienced users are also able to directly build their own workflows. This is done by using the command line version of the algorithms behind Gene ARMADA, designed to easily operate in command line mode. In its current version, Gene ARMADA fully supports the automated processing of 2 coloured cDNA and Affymetrix oligonoucleotide arrays. Yet through the incorporation of a powerful versatile custom import data editor, its workflows are easily adjusted to support the analysis of numerous types of already processed results through its versatile import editor. The program utilizes a set of gene filtering, normalization, parametric or non-parametric statistical tests, clustering and classification algorithms to analyze any number of experimental conditions and replicates. Additionally, it imports novel features in microarray signal processing, as the well established from Systems Theory concept of the signal-to-noise ratio for background correction in 2-colour microarrays, or the condition-based imputation of missing values (additional file 1)

## Implementation

### Implementation

Gene ARMADA has been implemented in MATLAB, a very powerful and globally renowned programming environment for scientific computing, and can be downloaded from . It has a complete GUI front-end created using MATLAB's language programmable user interface objects and GUIDE™. Being developed on MATLAB, Gene ARMADA is platform independent and can be used either as a MATLAB tool, when MATLAB is installed on the computer, or as a stand-alone application distributed together with MATLAB Component Runtime (MCR). Expert users are given the ability of further adjusting the analysis workflow to their own specific needs, by using or editing routines running behind the GUI, as they are designed to be used also in command line mode (MATLAB required). Gene ARMADA represents therefore a stable operating framework for versatile, transparent, seamless algorithmic integration addressing numerous facets of microarray analysis and meta-analysis.

Gene ARMADA excels among many tools which specialize either in data visualization, normalization, statistical testing, supervised or unsupervised learning and data annotation by integrating all the above, through a friendly interface. Data analysis can be performed through a predefined workflow with minimal user interaction through a batch programming module, or by performing analyses step by step upon user's requests. The following subsections summarize those features.

### Data import

Currently, the output formats of 4, widely used image analysis software, are supported (GenePix, ImaGene, QuantArray, Affymetrix) for automatic data import of both cDNA and oligonucleotide arrays. The user can also import a wealth of microarray signal files in tab-delimited text or Excel form, through a data import wizard where the user can easily import the minimal necessary parsed raw data required, for the subsequent analysis. Apart from raw data, the user can import any kind of already pre-processed data through a powerful data import wizard in order to exploit any of the rest of the functionalities of Gene ARMADA (normalization, statistical selection, clustering, annotation etc.). Through the data import wizard, the user can easily associate columns in the file (text tab-delimited or Excel) with any number of user-defined experimental conditions.

### Background correction

There are 3 options for spot background correction in 2-colour microarrays supported:

**1. Background subtraction**: In this case, the net signal for each channel is calculated, by subtracting the background signal, from the foreground estimated spot signal. Then, by taking the log_2 _ratio between channels, a measurement of expression for each spot on the array is obtained. This option represents the widely used background subtraction method for spot signal correction.

**2. Calculation of the signal-to-noise ratio**: In this case, a different approach is used, where the net signal for each channel is estimated as the ratio of the foreground estimated spot signal to the background signal. Therefore, the log_2 _ratio between channels which measures the expression levels for each spot is differently and more robustly scaled than in case (1) above, across all intensities.

**3. No background correction**: In this case, only the foreground estimated spot signal is taken into account in order to calculate the expression level of each spot on the array.

For Affymetrix arrays, RMA [15] and GCRMA [16] algorithms are available for background adjustment.

### Spot quality filtering

Poor quality spots are identified in 2-colour microarrays as follows:

Firstly, spots marked as poor either manually, or by the image analysis software are excluded. Noise sensitive genes are further isolated for each array based on 3 possible filters applied to both channels:

1. A signal-to-noise threshold filter below which noisy spots are filtered out from the slide. The default filter value is 2.

2. A filter based on the distance between the signal and background distributions; spots satisfying the inequality  are filtered out, where *S *and *B *denote the signal and background and *x *and *y *are user-defined parameters defining the strictness of the filter: as *x *and *y *increase, more spots are filtered out, as only those that have clearly separated foreground and background distributions can pass the filter. The default parameters for this filter are (*x*, *y*) = (1,2).

3. A custom filter based on the signal and background means, medians and standard deviations. The user can use the above descriptive statistics parameters to format a personal filter when imported data are known to exhibit a special or known behaviour, or simply when the user wishes to explore multiple data filtering possibilities. By providing a custom filtering option, Gene ARMADA also allows the experienced user to define a more complex filter based on the aforementioned parameters.

For Affymetrix arrays, the first step of poor quality signal identification is performed using the MAS5 detection algorithm to call present/absent probesets.

Secondly, outlier detection is performed for each spot, among the replicates of each experimental condition. Parametric or non-parametric tests are applied, to check if values follow the normal (or a continuous symmetrical) distribution with mean (median) equal to the average for this spot among all replicates. Error prone spots are excluded from the estimation of the normalization curve, to mitigate the impact of systematic measurement errors. For the case of Affymetrix arrays, this step is performed posterior to background adjustment/normalization/summarization steps.

### Normalization

Currently, 7 within slide normalization methods for 2-colour microarrays are supported: Global Mean/Median, Linear Lowess, Robust Linear Lowess, Quadratic Loess, Robust Quadratic Loess and Rank Invariant [17]. The robust Lowess/Loess versions [18] execute additional fitting iterations while readjusting each point's weight on each iteration to alleviate the impact of possible outliers. If subgrid coordinates exist for the imported array type, subgrid normalization is also possible and there is also full support for dye-swap experiments. Between-slides normalization is part of the statistical analysis workflow and can be performed using Quantile normalization [19] or MAD centering [20]. For Affymetrix arrays, either Quantile or Rank Invariant normalization can be performed.

### Statistical selection

Selection of DE genes is implemented by using a graphical interface where the user can define (or use default values) several parameters and contrasts for each analysis, performed in a workflow manner. It starts with the calculation of the *Trust Factor *(TF) which is defined for each gene as the ratio of its number of qualified replicates after the filtering step, to the total number of replicates, for each condition. The definition of the TF according to user's requests sets a reliability threshold, excluding genes from further analysis. The imputation of missing values for a gene is based on the average expression of the remaining present values of that gene from the same experimental condition or the kNN imputation algorithm [21]. It can be performed before or after between-slides normalization (default is after). Differentially expressed genes are acquired using parametric (ANOVA, t-test) or non-parametric (Kruskal-Wallis) statistical tests. Multiple testing correction is performed by controlling the FWER [22] or the FDR [23,24]. After the completion of each contrast, the user is prompted to accept the results or repeat the analysis using different parameter sets.

### Data clustering and classification

Currently, Gene ARMADA supports 3 fully customizable clustering algorithms: Hierarchical and k-means clustering based on the Statistics Toolbox of MATLAB and fuzzy C-means clustering [25]. A novel contribution is the automated derivation of the optimal number of clusters for the appropriate classification/partition of the dataset in gene groups. This is performed through the utilization of the Gap statistic [26], in a routine implementation which, by using distance metrics comparison with different reference gene signal distributions and iteration, selects the optimal number of clusters. Principal Component Analysis [27] is also provided for the derivation of the critical features of datasets. Supervised classification can be performed using Discriminant Analysis, k-Nearest Neighbors or Support Vector Machines [28] using the OSU-SVM toolbox [29], which supports multiclass classification and is based on the widely utilized libsvm [30]. Classifier parameterization can be performed, through an implemented interface for the appropriate tuning of each algorithm. This interface includes flexible dialogs where the user can define several parameters to be tested (or accept the defaults), as well as several classifier performance plots and detailed output depicting statistics concerning the performance of each classifier.

### Visualization tools

Gene ARMADA provides a variety of visualization, exploration and quality assessment graphs, such as array spatial images for several spot quantitation types, normalized and un-normalized expression values, individual and array versus array plots for several quantitation data, expression distribution histograms, boxplots, MA, volcano and expression profile plots, clustering heatmaps and classifier evaluation plots. Most graphs are interactive and users can select and export data directly in various graphic formats. The variety of provided visualization tools can be utilized for numerous purposes, encompassing among others, quality control and analysis (e.g. normalization) assessment, in order to aid in threshold and parameter estimation for several procedures, like statistical analysis, clustering and supervised classification.

### Data export

The results of the analyses can be exported in tab delimited or Excel file formats, or as .mat files so that the experienced user can further process the data with other MATLAB built-in algorithms. The output is customizable through an export wizard, allowing either basic data export for exploratory purposes, or the export of rich specific formats for direct use with other tools, such as GenMAPP [31] for mapping gene expression on cellular pathways, or for Gene Ontology Terms based statistical analysis, through the utilization of the software package named RankGO [32] (available for download from ). Outputs can be massively annotated through an annotation module.

### Batch programmer

Gene ARMADA offers a batch analysis programming module which can be used to schedule several analysis rounds, encompassing different methods and parameters. This feature offers the user the ability to simply import the data and then pre-define multiple analysis workflows without having to personally monitor the whole procedure. Upon completion of the execution of the batch processing, the user can collect the multiple outputs, and directly compare the differences among different method/parameter sets. Specifically, through the batch scheduling module, users can set: i) filtering and normalization steps, ii) define multiple analysis dispatches of multiple statistical comparisons among different experimental condition and replicate sets and also iii) define different clustering procedures. In this way, they are capable of conducting multiple algorithm exploration tests at once. The batch analysis options can be saved in a separate file, so that they can be easily recalled. The batch analysis results can be saved as a Gene ARMADA project so that they can be later loaded into the main program for visual data explorations or exporting in other storage formats.

### Data analysis structures

The data analysis structures implemented in Gene ARMADA allow users to define several experimental condition and slide replicate subsets in distinct analysis objects, each carrying the essential data required to maintain and/or easily recall analysis steps performed for each analysis run. This implementation allows the conduct of different comparisons without repeating the computationally intensive normalization task when not necessary. In addition, it enables the examination and assessment of the performance of different filtering and normalization steps, with respect to dataset specific optimized tuning of the algorithmic processing pipeline.

## Results and discussion

**Figure 1:**
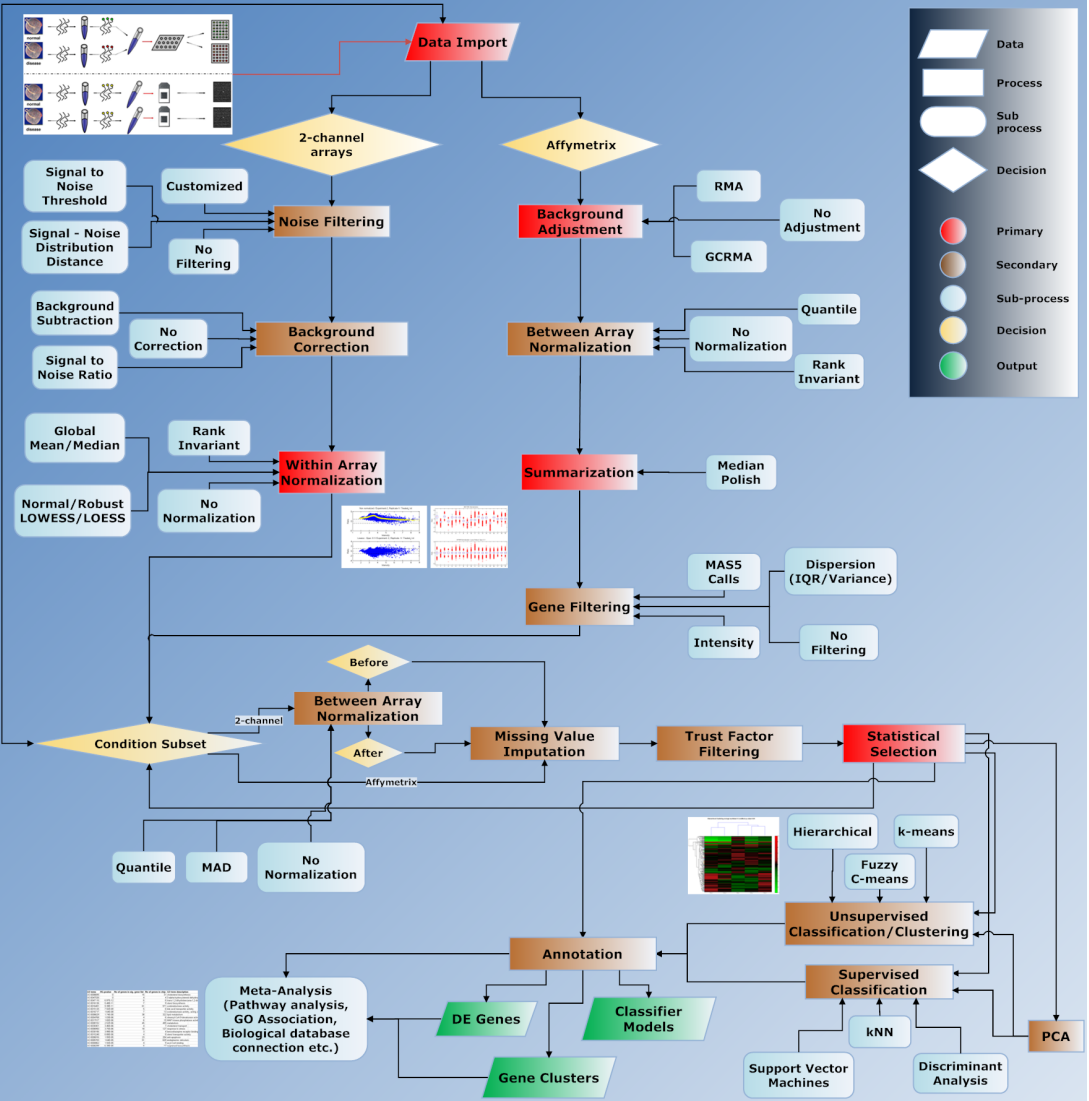
**Gene ARMADA analysis workflow**. Detailed flowchart of data analysis procedure followed by Gene ARMADA, depicting also available analysis algorithms and their application position in the overall workflow.

Gene ARMADA aims to provide investigators with a complete, open-source, flexible and handy platform for both cDNA and oligonucleotide (Affymetrix) microarray data analysis. It encompasses all levels of analysis to easily interpretable and simply manipulated gene lists. It addresses a very wide category of users, practically meeting all levels of competences regarding analysis and interpretation of microarray experiments, by providing a friendly software environment equipped with a graphical user interface, plus automated workflows for the analysis of microarray experiments together with absolutely open source routines, adjustable to meet specific data processing needs. The adoption by Gene ARMADA of a specific analysis protocol (Figure 1), represents one of the major strengths of it, as it provides a solid base of standardization of the microarray experiments processing, where the data handling steps among different experiments can be boiled down to very specific descriptions that unequivocally characterize the type of transformations applied to various data populations. Additionally, Gene ARMADA integrates several major steps of microarray data analysis (2-colour and single dye) under one tool, and represents a connection point for annotation tasks, as well as customizable integration of numerous other, microarray data analysis tools.

**Figure 2:**
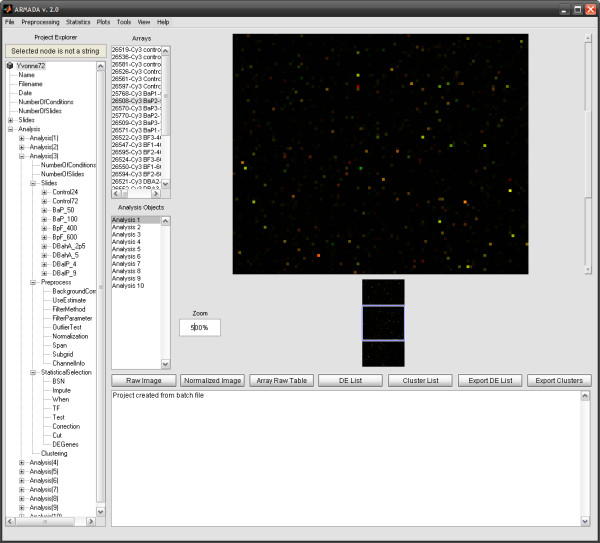
**Gene ARMADA main window**. All available choices and controls can be easily accessed through a carefully designed and friendly user interface. The main window contains also shortcuts to main functionalities and the user has the choice of getting a variety of informative messages and short reports concerning the analysis procedure.

The total computational cost of an experiment depends heavily on the amount of arrays to be analyzed, as well as the type of data processing workflow selected for implementation (e.g. while global median normalization is preformed in less than a second, robust LOESS requires a much larger amount of time due to local data processing). Yet the inherently parallel structure of the Gene ARMADA analysis workflows renders it an amenable environment for parallel computing. In this sense, Gene ARMADA represents the prototype, upon which the development of a web-based microarray analysis application, integrating in its programming, principles of Grid computing, GRISSOM (, **GR**ids for **I**n **S**ilico **S**ystems Bi**O**logy and **M**edicine) has been developed. Concerning the program use, apart from the graphical interface, the user can program several analysis rounds through the batch programming module. A snapshot of the program is illustrated in Figure 2.

It is to be noted that the signal-to-noise ratio implemented background correction method that utilizes the signal-to-noise content of a signal, is an established notion in systems theory and image processing [33]. It represents a pre-processing step, in line with the perception of the experimentalist about signal quality, emphasizing in the signal strength compared to noise. Given the reported controversy on background subtraction [34], this approach is critical, especially regarding weak signal datasets, whereas a majority of spot signals is close or even below the background levels. To our knowledge, there is no other analysis tool using the signal-to-noise ratio for background correction for 2-colour microarrays.

Finally, it should be also stressed here the novelty regarding the automated derivation of the optimal number of clusters, for the appropriate classification/partition of the dataset to gene families through the utilization of the Gap statistic [26]. This is another crucial feature for automated clustering procedures. Gene ARMADA has been successfully used so far for the analysis of several microarray datasets, including datasets concerning interstitial pulmonary fibrosis [32] and gene expression profiling of progestin-induced canine mammary hyperplasia and spontaneous mammary tumors [35]. It has also been used for processing microarray datasets concerning gene expression profiling of mouse liver and lung tissues after treatment with different dosages of Polycyclic Aromatic Hydrocarbons (S. Kyrtopoulos, personal communication). Additionally it has been used for clustering RNA Polymerase II occupancy in 2 ChIP-Seq datasets [36,37] from Next Generation Sequencing experiments, thus proving the generic design of the Gene ARMADA algorithmic modules, which render it adjustable to versatile processing tasks. Software efficiency and good functionality has also been extensively tested with several microarray datasets retrieved from public repositories such as ArrayExpress and GEO.

## Conclusion

Gene ARMADA aspires to provide a unified, automated and flexible platform for both 2-colour and Affymetrix oligonucleotide microarray data analysis and interpretation. It is programmed in MATLAB, exploiting elements from the Statistics and Bioinformatics Toolboxes and offering friendly integration with numerous other tools. Gene ARMADA has been successfully used to process several microarray datasets and has been tested with multiple public datasets some of which are available for download at .

## Availability and requirements

**Home page**: .

**Operating system**: Windows, if used as a stand-alone application or platform independent, if used under MATLAB.

**Requirements**: If used under MATLAB, Statistics and Bioinformatics Toolboxes should be also installed. If used as a stand-alone application, MATLAB Component Runtime (MCR) 7.6 is required, which is distributed with the application installer from the software's home page. In order to parse Affymetrix .CEL and library files, the Affymetrix GDAC Runtime Libraries are required, which can be found under the Bioinformatics Toolbox if MATLAB is present on the installation machine, or they are distributed with the stand-alone application installer from the software's home page. In some machines, .NET framework 2.0 can be also required.

**License**: Gene ARMADA is distributed under the Academic Free License (AFL) v.3.0. The software is available to all users without registration.

**Further information**: All versions of the software (MATLAB or stand-alone application) together with an analytical user's guide, installation instructions, several video tutorials, screenshots and several test datasets are available for download at the software's homepage.

## List of abbreviations

The abbreviations used throughout the article are: ARMADA: Automated Robust MicroArray Data Analysis; DE: Differentially Expressed; GUI: Graphical User Interface; MCR: MATLAB Component Runtime; TF: Trust Factor; FWER: Family-Wise Error Rate; FDR: False Discovery Rate; AFL: Academic Free License.

## Competing interests

The authors declare that they have no competing interests.

## Authors' contributions

AC conceived the idea of software implementation and the analysis workflow, designed the structure of the analysis pipeline, contributed to the implementation of the routines, coordinated the development of the software, tested the software and contributed to the drafting of the manuscript. PM designed and implemented the GUI, contributed to the implementation of the routines, tested the software, created the software's website and documentation and contributed to the drafting of the manuscript. FNK contributed to the evaluation of the software regarding the biological data management, and revised the manuscript. All authors have read and approved the final manuscript.

## Supplementary Material

Additional file 1 **Source code of Gene ARMADA**. Gene ARMADA's MATLAB source code. The file should be extracted using a suitable program (e.g. Winzip, WinRAR, gzip or 7-Zip) and the extracted files and folders should be placed in MATLAB's path. The program can then start by typing ARMADA in MATLAB's command line. Click here for file
