# Supplementary material for: Gene ARMADA: an integrated multi-analysis platform for microarray data implemented in MATLAB
Source: BMC Bioinformatics. 2009 Oct 27;10:354. doi: 10.1186/1471-2105-10-354 (PMC2771024; doi:10.1186/1471-2105-10-354)
Supplement: Additional file 1 — Source code of Gene ARMADA. Gene ARMADA's MATLAB source code. The file should be extracted using a suitable program (e.g. Winzip, WinRAR, gzip or 7-Zip) and the extracted files and folders should be placed in MATLAB's path. The program can then start by typing ARMADA in MATLAB's command line. [file 1471-2105-10-354-S1.ZIP › ARMADA/ARMADA user guide.pdf]

ARMADA

Automated Robust MicroArray Data Analysis

version 1.1

User's manual

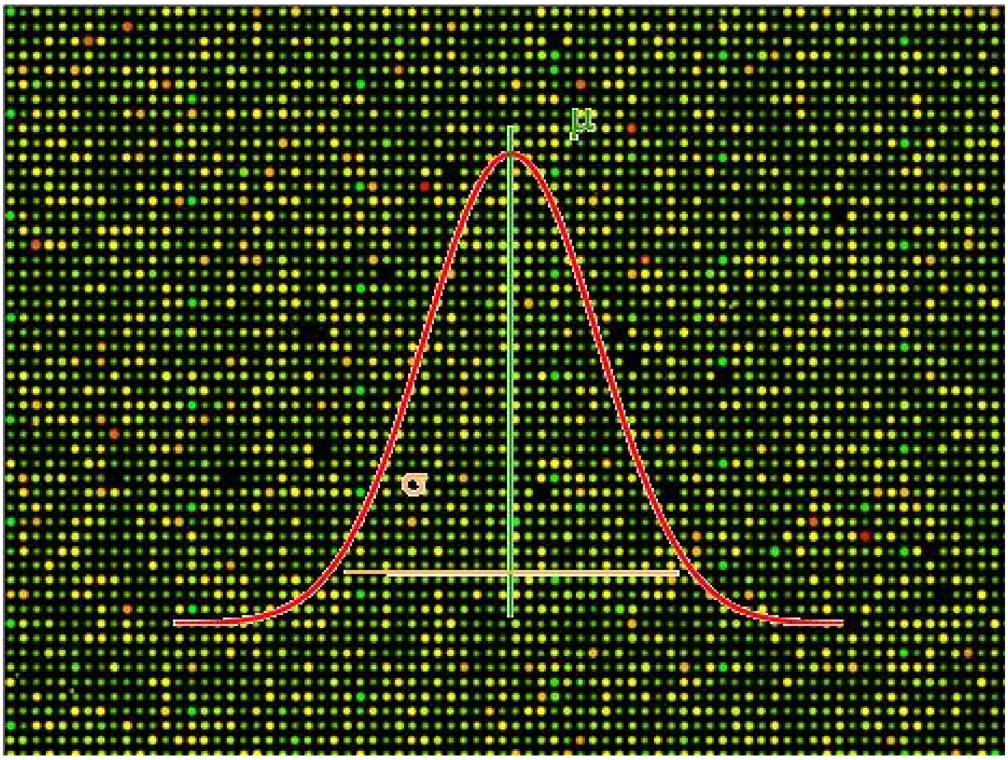

Metabolic Engineering and Bioinformatics Group, Institute of Biological Research and Biotechnology

National Hellenic Research Foundation

©2008

Contents

Contents ... 3

1. Overview ... 6

  1.1. Program overview ... 6

  1.2. Release changes ... 7

  1.3. Bug reporting ... 8

2. Basic Operations ... 9

  2.1. Installation requirements and instructions ... 9

  2.2. Creating a new project ... 9

  2.3. Opening a previously saved project ... 10

  2.4. Saving a project ... 10

  2.5. Importing data ... 10

    2.5.1. Importing data directly from image analysis software – supported program outputs ... 10

    2.5.2. Importing data directly from image analysis software – text tab delimited files ... 13

    2.5.3. Importing already processed data ... 15

  2.6. Exploring data – main window ... 17

    2.6.1. ARMADA's main window ... 17

    2.6.2. History textbox ... 18

    2.6.3. Tree view ... 18

    2.6.4. Arrays list ... 20

    2.6.5. Analysis Object List ... 21

    2.6.6. Raw Image ... 22

    2.6.7. Normalized Image ... 22

    2.6.8. Array Raw Table ... 23

    2.6.9. Normalized List ... 23

    2.6.10. Differentially Expressed genes List ... 24

    2.6.11. Cluster List ... 25

    2.6.12. Reports ... 25

    2.6.13. Deleting analysis objects ... 26

3. Preprocessing Data ... 27

  3.1. Selecting subjects of experimental conditions ... 27

  3.2. Background Correction ... 28

  3.3. Spot quality filtering ... 30

  3.4. Normalization ... 34

4. Statistical Operations ... 40

4.1. Statistical Selection 40
4.2. Fold Change Calculation 44
4.3. Clustering 45
4.3.1. Hierarchical clustering 45
4.3.2. k-means clustering 47
4.3.3. Fuzzy C-means clustering 49
4.4. Classification 51
4.4.1. (Linear) Discriminant Analysis 52
4.4.1.1. (Linear) Discriminant Analysis – Tuning 52
4.4.1.2. (Linear) Discriminant Analysis – Classifying 55
4.4.2. k–Nearest Neighbors 56
4.4.2.1. k–Nearest Neighbors – Tuning 57
4.4.2.2. k-Nearest Neighbors - Classifying 61
4.4.3. Support Vector Machines 62
4.4.3.1. Support Vector Machines – Tuning 62
4.4.3.2. Support Vector Machines - Training 65
4.4.3.3. Support Vector Machines - Classifying 66
5. Graphical data exploration 67
5.1. Array Images 67
5.2. Normalized and Un-normalized images 70
5.3. Array plots 71
5.4. MA Plots 74
5.4.1. MA plots before normalization 75
5.4.2. MA plots after normalization 76
5.4.3. MA plots before and after normalization 77
5.4.4. Subgrid MA plots 78
5.5. Expression Distributions 80
5.6. Boxplots 84
5.7. Volcano Plots 87
5.8. Expression Profiles 90
6. Exporting Data 95
6.1. Exporting gene lists 95
6.2. Exporting gene cluster lists 97
6.3. Exporting figures 98
6.4. Exporting to .mat files 98
7. Other Tools 102
7.1. The Principal Component Analysis tool 102

7.2. The Gap Statistic tool 104
7.3. The Batch Programmer 107
7.4. The Annotator 109
References 111
Appendix A: Input file formats 112
A.1.Raw data – Image analysis software output and tab delimited files 112
A.1.1. QuantArray file format 112
A.1.2. ImaGene file format 113
A.1.3. GenePix file format 114
A.1.4. Text tab delimited files 115
A.2. Processed data 115
A.3. Files used for classification 116
A.3.1. New sample files 117
A.3.2. External class prior files for DA classification 117
A.3.3. External kernel parameters files for SVM tuning 117
Appendix B: MATLAB's figure controls 118
B.1. Figures 118
B.2. Figure toolbars 119
Appendix C: Multiple testing correction issues 121
Appendix D: Distance metrics and linkage algorithms 122
D.1. Distance metrics 122
D.2. Linkage algorithms 123

1. Overview

1.1. Program overview

Microarray technology allows gene expression profiling at a global level by measuring mRNA abundance. ARMADA (Automated Robust MicroArray Data Analysis) is a MATLAB implemented program with a graphical user interface (GUI) which performs all steps of typical microarray data analysis; starting from importing raw data from several image analysis software outputs as well as text tab delimited files or already processed data that need to undergo statistical testing, ARMADA continues with processes including noise filtering, spot background correction, data normalization, statistical selection of differentially expressed genes based on parametric or non parametric statistics, cluster analysis based on several widely used clustering methods (Hierarchical, k-means, Fuzzy C-means) and annotation steps, resulting in detailed lists of differentially expressed genes and formed clusters. Along with the user friendly interface, ARMADA offers a variety of visualization options (MA plots, boxplots, array images, clustering heatmaps etc), a module which allows multiple analyses to be performed in batch mode under a specific analysis workflow and an annotation tool. Emphasis is given to the output data format which is fully customizable and contains a substantial amount of useful information such as detailed normalized and unnormalized expression values for each gene on each slide replicate along with several statistics concerning expression values for each experimental condition. The ARMADA output files can be easily imported in a spreadsheet like software such as MS Excel or in a database for further processing and storage and the analysis results can be saved as .mat files for further possible processing with MATLAB's built-in algorithms.

Depending on the user's programming experience and analysis preferences, ARMADA can be used to perform analyses step by step through the GUI of the system or as an automated analysis pipeline (by using the batch programming module). For the most experienced user, ARMADA can also be invoked directly from MATLAB's command window, as the main routines that perform the analysis behind the GUI are designed to run also individually in command line mode with specific arguments (the user should see help inside .m files to perform command line analysis). ARMADA is a completely open source MATLAB based platform and the user may alter, adjust or extend each of the main functions or create new routines according to specific needs. It should be noted that ARMADA can be used in command line mode only if MATLAB is present on the computer where ARMADA is installed. Otherwise, the program is distributed with MATLAB Component Runtime (MCR) and MATLAB is not required on the installation machine.

In order to use ARMADA under MATLAB or in command line mode (and thus be able to export results in MATLAB's workspace for further processing with built-in algorithms), MATLAB 7.3 (R2006a) or higher should be installed on the target computer. In this case the platform consists

only of MATLAB routines and not compiled files and is platform independent. After downloading the routines, the user should place them in a folder of preference maintaining the structure in the compressed file and then add this folder including its subfolders to the MATLAB path. If MATLAB is not present in the target computer, MATLAB Component Runtime (MCR) 7.6 is required. The MCR is included in the program installer which can be downloaded from (URL here). If MCR 7.6 was previously installed for other reasons, then it does not have to be installed again. Note that in this case the program will work ONLY with MCR 7.6 and NOT with older or newer versions of the MCR.

One of the main advantages of ARMADA is that it offers an analysis workflow by not allowing the user to do "anything" at "anytime", a feature that will prove valuable especially for users mostly with biological background. The purpose of this user's guide is to provide insights on the use of the platform and explain its capabilities as simple as possible in the eyes of a biologist with little programming experience or little experience in statistical computing. If some points are unclear or not explained as explicitly as expected, please provide feedback and help the developers perform better on later versions of ARMADA. Please report feedback (comments, suggestions or possible bugs and malfunctions) to Panagiotis Moulos (pmoulos@eie.gr).

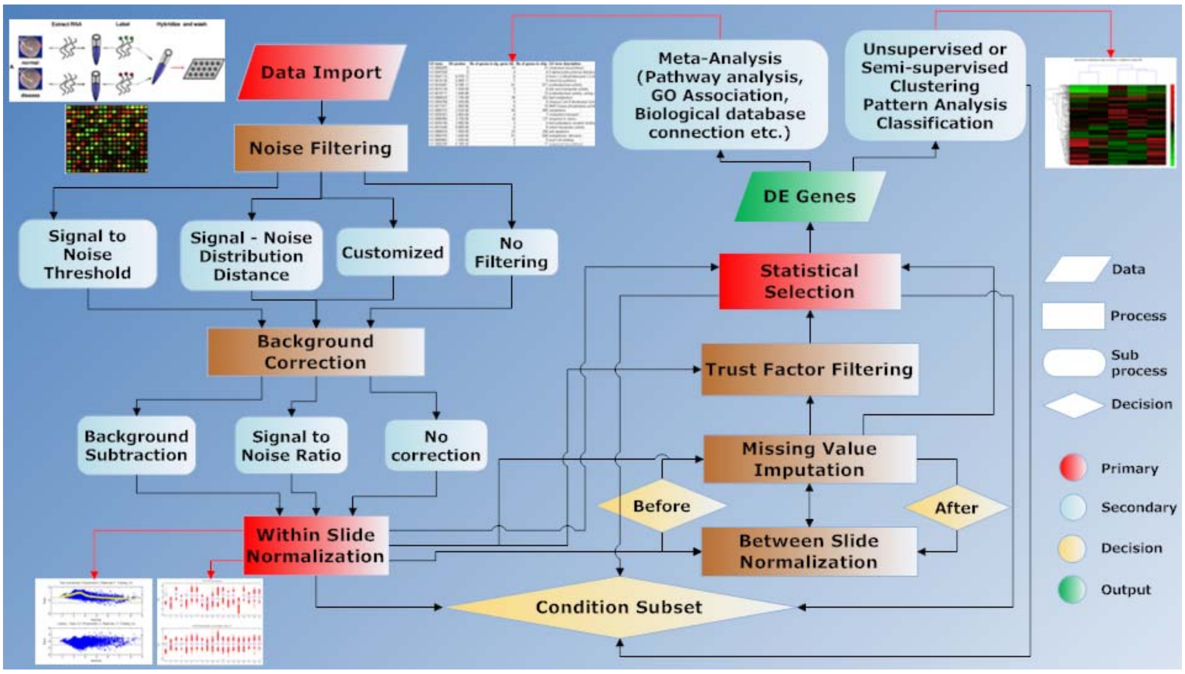

Analysis workflow of ARMADA

1.2. Release changes

The following section is a description of the additions made in version 1.1 of ARMADA compared to the previous version, 1.0:

- Users can now import external already processed data directly for clustering or supervised learning training without having to perform statistical selection first.

- Complete dye-swap experiment support, implemented during the normalization procedure, under the Normalization preferences.
- Added single array plots for several image quantitation types (e.g. Channel 1 vs Channel 2 etc.). Data are selectable and exportable as in MA or Volcano plots.
- Added array vs array plots for several measurements (e.g. log_2 ratio, dye quantitation etc.). Data are selectable and exportable as in MA or Volcano plots.
- Several bug fixes.

1.3. Bug reporting

If the user wishes to report a bug, it is recommended that the exact error message is included in the report (a simple screenshot of the error using a simple screen capturing program or simply hitting the button 'PrtScn' on the keyboard would be enough) together with a small description on what process the user tried to perform. If the bug appears during the data import process and if the problem is not solved by following the import instructions described in this user's guide exactly, it is recommended that a sample of the files used for import is included in the report.

2. Basic Operations

This section of the user guide presents the installation requirements and installation process of ARMADA and how the user can perform basic operations such as creating and saving and opening projects, opening new session windows and importing data to ARMADA in various ways.

2.1. Installation requirements and instructions

In order to run ARMADA, the user must have at least MATLAB 7.3 (R2006a) or higher, or the MATLAB Component Runtime (MCR) 7.6 (not higher) installed on his computer. ARMADA can be downloaded from (site here) as MATLAB routines for the users who are experienced with MATLAB and have at least MATLAB 7.3 (R2006a) installed on their computer, or as an executable installer file which also contains MCR 7.6. Example datasets can also be downloaded from the above site. If the user chooses to download ARMADA as MATLAB routines, the downloaded file should be unzipped in a location of user's preference and then the specific location must be added to MATLAB's path including its subfolders (In MATLAB, File → Set Path… → Add with Subfolders…). If the user chooses to download ARMADA as an executable installer file, the instructions provided through the installation process should be followed carefully. ARMADA is distributed under the Academic Free License, version 3 (http://www.opensource.org/licenses/academic.php).

2.2. Creating a new project

To create a new project, the user should click on **File → New → New Project** (or **Ctrl + N**) and then the following window will appear:

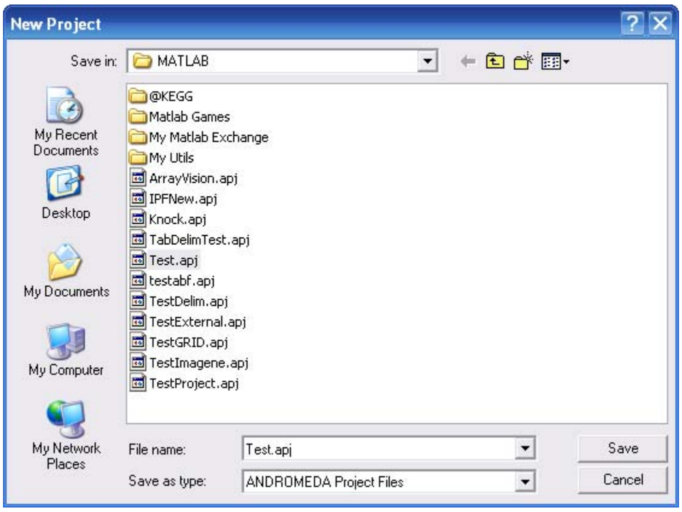

The user is prompted to fill the field **File name** with the desired project name and click **Save** for the new project to be created. For a new ARMADA session window, the user should click **File → New → New Session** (or **Ctrl + I**).

2.3. Opening a previously saved project

To open a previously saved project, the user should click **File** → **Open** (or **Ctrl** + **O**) and then the following window will appear:

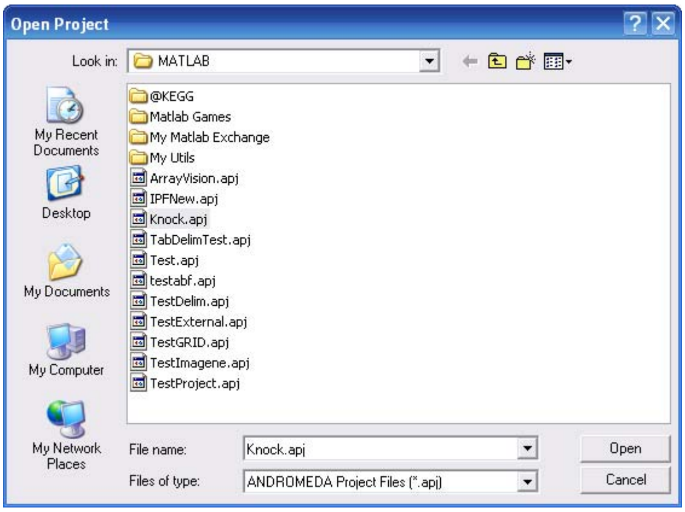

From there, the user should select a previously created project and click **Open**.

2.4. Saving a project

To save the current project, the user should click **File** → **Save** (or **Ctrl** + **S**). To save a project under a different filename, the user should click **File** → **Save As**, enter a new filename on the project and click **Save**.

2.5. Importing data

The following sections describe how data files derived from the image analysis programs supported or from text tab delimited files (e.g. downloaded from public repositories) can be imported for processing to ARMADA. The user should note that when data import is completed properly, no further data importing is possible in the same project. This is part of ARMADA workflow and if the user wishes to import other data in ARMADA, a new project should be created.

2.5.1. Importing data directly from image analysis software – supported program outputs

The first step of analyzing a dataset consists of proper import of the image analysis software output files or text tab delimited files containing image quantitation data for each spot on the array. To import a dataset in the current project, the user should click **File** → **Data Import** → **Raw image data** and the following window will appear:

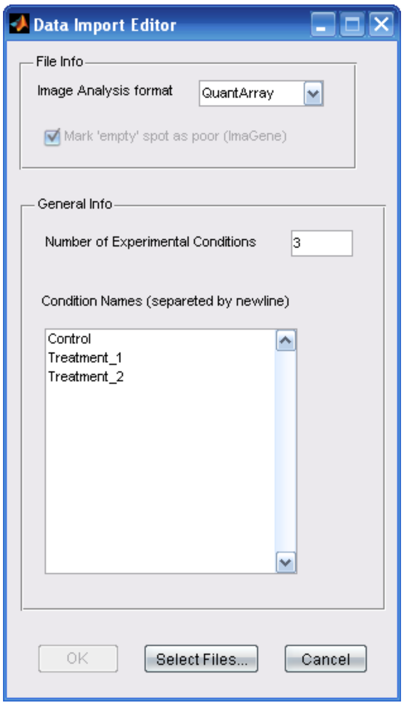

In this data import wizard, the user is prompted firstly to choose the software which was utilized to process raw images and create the files containing image quantitation data. Currently, 4 software formats are supported: QuantArray (Perkin Elmer, Inc.), ImaGene (BioDiscovery, S.A.), GenePix (Molecular Devices) and simple text tab delimited format files containing image quantitation elements coupled with (optionally) spatial information on how the spots are distributed on the microarray. This format is useful when datasets downloaded from public databases such as ArrayExpress (www.ebi.ac.uk/arrayexpress/) or Gene Expression Omnibus (GEO) (http://www.ncbi.nlm.nih.gov/geo/) are to be imported. The user can use this option also when the dataset images have been processed with software not supported by ARMADA after certain manual manipulation first (the user should see Appendix A for further information on file formats). Note that if the dataset files have been produced with ImaGene software, the user can check the **Mark 'empty' spot as poor (ImaGene)** option. If this option is activated, then spots that are flagged by the user or the software as empty will be treated by ARMADA as poor quality spots and excluded from analyses. This option is included in case the user wishes to include empty spots in the analysis, for example as an estimation of noise in the images of the experiment.

As a second step, the user is prompted to enter the number of different conditions of the experiment in the field **Number of Experimental Conditions** (e.g. if the experiment includes the experimental factors Control, Treatment 1, Treatment 2, the user should enter 3). Next, in the field **Condition Names**, the user should fill in the names of the experimental conditions (e.g. Control, Treatment_1, Treatment_2). It should be noted that many special characters[1] are not allowed and the names should not start with numbers (e.g. the condition names "Control.1*T" or "1Control" will

[1] These characters are the following: < > / \ , ; "" [ ] " | ? . ! @ # $ % ^ & * ( ) - + = ` ~ or white spaces.

generate an error message and will be automatically replaced by valid names). After properly setting the parameters described above, the user should click on the button **Select Files…** and will be prompted to select the directory where the data files are placed (it is not necessary to have all files in one directory; this step exists for user's convenience). The following window will appear that will help the user select the files of the experiment:

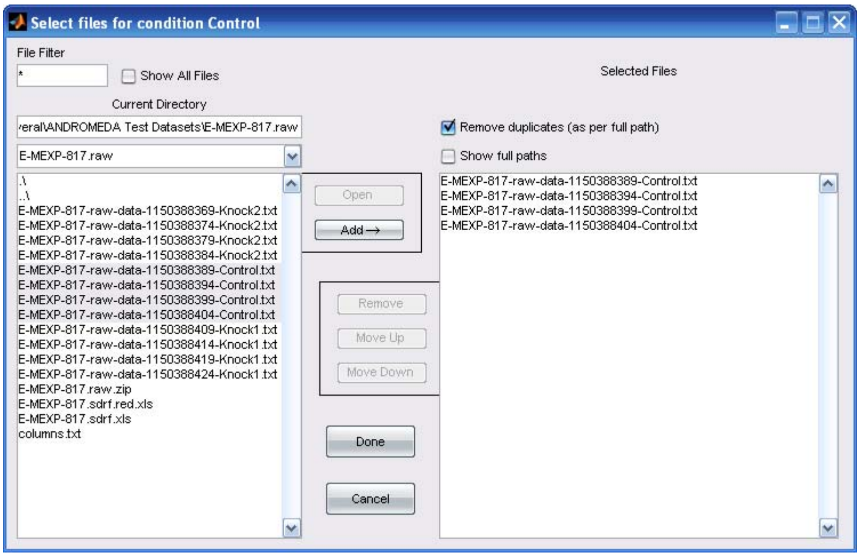

This window will appear as many times as the number of the experimental conditions in the project. Each time the user will be prompted to select the files for the condition with name displayed in the window title (e.g. "Select files for condition Control"). This file selection window gives certain control over the file names and what is displayed (e.g. the user can filter what is displayed by a regular expression (the user should see for example http://www.regular-expressions.info/), change the directory or display the full paths of the selected files). In the case of importing from ImaGene output, the user should also take into account that ImaGene files are produced in pairs (one file for Cy3 – 1st channel and one file for Cy5 – 2nd channel). Thus, the files are also selected in pairs. ARMADA distinguishes the channels for ImaGene files by searching for the text 'Cy3' or 'Cy5' in the filenames. The user should make sure that this string exists in the filenames and that each file corresponds to the proper channel. After selecting the array files for each condition, the user should click **Done**.

To finish importing the dataset, click **OK** in the Data Import wizard window. If the user has chosen to import direct output files from one of the supported image analysis programs, they will be automatically imported for analysis in ARMADA. The case of tab delimited text files that contain the image quantitation types is explained in the next paragraph.

Important notice: when importing from GenePix, the user should make sure that channel 1 (or Cy3 or 'Green') corresponds to the 532nm wavelength and that channel 2 (or Cy3 or 'Red') corresponds to the 635nm wavelength because ARMADA assigns channels in this way. In case of the opposite, the user should correct for this by selecting 'Cy5 is channel 1' at the **Channel – Dye correspondence** list in the normalization preferences window (section 3.4).

2.5.2. Importing data directly from image analysis software – text tab delimited files

In the case of selecting to import image quantitation data from text tab delimited files (e.g. downloaded from a public repository), after clicking **OK** in the Data Import wizard window, the following window will appear:

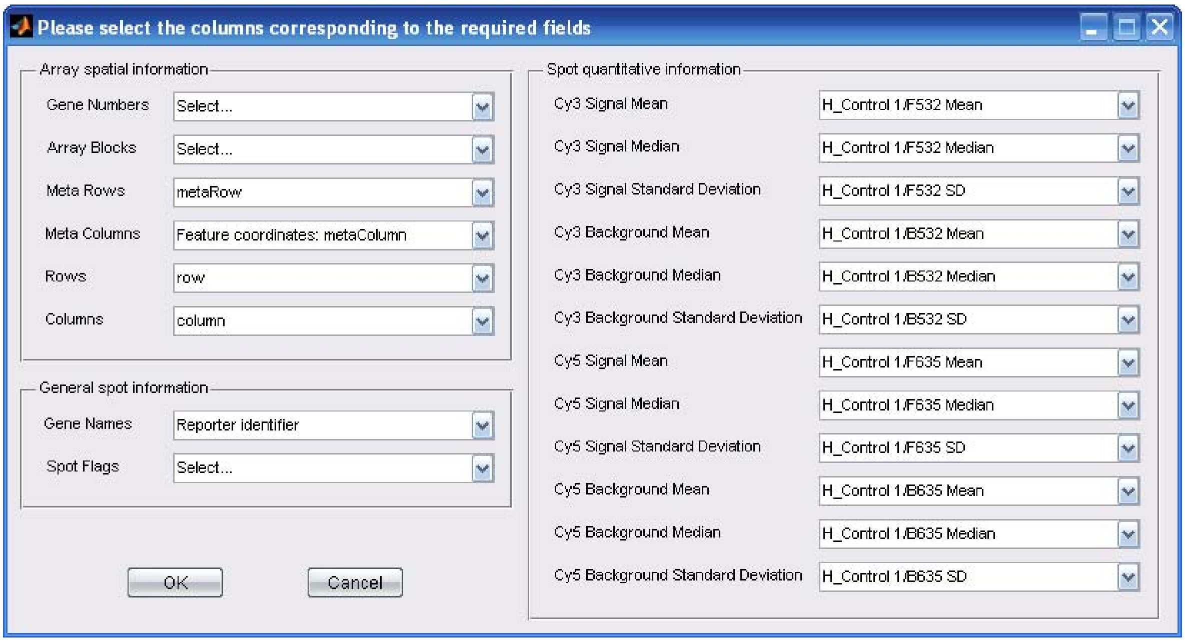

Each list contains all the column headers of the first file out of the whole file set imported by pressing the **Select Files…** button in the Data Import wizard. Therefore the user should have checked before this step that all the text files in the dataset have *exactly* the same format (e.g. there isn't a file where the column with name 'Row' is placed 4th from the beginning of the file while in all other files it is placed 3rd). The following table explains the content of each required field.

**Field Name**	**Description**	**Optional**
Gene Numbers	A unique gene numbering present on the microarray slide (e.g. the column "Gene Number" on QuantArray files). This attribute allows the unique gene identification in the case of multiple clones of a transcript present on the slide. If not given, it will be assigned automatically.	Yes
Array Blocks	The column with numbers from 1 to the number of blocks into which the probes on the array are organized. This attribute helps in the reconstruction of array images. If not given and the slide is organized in blocks, the Meta Rows and Meta Columns attributes should be provided.	Yes
Meta Rows	Probe meta-coordinates[2] (row). This attribute helps in the reconstruction of array images. If not given and the slide is organized in blocks, the Blocks attribute should be provided.	Yes
Meta Columns	Probe meta-coordinates (column). This attribute helps in the reconstruction of array images. If not given and the	Yes

[2] If not given properly, probe meta-coordinates as well as slide probe coordinates could generate errors. If the user is not sure about the validity of these attributes they should not be provided. ARMADA will still produce an image, but it will not represent the distribution of probes on the array.

	slide is organized in blocks, the Blocks attribute should be provided.	
Rows	Probe coordinates (row) in each block or simple probe coordinates. This attribute helps in the reconstruction of array images. If given with meta-coordinates, it helps in the reconstruction of blocks in the image. If meta-coordinates are not given, this attribute (together with "Columns" are taken to be the array coordinates.	Yes
Columns	Probe coordinates (column) in each block or simple probe coordinates. This attribute helps in the reconstruction of array images. If given with meta-coordinates, it helps in the reconstruction of blocks in the image. If meta-coordinates are not given, this attribute (together with "Rows" are taken to be the array coordinates.	Yes
Gene Names	The column containing (ideally) unique gene identifiers (usually provided by manufacturers).	No
Spot Flags	The column containing flags (manually or automatically produced by the image analysis software used marking poor spots). Note that this column should contain only one's (1's) and zero's (0's) with 1 representing good spots while 0 poor quality spots. If you wish to provide this attribute make sure that your files contain proper flags as defined above. If not, do not provide this attribute and the internal filters of ARMADA will be used to mark poor quality spots.	Yes
Cy3 Signal Mean	The column containing signal quantitation for each spot for the 1st channel. The title Cy3 is indicative and taken by the fact that the reference samples in 2-coloured microarray experiments are labelled with Cyanine 3 ("green"). In any case, this attribute should contain the foreground intensities for the reference dye (channel). The title "Mean" is indicative. It depends on the quantitation algorithm of each image analysis software. However, this attribute is mandatory and should contain the main signal quantitation for each spot.	No
Cy3 Signal Median	The column containing the median of signal quantitation for each spot of the 1st channel[3].	Yes
Cy3 Signal Standard Deviation	The column containing the standard deviation of signal quantitation for each spot of the 1st channel[4].	Yes
Cy3 Background Mean	The column containing background contamination quantitation for each spot for the 1st channel. The title Cy3 is indicative and taken by the fact that the reference samples in 2-coloured microarray experiments are labelled with Cyanine 3 ("green"). In any case, this attribute should contain the background intensities for the reference dye (channel). The title "Mean" is indicative. It depends on the quantitation algorithm of each image analysis software. However, this attribute is mandatory and should contain the main background quantitation for each spot.	No
Cy3 Background Median	The column containing the median of background quantitation for each spot of the 1st channel.	Yes

[3] It should be noted that if the "Median" attributes are not given, ARMADA's filtering methods will not be available at full extent.
[4] It should be noted that if the "Standard Deviation" attributes are not given, ARMADA's filtering methods will not be available at full extent.

Cy3 Background Standard Deviation	The column containing the standard deviation of background quantitation for each spot of the 1st channel.	Yes
Cy5 Signal Mean	The column containing signal quantitation for each spot for the 2nd channel. The title Cy5 is indicative and taken by the fact that the reference samples in 2-coloured microarray experiments are labelled with Cyanine 5 (“red”). In any case, this attribute should contain the foreground intensities for the sample dye (channel). The title “Mean” is indicative. It depends on the quantitation algorithm of each image analysis software. However, this attribute is mandatory and should contain the main signal quantitation for each spot.	No
Cy5 Signal Median	The column containing the median of signal quantitation for each spot of the 2nd channel.	Yes
Cy5 Signal Standard Deviation	The column containing the standard deviation of signal quantitation for each spot of the 2nd channel.	Yes
Cy5 Background Mean	The column containing background contamination quantitation for each spot for the 2nd channel. The title Cy5 is indicative and taken by the fact that the reference samples in 2-coloured microarray experiments are labelled with Cyanine 5 (“red”). In any case, this attribute should contain the background intensities for the sample dye (channel). The title “Mean” is indicative. It depends on the quantitation algorithm of each image analysis software. However, this attribute is mandatory and should contain the main background quantitation for each spot.	No
Cy5 Background Median	The column containing the median of background quantitation for each spot of the 2nd channel.	Yes
Cy5 Background Standard Deviation	The column containing the standard deviation of background quantitation for each spot of the 2nd channel.	Yes

After filling all the necessary fields by choosing from the lists, the user should click **OK**. Data importing should begin immediately.

2.5.3. Importing already processed data

If data which have been preprocessed with another analysis tool or downloaded from a public repository have to be imported to the project, the user should click **File → Data Import → Processed data** and the following window will appear, prompting the user to select a text tab delimited or MS Excel file containing the data to be imported:

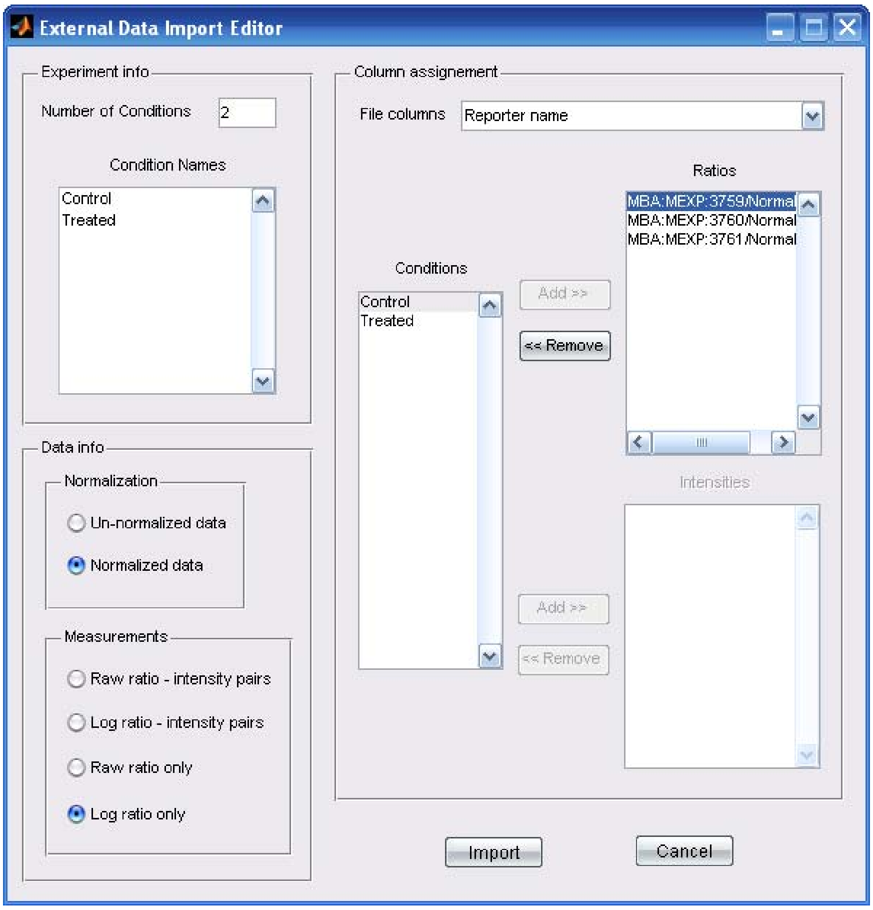

The user should see Appendix A for how this file should be structured. Briefly, each column (or pair of ratio-intensity columns) should correspond to measurements of a single array. The file should contain only one column with unique gene identifiers. As with the Data Import wizard, the user should properly fill in the number of experimental conditions of the dataset and proper condition names (the user should see 2.5.1 for information on proper condition names). After setting the above parameters, the user should provide some information on the contents of the file. The following table explains the types of processed data that can be imported to ARMADA.

**Type**	**Description**
Un-normalized data	Such data should be structured in ratio-intensity pairs. Ratio is the ratio between channel 2 (treated) and channel 1 (reference) data while intensity is an estimate of spot intensity based on the signals of the two channels (the user should see Apendix A). If the provided ratios are not log_2 transformed, the user should choose "**Raw ratio-intensity pairs**" and ratios will be log_2 transformed. If ratios are already log_2 transformed, the user should choose "**Log ratio-intensity pairs**". Un-normalized ratios alone cannot be imported.
Normalized data	Such data should be structured in ratio-intensity pairs or ratios alone. Ratio is the ratio between channel 2 (sample) and channel 1 (reference) data while intensity is an estimate of spot intensity based on the signals of the two channels (the user should see Apendix A). If the provided ratios are not log_2 transformed, the user should choose "**Raw ratio-intensity pairs**" or "**Raw ratio**" only depending on the nature of the data and ratios will be log_2 transformed. If ratios are already log_2 transformed, the user should choose

"**Log ratio-intensity pairs**" or "**Log ratio**". The user should note that some of the data exploration plots will not be available in the case of only ratio data.

The list **File columns** contains the column headers found in the data file to be imported. The user should use this list and the buttons **Add>>** and **<<Remove** to assign proper ratio-intensity pairs (or ratios only) to each of the conditions in the **Conditions** list. At any point the user can see the assigned ratios and intensities in the **Ratios** and **Intensities** lists[5]. All columns of the file should be assigned to an experimental condition and the only column that will remain in the **File columns** list should contain a unique gene identifier. After properly setting all the above parameters, the user should click **Import** for the data to be imported to ARMADA.

2.6. Exploring data – main window

2.6.1. ARMADA's main window

The image below depicts the main window of ARMADA. It consists of the history textbox, the project tree view, the array list, the analysis objects list, the image (or data) area and some shortcut buttons for main functionalities of the program. The menu bar provides access to all the platform's functions and abilities. This and the following sections describe the functionalities that can be accessed directly from the main window and present the first steps of data exploration with ARMADA.

[5] Special attention should be paid during column assignment. It is of great importance for the relevance of the subsequent analysis.

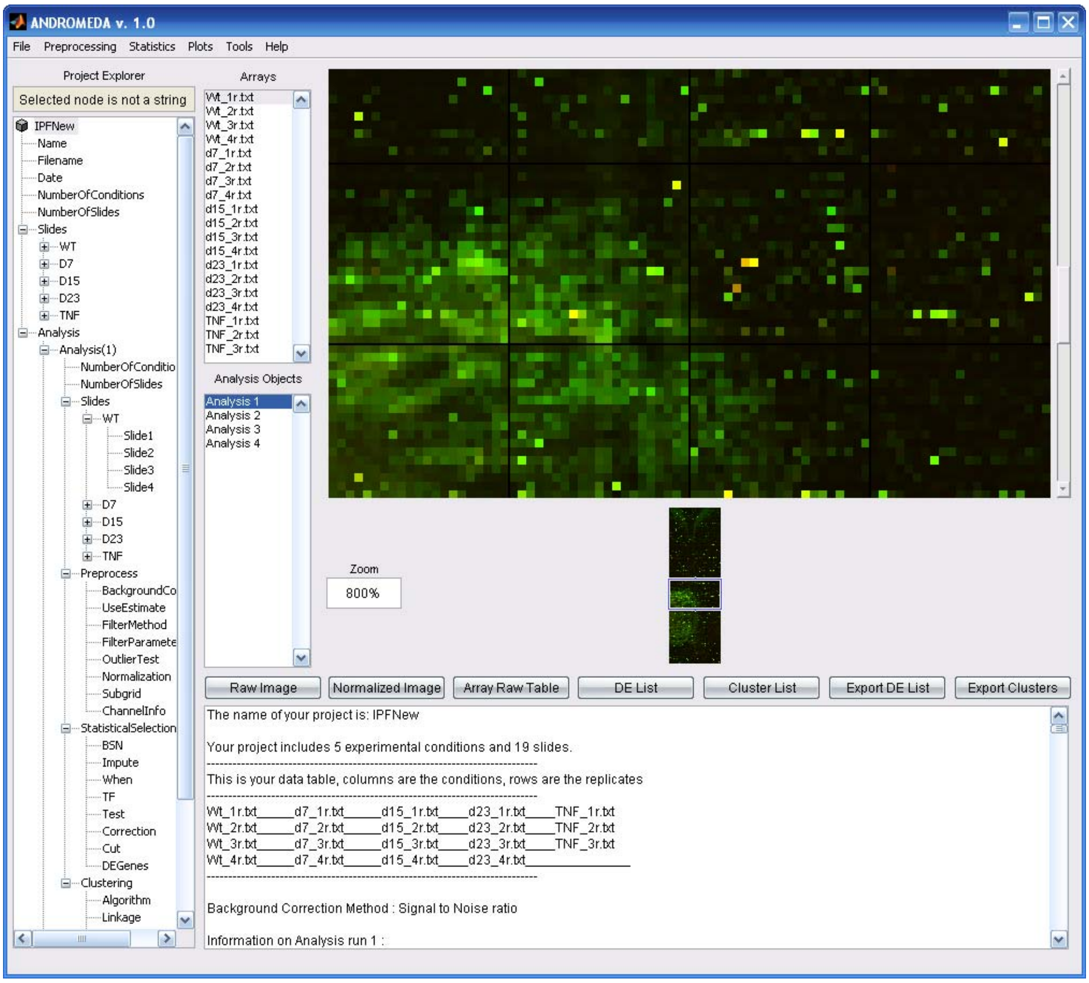

2.6.2. History textbox

The history textbox is placed at the right bottom in the main window. It contains messages produced during different analysis steps in the project. These messages could be used in the production of reports.

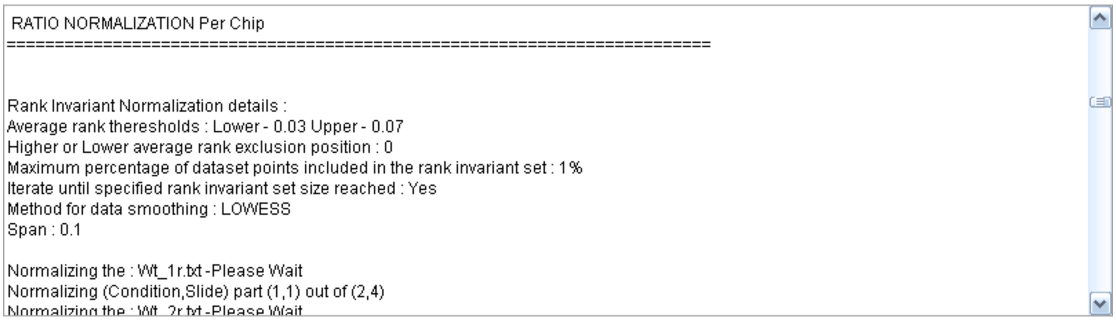

2.6.3. Tree view

The tree view is placed in the left side of the main window and displays analysis history in the form of the tree. It contains several summary data for each analysis in your project, such as the number of conditions and slides included in the analysis, which filtering or statistical methods were used etc.

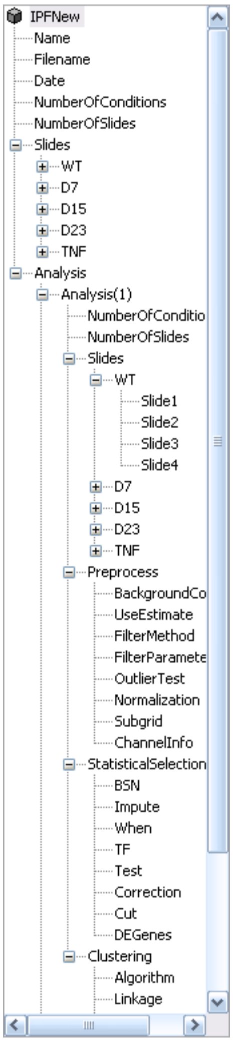

The following table presents the names of the tree branches:

**Branch name**	**Description**
Name	The name of the project.
Filename	The path and filename of the project.
Date	The date the project was created.
NumberOfConditions	When placed directly under the root of the tree, it represents the total number of experimental conditions in the project, while when placed under an "Analysis" branch it represents the number of condition for that specific analysis.
NumberOfSlides	When placed directly under the root of the tree, it represents the total number of microarrays in the project, while when placed under an "Analysis" branch it represents the number of microarrays for that specific analysis.
Slides	A sub-tree containing the experimental conditions as branch names and the names of the data files as leafs.
Analysis	Different analyses of the project (e.g. using a different set of conditions or different preprocessing or statistical selection methods.
Preprocess	Branch name for the preprocessing steps used in the project. Its children contain detail on the preprocessing methods used (the user should see "Preprocessing data").
BackgroundCorrection	The background correction method used.
UseEstimate	The main signal estimation (mean or median).

FilterMethod	The spot filtering method used.
FilterParameter	The parameters used with the spot filtering method used with the filtering method.
OutlierTest	The statistical test that was used for outlier detection among replicates of the same condition (if performed).
Normalization	The name of the within slide normalization method that was utilized (if any).
Span	The spanning neighbourhood size for LOWESS/LOESS normalization methods.
Subgrid	Whether block dependent normalization was chosen to be performed or not.
ChannelInfo	Whether the 2nd channel (sample channel) refers to Cy3 or Cy5.
StatisticalSelection	Branch name for the statistical selection steps applied in the data of project after preprocessing. Its children contain details on the steps performed.
BSN	The name of the **B**etween **S**lide **N**ormalization (BSN) that was utilized (if any).
Impute	The missing value imputation algorithm that was used to impute any missing value caused by the image processing or the filtering steps.
When	Impute missing values before or after between slide normalization.
TF	The **T**rust **F**actor filter value.
Test	The statistical test used in the statistical selection process.
Correction	The multiple testing correction procedure applied (if any).
Cut	The p-value (or FDR depending on multiple testing correction method) threshold applied in the process of statistical selection.
DEgenes	The number of **D**ifferentially **E**xpressed genes found after the application of a statistical test.
Clustering	Branch name for clustering steps applied in the data of the project after statistical selection. Its children contain details on the steps performed.
Algorithm	The clustering algorithm utilized.
Linkage	The linkage algorithm used (only in the case of hierarchical clustering).
Distance	The distance calculation metric used with the chosen clustering algorithm.
Seed	The initial cluster position (only in the case of k-means clustering).
Limit	The limitation method used to identify the number of clusters.
PValue	A p-value cutoff to filter the differentially expressed genes to be clustered after the statistical selection process.
Clusters	The number of clusters found.
SVM	Branch name for **S**upport **V**ector **M**achine classifier constructed for classification purposes after statistical selection.
Kernel	The kernel function type of the SVM classifier.
Parameters	The parameter set for the SVM classifier.

2.6.4. Arrays list

The arrays list is a list of all the files (arrays) that were imported to the project. By selecting an array from the list and right clicking inside the list, a menu with the following options appears:

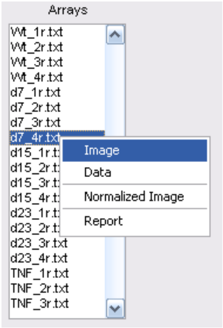

The functionalities of the submenu commands are explained in the following table:

Command name	Description
Image	Displays an image for the selected array which is reconstructed from the raw data.
Data	Displays a data table reconstructed from the columns of the selected file.
Normalized Image	Displays an image for the selected array which is reconstructed from log_2 ratios after the normalization procedure.
Report	Displays information concerning the selected array in a separate window.

2.6.5. Analysis Object List

The analysis list is a list of all the different analyses that have been created in the current project. By selecting an array from the list and right clicking inside the list, a menu with the following options appears:

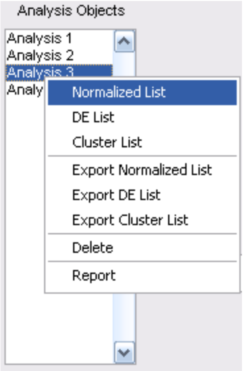

The functionalities of the submenu commands are explained in the following table:

Command name	Description
Normalized List	Displays the list of genes of the selected analysis and their expression values (data displayed customizable) after the normalization procedure.
DE List	Displays the list of **D**ifferentially **E**xpressed genes of the selected analysis and their expression values (data displayed customizable) after the statistical selection procedure.
Cluster List	Displays the list of clusters and the genes belonging to each cluster coupled by their expression values after the clustering procedure.
Export Normalized List	Exports the list of genes of the selected analysis and their expression values (data exported and export format customizable) after the normalization procedure.
Export DE List	Exports the list of **D**ifferentially **E**xpressed genes of the selected analysis and their expression values (data exported and export format customizable) after the statistical selection procedure.
Export Cluster List	Exports the list of clusters and the genes belonging to each cluster coupled by their expression values after the clustering procedure (export format customizable).
Delete	Deletes the selected analysis and all its components.
Report	Displays a brief report on analysis steps and results in a new window.

2.6.6. Raw Image

By hitting the **Raw Image** button in the main window, if array block coordinates are provided, an image is reconstructed based on these spatial data by overlaying raw signal data from both channels for the selected array from the Arrays list. If coordinates are not provided, ARMADA creates an image with grid size equal to the number of genes on the arrays multiplied by the number of slides on the project. In this way, each row of the reconstructed image presents a gene and each column the corresponding slide. Array images in the main window can also be created by right-clicking on the selected array in the Arrays list and selecting **Image** or by clicking **View → Raw Image**.

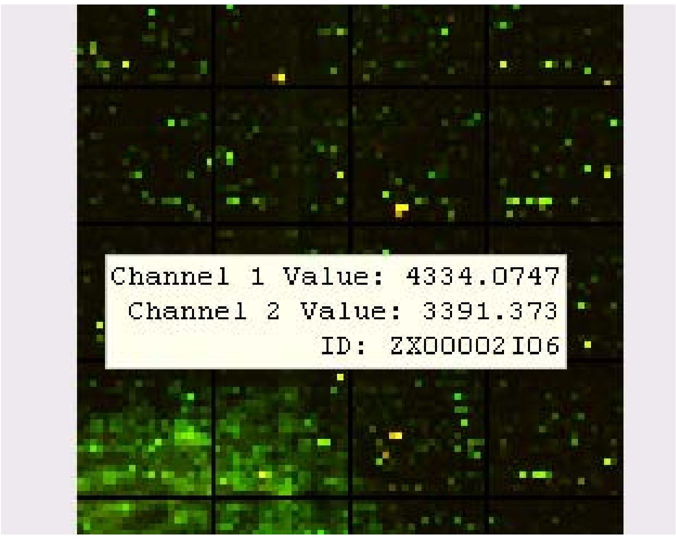

The user can click on raw images and view individual spot data. Zooming and scrolling is also possible.

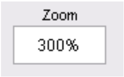

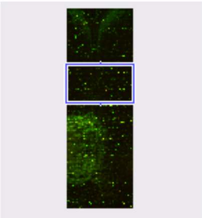

2.6.7. Normalized Image

By hitting the **Normalized Image** button in the main window, if array block coordinates are provided, an image is reconstructed based on these spatial data using normalized log_2 ratio data for the selected microarray from the Arrays list. If coordinates are not provided, ARMADA creates an image with grid size equal to the number of genes on the arrays multiplied by the number of slides on the project. In this way, each row of the reconstructed image presents a gene and each column the corresponding slide. Normalized array images in the main window can also be created by right-clicking on the selected array in the Arrays list and selecting **Normalized Image** or by clicking **View → Normalized Image**.

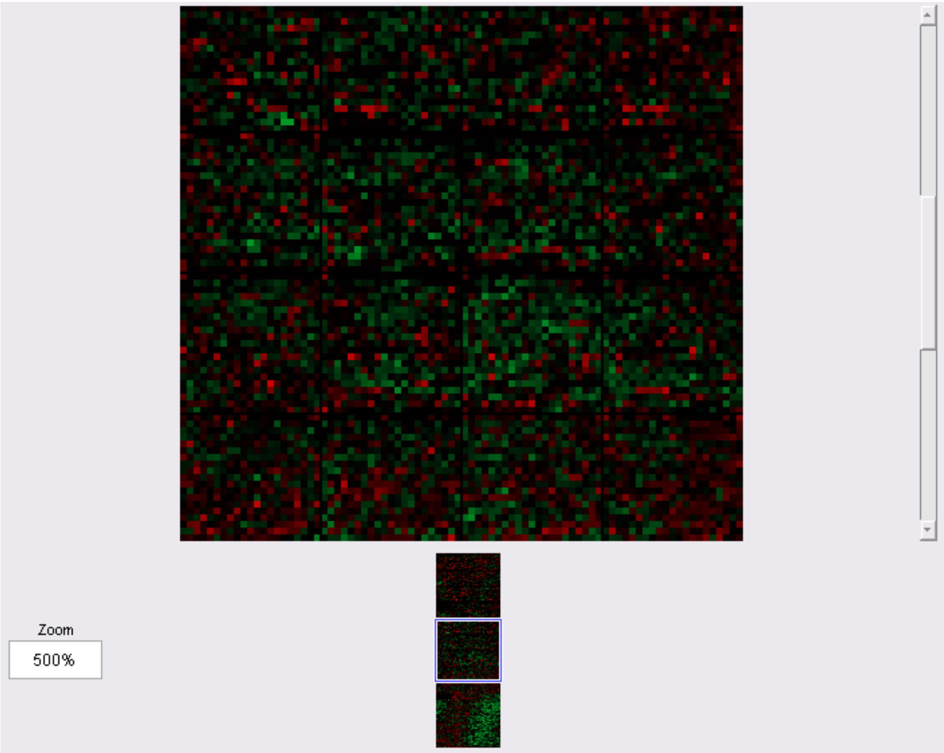

2.6.8. Array Raw Table

By clicking on the **Array Raw Table** button, ARMADA displays a spreadsheet like view which contains basic data derived directly from the input file(s) for the selected array from the Arrays list. The same data can be displayed by right-licking on a selected array from the Arrays list and then clicking on **Data**, or by clicking **View → Raw Data**.

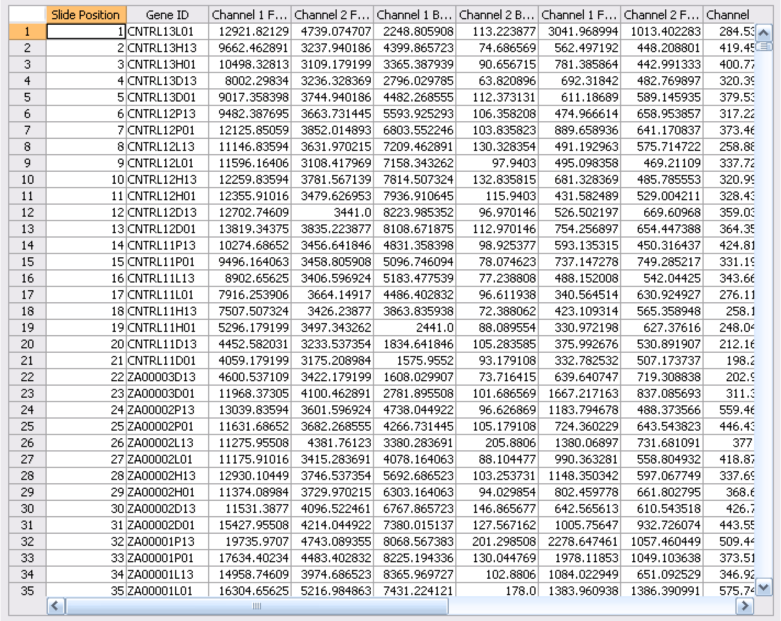

	Slide Position	Gene ID	Channel 1 F...	Channel 2 F...	Channel 1 B...	Channel 2 B...	Channel 1 F...	Channel 2 F...	Channel
1	1	CNTRL13L01	12921.82129	4739.074707	2248.805908	113.223877	3041.968994	1013.402283	284.53
2	2	CNTRL13H13	9662.462891	3237.940186	4399.865723	74.686569	562.497192	448.208801	419.45
3	3	CNTRL13H01	10498.32813	3109.179199	3365.387939	90.656715	781.385864	442.991333	400.77
4	4	CNTRL13D13	8002.29834	3236.328369	2796.029785	63.820896	692.31842	482.769897	320.39
5	5	CNTRL13D01	9017.358398	3744.940186	4482.268555	112.373131	611.18689	589.145935	379.53
6	6	CNTRL12P13	9482.387695	3663.731445	5593.925293	106.358208	474.966614	658.953857	317.22
7	7	CNTRL12P01	12125.85059	3852.014893	6803.552246	103.835823	889.658936	641.170837	373.46
8	8	CNTRL12L13	11146.83594	3631.970215	7209.462891	130.328354	491.192963	575.714722	258.88
9	9	CNTRL12L01	11596.16406	3108.417969	7158.343262	97.9403	495.098358	469.21109	337.72
10	10	CNTRL12H13	12259.83594	3781.567139	7814.507324	132.835815	681.328369	485.785553	320.99
11	11	CNTRL12H01	12355.91016	3479.626953	7936.910645	115.9403	431.582489	529.004211	328.43
12	12	CNTRL12D13	12702.74609	3441.0	8223.985352	96.970146	526.502197	669.60968	359.03
13	13	CNTRL12D01	13819.34375	3835.223877	8108.671875	112.970146	754.256897	654.447388	364.35
14	14	CNTRL11P13	10274.68652	3456.641846	4831.358398	98.925377	593.135315	450.316437	424.81
15	15	CNTRL11P01	9496.164063	3458.805908	5096.746094	78.074623	737.147278	749.285217	331.19
16	16	CNTRL11L13	8902.65625	3406.596924	5183.477539	77.238808	488.152008	542.04425	343.66
17	17	CNTRL11L01	7916.253906	3664.14917	4486.402832	96.611938	340.564514	630.924927	276.11
18	18	CNTRL11H13	7507.507324	3426.23877	3863.835938	72.388062	423.109314	565.358948	258.1
19	19	CNTRL11H01	5296.179199	3497.343262	2441.0	88.089554	330.972198	627.37616	248.04
20	20	CNTRL11D13	4452.582031	3233.537354	1834.641846	105.283585	375.992676	530.891907	212.16
21	21	CNTRL11D01	4059.179199	3175.208984	1575.9552	93.179108	332.782532	507.173737	198.2
22	22	ZA00003D13	4600.537109	3422.179199	1608.029907	73.716415	639.640747	719.308838	202.9
23	23	ZA00003D01	11968.37305	4100.462891	2781.895508	101.686569	1667.217163	837.085693	311.3
24	24	ZA00002P13	13039.83594	3601.596924	4738.044922	96.626869	1183.794678	488.373566	559.46
25	25	ZA00002P01	11631.68652	3682.268555	4266.731445	105.179108	724.360229	643.543823	446.43
26	26	ZA00002L13	11275.95508	4381.76123	3380.283691	205.8806	1380.06897	731.681091	377
27	27	ZA00002L01	11175.91016	3415.283691	4078.164063	88.104477	990.363281	558.804932	418.87
28	28	ZA00002H13	12930.10449	3746.537354	5692.686523	103.253731	1148.350342	597.067749	337.69
29	29	ZA00002H01	11374.08984	3729.970215	6303.164063	94.029854	802.459778	661.802795	368.6
30	30	ZA00002D13	11531.3877	4096.522461	6767.865723	146.865677	642.565613	610.543518	426.7
31	31	ZA00002D01	15427.95508	4214.044922	7380.015137	127.567162	1005.75647	932.726074	443.55
32	32	ZA00001P13	19735.9707	4743.089355	8068.567383	201.298508	2278.647461	1057.460449	509.44
33	33	ZA00001P01	17634.40234	4483.402832	8225.194336	130.044769	1978.11853	1049.103638	373.51
34	34	ZA00001L13	14958.74609	3974.686523	8365.969727	102.8806	1084.022949	651.092529	346.92
35	35	ZA00001L01	16304.65625	5216.984863	7431.224121	178.0	1383.960938	1386.390991	575.74

2.6.9. Normalized List

By right-clicking on and Analysis object from the Analysis Objects lists and selecting **Normalized List**, ARMADA displays a spreadsheet like view which contains normalized data for

the selected Analysis. The same data can be displayed by clicking **View → Normalized Data**. The data elements displayed are customizable (the user should see section 5).

	Slide Position	GeneID	Normalized L...	Normalized L...	Normalized L...	Normalized L...	Mean Norma...	StDev Norm...	Mean Int
1	1.0	CNTRL13L01	NaN	0.06837191...	0.95424264...	0.41627659...	0.47963038...	0.44632052...	3.83024
2	2.0	CNTRL13H13	NaN	-0.0664771...	NaN	0.66158268...	0.29755275...	0.51481605...	1.87311
3	3.0	CNTRL13H01	NaN	0.01234704...	0.29524241...	0.18339866...	0.16366270...	0.14247658...	2.06500
4	4.0	CNTRL13D13	NaN	0.04420318...	NaN	0.33104754...	0.18762536...	0.20282959...	1.81654
5	5.0	CNTRL13D01	NaN	-0.0908056...	8.76362621...	0.30477361...	0.07161478...	0.20705959...	2.06105
6	6.0	CNTRL12P13	NaN	0.05558527...	-0.0254250...	0.30627249...	0.11214425...	0.17293061...	2.07489
7	7.0	CNTRL12P01	NaN	-0.1139568...	-0.1162949...	0.17008064...	-0.0200570...	0.16466822...	2.06454
8	8.0	CNTRL12L13	NaN	-0.1066155...	0.26464372...	0.68051506...	0.27951440...	0.39377596...	2.17351
9	9.0	CNTRL12L01	NaN	-0.0087301...	0.29922796...	0.48757770...	0.25935850...	0.25054451...	2.21748
10	10.0	CNTRL12H13	NaN	2.82960262...	0.74367381...	0.68480947...	0.47625541...	0.41325365...	2.43741
11	11.0	CNTRL12H01	NaN	0.04641658...	NaN	0.33500772...	0.19071215...	0.20406475...	1.84748
12	12.0	CNTRL12D13	NaN	-0.1130260...	NaN	0.39670203...	0.14183801...	0.36043215...	1.72954
13	13.0	CNTRL12D01	NaN	0.03356146...	-0.2832726...	0.45200798...	0.06743225...	0.36880867...	2.05243
14	14.0	CNTRL11P13	NaN	-5.6955328...	NaN	0.03774172...	0.01858608...	0.02709016...	1.79304
15	15.0	CNTRL11P01	NaN	-0.1240653...	NaN	0.18947641...	0.03270551...	0.22170752...	1.73394
16	16.0	CNTRL11L13	NaN	0.04468273...	NaN	-0.1098476...	-0.0325824...	0.10926948...	1.72362
17	17.0	CNTRL11L01	NaN	-0.0985912...	NaN	0.19077192...	0.04609032...	0.20461068...	1.65058
18	18.0	CNTRL11H13	NaN	-0.1143307...	NaN	-0.0339772...	-0.0741540...	0.05681853...	1.75874
19	19.0	CNTRL11H01	NaN	-0.1426326...	NaN	0.06442995...	-0.0391013...	0.14641536...	1.68996
20	20.0	CNTRL11D13	NaN	-0.0319784...	NaN	0.35992535...	0.16397347...	0.27711780...	1.73775
21	21.0	CNTRL11D01	NaN	-0.1089483...	0.15147798...	-0.1852966...	-0.0475890...	0.17657298...	1.93694
22	22.0	ZA00003D13	NaN	0.08343491...	0.17488408...	-0.1329239...	0.04179834...	0.15807167...	2.42001
23	23.0	ZA00003D01	NaN	-0.0301441...	0.46897245...	-0.1848757...	0.08465083...	0.34170571...	2.68700
24	24.0	ZA00002P13	NaN	-0.0304733...	-0.1228075...	-0.2076341...	-0.1203050...	0.08860694...	2.33588
25	25.0	ZA00002P01	NaN	-0.0907102...	-0.3184901...	-0.3761002...	-0.2617669...	0.15091389...	2.19983
26	26.0	ZA00002L13	NaN	-0.2104689...	-0.3329277...	-0.2656781...	-0.2696916...	0.06132798...	2.23287
27	27.0	ZA00002L01	NaN	-0.0562167...	-0.3225438...	-0.1730056...	-0.1839220...	0.13349870...	2.31177
28	28.0	ZA00002H13	NaN	-0.2438186...	-0.4405987...	0.00277324...	-0.2272147...	0.22215184...	2.22886
29	29.0	ZA00002H01	NaN	-0.0503975...	0.24817430...	-0.0769048...	0.04029063...	0.18051973...	2.08074
30	30.0	ZA00002D13	NaN	0.08647994...	-0.1085416...	0.06104292...	0.01299374...	0.10601837...	2.08682
31	31.0	ZA00002D01	NaN	-0.0569397...	0.20930017...	-0.2501061...	-0.0325818...	0.23066969...	2.50829
32	32.0	ZA00001P13	NaN	0.33851902...	0.01459660...	-0.0799106...	0.09106833...	0.21944655...	2.67414
33	33.0	ZA00001P01	NaN	0.05738228...	-0.3243329...	-0.4209556...	-0.2293021...	0.25293269...	2.50027
34	34.0	ZA00001L13	NaN	-0.2555741...	NaN	-0.0430242...	-0.1492991...	0.15029542...	1.75940
35	35.0	ZA00001L01	NaN	0.11429486...	-0.4972774...	-0.5893595...	-0.3241140...	0.38245465...	2.51912

2.6.10. Differentially Expressed genes List

By clicking on the **DE List** (**D**ifferentially **E**xpressed) button, ARMADA displays a spreadsheet like view which contains data for the differentially expressed genes derived from a statistical selection process. The data displayed correspond to the selected Analysis from the Analysis Objects list. The same data can be displayed by right-licking on the selected Analysis from the Analysis Objects list and then clicking on **DE List**, or by clicking **View → DE Genes List**.

	Slide Positions	GeneID	p-value	Normalized L...	Normalized L...	Normalized L...	Normalized L...	Mean Norma...	StDev Nor
1	24	ZA00002P13	0.01553916...	-0.5140957...	-0.1716506...	-0.2904878...	-0.5140957...	-0.3725825...	0.092589
2	87	ZX00003P01	0.04524206...	-2.6015188...	-0.4812512...	0.33539716...	-1.2357528...	-0.9957814...	0.470671
3	171	ZX00014H01	0.00911155...	-1.8539887...	-3.1265262...	-1.4933382...	-1.4933382...	-1.9917978...	0.113463
4	193	ZX00016P01	0.02539326...	-0.2701940...	-0.6631876...	-1.9361905...	-1.9361905...	-1.2014407...	0.282574
5	213	ZX00019L01	0.03234523...	0.50308959...	0.44821289...	-0.1144763...	-2.5252714...	-0.4221113...	0.574967
6	241	ZX00021H01	0.01359739...	-1.3615470...	-2.4132194...	0.62230802...	-1.3615470...	-1.1285013...	0.616019
7	253	ZX00025D01	0.03684518...	-1.7264890...	-0.0338474...	2.49256778...	-1.8278883...	-0.2739142...	2.574121
8	281	ZX00026P01	0.00720329...	-2.0133258...	-0.3993551...	-0.0751294...	-2.0133258...	-1.1252840...	0.358477
9	282	ZX00026L13	0.01843009...	-0.6718848...	0.21913906...	1.32108068...	-0.6718848...	0.04911252...	0.883003
10	314	ZX00027P13	0.00939236...	-2.6423339...	-0.5194353...	-0.0915380...	-2.6423339...	-1.4739103...	0.392369
11	327	ZX00031L01	0.04891699...	-0.5319141...	2.05676584...	-1.0590764...	-1.3846902...	-0.2297287...	1.825726
12	336	ZX00030H13	0.03170576...	-1.5854407...	0.50989167...	-1.8514422...	-1.5854407...	-1.1281080...	0.555341
13	341	ZX00035D01	0.04204787...	-2.6647512...	-1.5404782...	3.40490121...	-1.7912153...	-0.6478859...	5.164849
14	360	ZX00048P13	0.04076538...	0.24750770...	0.19940122...	0.07864490...	-1.7120992...	-0.2966363...	0.416273
15	361	ZX00048P01	0.02360243...	-0.1867841...	-0.6771326...	0.21418038...	-1.3975639...	-0.5118250...	0.335127
16	379	KG00002P01	0.01715951...	0.09877379...	-0.4012926...	0.78989417...	-0.8620497...	-0.0936686...	0.514724
17	380	KG00002L13	0.04528013...	-1.4906205...	-0.9619028...	0.96728637...	-1.5327807...	-0.7545044...	0.778898
18	400	KG00006H13	0.01493967...	0.58975227...	-1.2827396...	-0.5374328...	-0.5374328...	-0.4419632...	0.472847
19	428	CNTRL14D13	0.00514420...	0.83270608...	-0.7396525...	-0.3197873...	-0.3197873...	-0.1366302...	0.532248
20	433	NP00001L01	0.02187174...	-1.2617788...	-0.3203742...	0.39518048...	-1.2617788...	-0.6121878...	0.425459
21	468	ZA00002K01	0.02588446...	-0.5315799...	-1.0297541...	-0.9165801...	-0.9165801...	-0.8486235...	0.089680
22	495	ZA00004G13	0.01738170...	-0.2021967...	-0.9112755...	-0.6495440...	-0.6495440...	-0.6031401...	0.142506
23	563	ZX00004K13	0.04297893...	0.51401375...	0.77831881...	1.51341065...	-1.0530312...	0.43817800...	0.977392
24	615	ZX00013O13	0.04985758...	0.60455542...	-1.4154252...	0.24515612...	-2.2586067...	-0.7060801...	0.631280
25	635	ZX00016K13	0.01345936...	-0.4593538...	-0.5406426...	-1.1432297...	-1.1432297...	-0.8216140...	0.147915
26	801	ZX00048O13	0.02960167...	-1.4555292...	-0.6627394...	-0.1378365...	-1.4555292...	-0.9279086...	0.260114
27	803	ZX00048K13	0.02491239...	-0.4236478...	-0.7661551...	4.52981593...	-2.0038374...	0.33404385...	11.28804
28	805	ZX00048G13	0.03490098...	0.25709182...	-0.8210970...	3.79222398...	-0.1646631...	0.76588891...	6.489850
29	820	KG00002O01	0.01904997...	-1.3648943...	-1.6384931...	-1.6181425...	-1.3648943...	-1.4966061...	0.037452
30	873	NP00001K13	0.03787566...	0.90568680...	0.28337285...	-0.3429297...	-0.3429297...	0.12580005...	0.512566
31	883	CNTRL13J01	4.51284386...	0.02343750...	-0.5773808...	0.23374249...	0.15315455...	-0.0417615...	0.225362
32	903	CNTRL11B01	0.00737537...	-0.4032208...	-0.4731331...	1.47334827...	-0.4032208...	0.04844334...	1.016345
33	907	ZA00002N01	0.00740561...	0.61085261...	0.84104774...	0.56003844...	-0.1666848...	0.46131349...	0.379601
34	920	ZA00001B13	0.00974793...	-0.3397404...	-0.7094245...	-0.0158473...	-0.3397404...	-0.3511881...	0.154229
35	940	ZA00003N13	0.00247012...	-0.6567064...	-0.5954637...	-0.4331337...	-0.6567064...	-0.5855025...	0.050279

2.6.11. Cluster List

By clicking on the **Cluster List** button, ARMADA displays a spreadsheet like view which contains data concerning gene cluster memberships derived after clustering procedures. The data displayed correspond to the selected Analysis from the Analysis Objects list. The same data can be displayed by right-licking on the selected Analysis from the Analysis Objects list and then clicking on **Cluster List**, or by clicking **View → Gene Clusters List**.

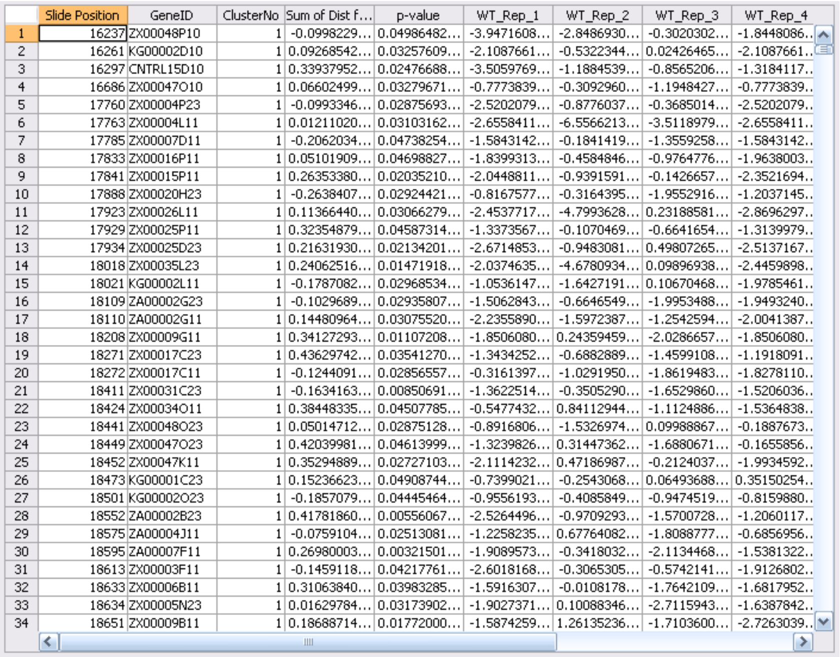

	Slide Position	GeneID	ClusterNo	Sum of Dist f...	p-value	WT_Rep_1	WT_Rep_2	WT_Rep_3	WT_Rep_4
1	16237	ZX00048P10	1	-0.0998229...	0.04986482...	-3.9471608...	-2.8486930...	-0.3020302...	-1.8448086..
2	16261	KG00002D10	1	0.09268542...	0.03257609...	-2.1087661...	-0.5322344...	0.02426465...	-2.1087661..
3	16297	CNTRL15D10	1	0.33937952...	0.02476688...	-3.5059769...	-1.1884539...	-0.8565206...	-1.3184117..
4	16686	ZX00047O10	1	0.06602499...	0.03279671...	-0.7773839...	-0.3092960...	-1.1948427...	-0.7773839..
5	17760	ZX00004P23	1	-0.0993346...	0.02875693...	-2.5202079...	-0.8776037...	-0.3685014...	-2.5202079..
6	17763	ZX00004L11	1	0.01211020...	0.03103162...	-2.6558411...	-6.5566213...	-3.5118979...	-2.6558411..
7	17785	ZX00007D11	1	-0.2062034...	0.04738254...	-1.5843142...	-0.1841419...	-1.3559258...	-1.5843142..
8	17833	ZX00016P11	1	0.05101909...	0.04698827...	-1.8399313...	-0.4584846...	-0.9764776...	-1.9638003..
9	17841	ZX00015P11	1	0.26353380...	0.02035210...	-2.0448811...	-0.9391591...	-0.1426657...	-2.3521694..
10	17888	ZX00020H23	1	-0.2638407...	0.02924421...	-0.8167577...	-0.3164395...	-1.9552916...	-1.2037145..
11	17923	ZX00026L11	1	0.11366440...	0.03066279...	-2.4537717...	-4.7993628...	0.23188581...	-2.8696297..
12	17929	ZX00025P11	1	0.32354879...	0.04587314...	-1.3373567...	-0.1070469...	-0.6641654...	-1.3139979..
13	17934	ZX00025D23	1	0.21631930...	0.02134201...	-2.6714853...	-0.9483081...	0.49807265...	-2.5137167..
14	18018	ZX00035L23	1	0.24062516...	0.01471918...	-2.0374635...	-4.6780934...	0.09896938...	-2.4459898..
15	18021	KG00002L11	1	-0.1787082...	0.02968534...	-1.0536147...	-1.6427191...	0.10670468...	-1.9785461..
16	18109	ZA00002G23	1	-0.1029689...	0.02935807...	-1.5062843...	-0.6646549...	-1.9953488...	-1.9493240..
17	18110	ZA00002G11	1	0.14480964...	0.03075520...	-2.2355890...	-1.5972387...	-1.2542594...	-2.0041387..
18	18208	ZX00009G11	1	0.34127293...	0.01107208...	-1.8506080...	0.24359459...	-2.0286657...	-1.8506080..
19	18271	ZX00017C23	1	0.43629742...	0.03541270...	-1.3434252...	-0.6882889...	-1.4599108...	-1.1918091..
20	18272	ZX00017C11	1	-0.1244091...	0.02856557...	-0.3161397...	-1.0291950...	-1.8619483...	-1.8278110..
21	18411	ZX00031C23	1	-0.1634163...	0.00850691...	-1.3622514...	-0.3505290...	-1.6529860...	-1.5206036..
22	18424	ZX00034O11	1	0.38448335...	0.04507785...	-0.5477432...	0.84112944...	-1.1124886...	-1.5364838..
23	18441	ZX00048O23	1	0.05014712...	0.02875128...	-0.8916806...	-1.5326974...	0.09988867...	-0.1887673..
24	18449	ZX00047O23	1	0.42039981...	0.04613999...	-1.3239826...	0.31447362...	-1.6880671...	-0.1655856..
25	18452	ZX00047K11	1	0.35294889...	0.02727103...	-2.1114232...	0.47186987...	-0.2124037...	-1.9934592..
26	18473	KG00001C23	1	0.15236623...	0.04908744...	-0.7399021...	-0.2543068...	0.06493688...	0.35150254..
27	18501	KG00002O23	1	-0.1857079...	0.04445464...	-0.9556193...	-0.4085849...	-0.9474519...	-0.8159880..
28	18552	ZA00002B23	1	0.41781860...	0.00556067...	-2.5264496...	-0.9709293...	-1.5700728...	-1.2060117..
29	18575	ZA00004J11	1	-0.0759104...	0.02513081...	-1.2258235...	0.67764082...	-1.8088777...	-0.6856956..
30	18595	ZA00007F11	1	0.26980003...	0.00321501...	-1.9089573...	-0.3418032...	-2.1134468...	-1.5381322..
31	18613	ZX00003F11	1	-0.1459118...	0.04217761...	-2.6018168...	-0.3065305...	-0.5742141...	-1.9126802..
32	18633	ZX00006B11	1	0.31063840...	0.03983285...	-1.5916307...	-0.0108178...	-1.7642109...	-1.6817952..
33	18634	ZX00005N23	1	0.01629784...	0.03173902...	-1.9027371...	0.10088346...	-2.7115943...	-1.6387842..
34	18651	ZX00009B11	1	0.18688714...	0.01772000...	-1.5874259...	1.26135236...	-1.7103600...	-2.7263039..

2.6.12. Reports

There are two kinds of brief reports that can be displayed from ARMADA in separate windows: array reports and analysis reports. Array reports can be obtained by right-clicking on an array in the Arrays list and then selecting **Report** or by clicking **View → Array Report** while analysis reports can be obtained by right-clicking on an analysis object in the Analysis Object list and then selecting **Report** or by clicking **View → Analysis Report**.

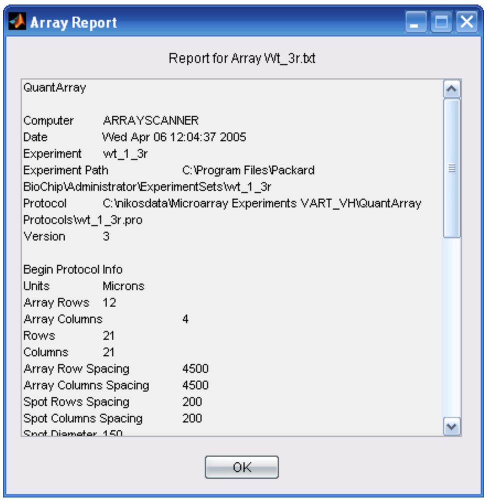

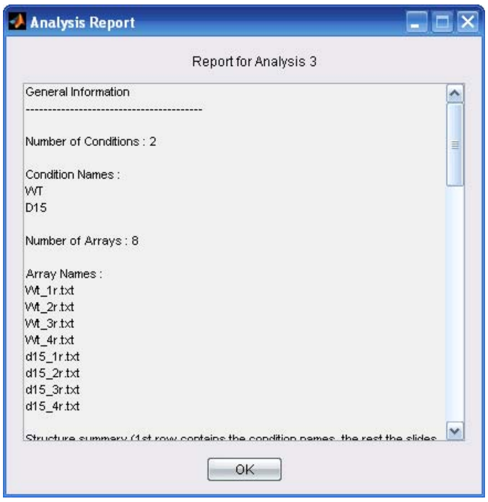

These reports display very briefly certain information on the array files imported (if header information is available when importing directly from the supported image analysis software, this information is displayed too) or the analysis steps followed and results obtained. By right-clicking inside the report window, the user can export the displayed information in a text file.

2.6.13. Deleting analysis objects

In order to delete an analysis object, the user should select the analysis to be deleted from the Analysis Object list, right-click on the selected analysis and click **Delete**. The selected analysis will be deleted and the number of the following analysis in the list will be decreased in order to keep a continuous analysis object numbering. For example, if there are 5 analysis object named 'Analysis 1', 'Analysis 2',…, 'Analysis 5', if the user deletes 'Analysis 4', 'Analysis 5' will be renamed to 'Analysis 4'.

3. Preprocessing Data

The first steps on analyzing data derived from microarray experiments consist from several preprocessing steps which include quality control, data filtering and normalization to assure the quality of the data that will be used to extract results and compensate for systematic error measurements among different arrays. The next sections describe the filtering and normalization methods implemented in ARMADA and explain the program interfaces. The user should also note that data preprocessing will not be available if the data import step is not completed properly.

3.1. Selecting subjects of experimental conditions

In order to create an analysis object, the user should first choose a subset of experimental conditions and replicates from the total set of the imported arrays. If this step is skipped for the first analysis, ARMADA will apply the chosen preprocessing procedures to the whole dataset. The application of the same preprocessing steps on the whole dataset is recommended when the user wishes to preprocess the data in a same manner (e.g. with the same normalization method) and then be able to choose different sets of experimental conditions to perform different statistical tests without re-performing the sometimes time consuming step of normalization.

The user is able to select different sets of experimental conditions and replicates and create different analysis objects by clicking **Preprocessing → Select Conditions**. The following window appears:

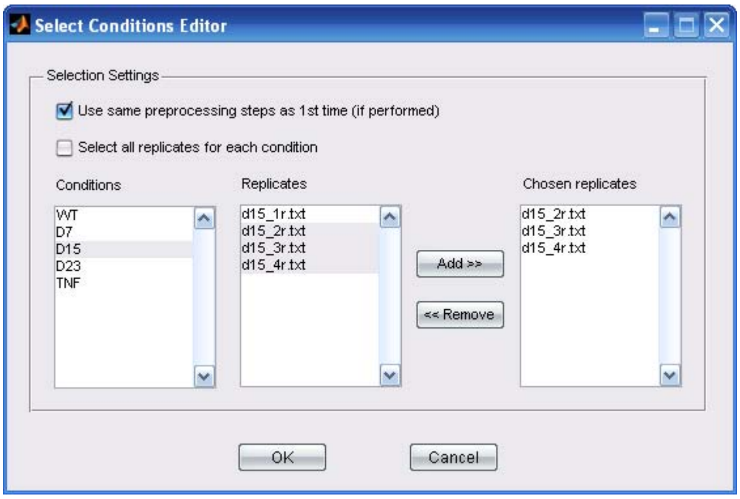

The above interface helps the user to create a new analysis object by defining which experimental conditions and which arrays from each experimental condition should be included in the analysis object. This feature is helpful for example when experiment quality control or filtering has shown that an array from one of the conditions is of poor quality and maybe should not be used in further analysis but should be imported or remain to ARMADA to perform data exploration on this array (e.g. create array images and compare the signal with the background noise).

If the user has chosen to perform the preprocessing procedures (up to normalization) for the whole dataset (without selecting any subset of experimental conditions at the beginning), the checkbox **Use same preprocessing steps as 1st time (if performed)** will be active and the user may check it in order to define a new analysis object using part of the preprocessed whole dataset. If this option remains unchecked, a new clean analysis object will be created where the user can perform several preprocessing steps from the beginning. It should be noted that keeping the same preprocessing steps does not mean that another set of preprocessing steps cannot be applied to the selected set of experimental conditions. Also, if the user has selected a subset of experimental conditions from the beginning (e.g. Analysis 1 consists of 'WT' and 'D7' in the above example), then the option **Use same preprocessing steps as 1st time (if performed)** will not be available and the user should preprocess the data from the beginning for each analysis object created.

If there is no reason for an array to be excluded from a condition subset, the user can check the **Select all replicates for each condition** option to select directly from the list 'Conditions' without having also to select individual arrays. Finally, the **Add>>** and **<<Remove** buttons add or remove arrays in the selected experimental condition in the analysis object to be created. When finished with condition and array selection the user should click **OK** and a new analysis object will be displayed in the Analysis Object list.

3.2. Background Correction

The first step in data preprocessing with ARMADA is the definition of a background correction method to correct for background image contamination caused by several factors such as artifacts on the array surface, scratches or non-specific hybridization. To choose the background correction method, the user should click **Preprocessing → Background Correction** and the following window will appear:

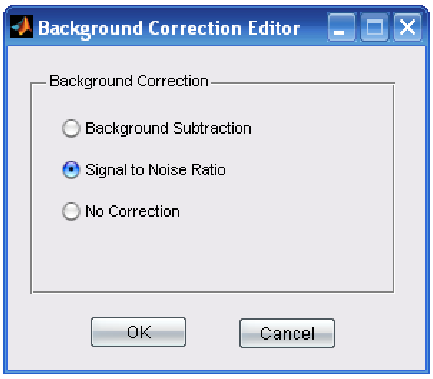

The program uses one of three possible methods (**Background Subtraction**, **Signal to noise Ratio** or **No correction**), to correct for each spot background contamination and calculate the pure signal value for each gene in each slide replicate for all conditions. Each background correction method implemented is summarized in the table below (notation: $\bar{S}$ is the signal mean/median, $\bar{B}$ is the background mean/median and $\tilde{S}$ is the net signal estimation for each spot):

Method	Description
Background Subtraction	In this case the net signal for each spot is $\tilde{S} = \bar{S} - \bar{B}$ and the log ratio between channels is $R = log_2\left(\frac{\bar{S}_{Cy5} - \bar{B}_{Cy5}}{\bar{S}_{Cy3} - \bar{B}_{Cy3}}\right) = log_2\left(\frac{\tilde{S}_{Cy5}}{\tilde{S}_{Cy3}}\right)$.
Signal to Noise Ratio	In this case the net signal for each spot is $\tilde{S} = \bar{S}/\bar{B}$ and the log ratio between channels is $R = log_2\left(\frac{\bar{S}_{Cy5}/\bar{B}_{Cy5}}{\bar{S}_{Cy3}/\bar{B}_{Cy3}}\right) = log_2\left(\frac{\tilde{S}_{Cy5}}{\tilde{S}_{Cy3}}\right)$.
No Correction	In this case the net signal for each spot is $\tilde{S} = \bar{S}$ and the log ratio between channels is $R = log_2\left(\frac{\tilde{S}_{Cy5}}{\tilde{S}_{Cy3}}\right)$.

It should be noted that the case of **Signal to Noise Ratio** takes into consideration the signal-to-noise content of a signal, an established notion in systems theory and image processing (1), thus coming in line with the perception of the experimentalist about the quality of a signal, taking into account its interest in the strength of the signal compared to noise. The following example illustrates the strength of using the signal to noise ratio for the net signal estimation compared to background subtraction which is the most common method used for background correction.

Let $\bar{S}$ denote the signal mean, $\bar{B}$ the background noise mean and $\tilde{S}$ the final signal estimation for a spot on the microarray. Let also *i*, *j* denote two arbitrary genes on the microarray and let $\bar{S}_i$=200, $\bar{B}_i$=100, $\bar{S}_j$=1100 and $\bar{B}_j$=1000. Then the net signal estimation with each of the two methods would be:

Background Subtraction	$\tilde{S}_i = \bar{S}_i - \bar{B}_i = 200 - 100 = 100$<br>$\tilde{S}_j = \bar{S}_j - \bar{B}_j = 1100 - 1000 = 100$
Signal to Noise Ratio	$\tilde{S}_i = \bar{S}_i / \bar{B}_i = 200/100 = 2$<br>$\tilde{S}_j = \bar{S}_j / \bar{B}_j = 1100/1000 = 1.1$

It can be seen that when using background subtraction, signals of different intensity range and thus with variation of different order are assigned similar corrected values, a fact that could lead to misinterpretation of subsequent analysis. On the other hand, signal-to-noise ratio provides a more rational scale of measurement and it can be seen that when signal distribution is too close to background distribution the signal-to-noise ratio is close to 1 and can be filtered easily and thus ensure constant level of comparison across all intensities range.

After selecting the desired background correction method, the user should click **OK**. If the user skips the background correction step, ARMADA sets it automatically to **No Correction**.

3.3. Spot quality filtering

After setting the background correction method, the next step in the analysis consists of spot quality filtering to exclude spots with high background contamination. By clicking **Preprocessing** → **Filtering** the following window will appear:

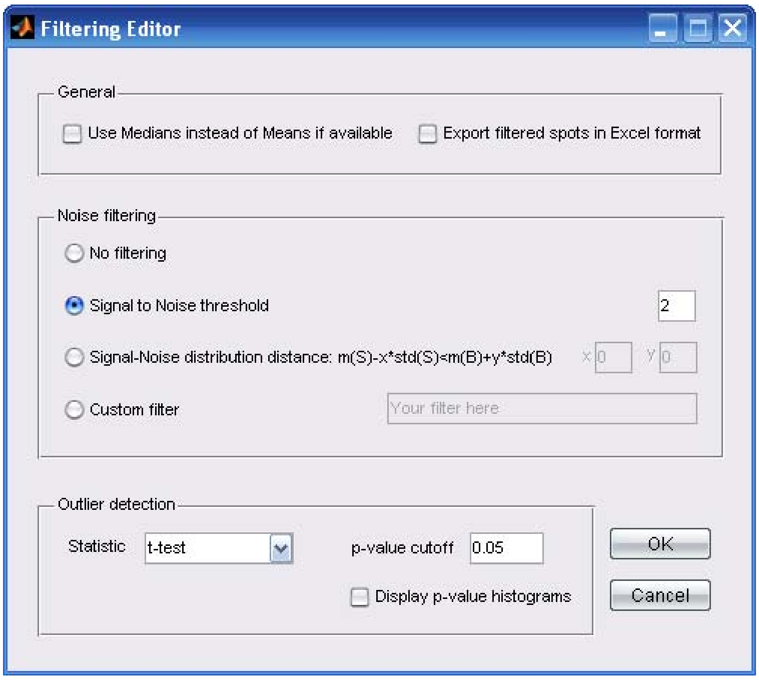

ARMADA uses by default the signal and background means to estimate the net signal that will be used to calculate expression for each spot. In the **General** panel, the user can select to use the signal and background medians instead of the means (if available) or to export the genes sensitive to the filtering processes in Excel format.

In the **Noise filtering** and **Outlier detection** panel, the user is able to select the gene filtering methods and change the default thresholds if desired. ARMADA divides spot quality filtering in 2 parts: i) spot filtering based on background noise and ii) spot filtering based on measurement reproducibility among replicates (optional). In the first step, spots marked as poor manually or by the image analysis software are excluded for every replicate and noise sensitive genes are further isolated for each slide of each condition based on one of the available filters applied to both channels. At this point, it should be noted that if data are imported directly from the supported image analysis programs, ARMADA recognizes automatically each program's spot flags and treats the flagged spots appropriately. On the other hand, if data are imported from other sources (tab delimited files, public repositories) and they contain spot flags, the user is responsible for transforming the flags column into ARMADA recognizable flags (the user should see Appendix A for file formats) or let ARMADA decide on each spot's quality by one of the supported filters. The available filters (**No filtering**, **Signal to Noise threshold**, **Signal-Noise distribution distance: m(S)-x*std(S)<m(B)+y*std(B)**, **Custom filter**) are described in the table below:

Filter	Description
No filtering	Data are filtered based only to the automatic flagging of image analysis programs or by the flags provided during importing (if they are valid flags). No other filters are applied.
Signal to Noise threshold	This filter is based on the formula $\left(\bar{S}/\bar{B}\right) < T$ where T is the threshold below which noisy spots are filtered out from each array.
Signal-Noise distribution distance: m(S)-x*std(S)<m(B)+y*std(B)	This filter is based on the distance between the signal and background distributions: a spot is robust against this filter if its signal and noise distributions abstain from each other a distance which is determined by the respective standard deviations. Sensitive spots are determined by the inequality $\bar{S} - x\sigma_S < \bar{B} + y\sigma_B$, where x and y are user-defined parameters.
Custom filter	In this case the user can create his own custom filter using any of the operators +, −, *, /, <, >, =, ~=, & (logical AND) or | (logical OR), any positive real number and any of the expressions below:<br>SigMean — Signal Mean<br>BackMean — Background Mean<br>SigMedian — Signal Median (for ImaGene, GenePix or other, if medians information present)<br>BackMedian — Background Median (for ImaGene, GenePix or other, if medians information present)<br>SigStd — Signal Standard Deviation<br>BackStd — Background Standard Deviation

As an example of the custom filter case, the following filtering expressions are valid and are applied for each microarray in the selected set of conditions on both channels:

SigMean < 2*SigStd

SigMedian – BackMedian < 500

SigMean/BackMean < 3 & SigMean < 1000

It should be noted that spot filters are applied to both channels and the union of poor spots from both channels are considered to be poor spots and excluded from further analysis.

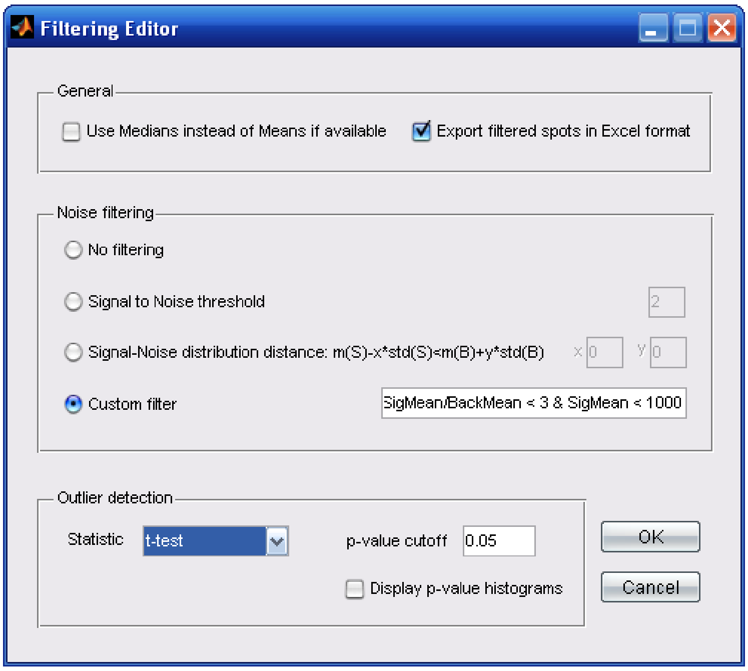

In the second filtering step which is optional (spot filtering based on measurement reproducibility among replicates for each experimental condition), the user is able to select between a t-test (parametric) or Wilcoxon (non-parametric) test that will verify that for each spot, the ratio measurements of all condition replicates derive from a normal (or a continuous symmetrical) distribution with mean (median) equal to the average ratio for this spot among all replicates. This test tracks and excludes outliers among the replicate slides of an experimental condition. If the user does not wish to perform this test, the choice of **Statistic** in the **Outlier detection** panel should be set to **None**, else, the user should select the desired test to be performed, set a statistical threshold cutoff (p-value, usually a cutoff of 0.05 is not considered neither very strict nor very loose) in the field **p-value cutoff** and choose whether or not to display histograms of p-values that depict the p-value range frequencies for all genes of an experimental condition.

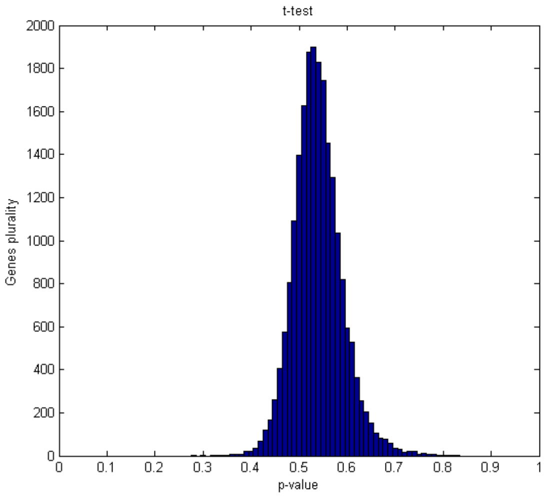

After making the necessary selections (or leaving the default settings) the user should click **OK** to perform the selected operations. At this point, ARMADA filters the selected data based on the

intensities of both channels and calculates the $\log_2$ ratio between channels as well as the intensity values (2) for the genes that passed the filtering procedures. If the option **Export filtered spots in Excel format** is checked, the following window will appear, prompting the user to select which types of filtered spots should be exported:

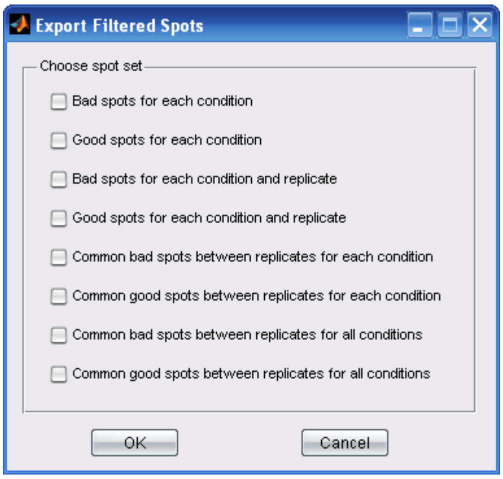

The following table describes what spots will be exported by checking each of the check boxes:

**Option**	**Description**
Bad spots for each condition	The union (all filtered spots from all replicates) of filtered spots for each experimental condition.
Good spots for each condition	The union (all remaining spots from all replicates) of remaining spots for each experimental condition.
Bad spots for each condition and replicate	Filtered spots for each experimental condition and each individual replicate.
Good spots for each condition and replicate	Remaining spots for each experimental condition and each individual replicate.
Common bad spots between replicates for each condition	The conditional intersection (filtered spots that are common between replicates) of filtered spots for each experimental condition.
Common good spots between replicates for each condition	The conditional intersection (remaining spots that are common between replicates) of remaining spots for each experimental condition.
Common bad spots between replicates for all conditions	The total intersection (filtered spots that are common between conditions) of filtered spots among all experimental conditions.
Common good spots between replicates for all conditions	The total intersection (remaining spots that are common between conditions) of remaining spots among all experimental conditions.

For example, checking **Bad spots for each condition and replicate** will return an Excel file (the user chooses the name and store location of the file) containing the filtered spots (named with their GeneID) for each array replicate under each condition in separate columns, while checking **Common bad spots between replicates for all conditions** will return an Excel file containing the spots that were commonly filtered from all conditions and replicates.

Array spots which were found to be sensitive to any of the procedures described in this section are marked as non-informative poor quality spots and excluded from the dataset to be subsequently normalized in order to alleviate the normalization procedure from the impact of systematic measurement errors. It should also be noted that if the filtering part is skipped, ARMADA assumes takes as default the **No filtering** option.

3.4. Normalization

The analysis part that follows the poor quality spot filtering in ARMADA workflow is the data normalization for each slide to compensate for systematic measurement errors. At this point it should be noted that normalization is performed on each microarray slide separately using only genes that passed the filtering tests for each slide. In order to select the normalization method and set various parameters, the user should click **Preprocessing** → **Normalization** and the following window will appear:

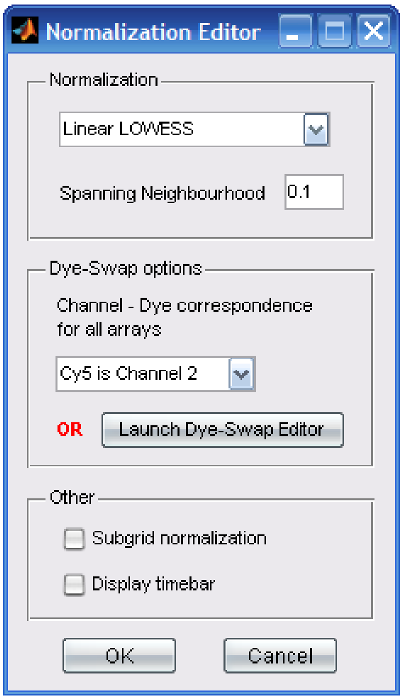

In the **Normalization** panel, the user should select among one of the currently supported normalization methods. In the case of LOWESS/LOESS methods (3) the field **Spanning Neighbourhood** should also be completed (or left with its default value). The span value modifies the running window size (proportion of neighbouring points to the currently processed point) for the smoothing function. If the span value is less than 1, the window size is taken to be a fraction of the number of points in the data. If span value is greater than 1, the running window contains as many data points as the value given. The table below describes briefly the currently supported normalization methods. The notation used is *R* for 'Red' or 'Cy5' or 'Channel 2', *G* for 'Green' or 'Cy3' or 'Channel 1' and *i* denotes gene *i* on the array under normalization. The function notation N(·) denotes normalized values.

**Method**	**Brief description**
Global Mean	Global Mean normalization normalizes data on each microarray slide by calculating the mean expression of all genes present on the array and subtracting this value from each individual gene. $N\left(log_2\left(\frac{R_i}{G_i}\right)\right) \rightarrow log_2\left(\frac{R_i}{G_i}\right) - M$ $M = mean\left(log_2\left(\frac{R_i}{G_i}\right),\ i = 1...\#\,genes\right)$
Global Median	Global Median normalization normalizes data on each microarray slide by calculating the median expression of all genes present on the array and subtracting this value from each individual gene. $N\left(log_2\left(\frac{R_i}{G_i}\right)\right) \rightarrow log_2\left(\frac{R_i}{G_i}\right) - M$ $M = median\left(log_2\left(\frac{R_i}{G_i}\right),\ i = 1...\#\,genes\right)$
LOWESS (linear fit)	LOWESS normalization normalizes data on each microarray slide by local regression of log_2 ratio against intensity using weighted linear least squares and a 1st degree polynomial model. This model is used to calculate normalized expression values for each gene. $N\left(log_2\left(\frac{R_i}{G_i}\right)\right) \rightarrow log_2\left(\frac{R_i}{G_i}\right) - f\left(log_2\left(\frac{R_i}{G_i}\right)\right)$ $f\left(log_2\left(\frac{R_i}{G_i}\right)\right) = LOWESS\left(log_2\sqrt{R_i \cdot G_i}, log_2\left(\frac{R_i}{G_i}\right)\right)$
Robust LOWESS (linear fit)	Robust LOWESS normalization normalizes data on each microarray slide by local regression of log_2 ratio against intensity using weighted linear least squares and a 1st degree polynomial model. The robust version of LOWESS performs additional fitting iterations and assigns lower weight to outliers in the regression. The method assigns zero weight to data outside six mean absolute deviations. This model is used to calculate normalized expression values for each gene. Robust LOWESS needs more time to complete than simple LOWESS but produces results more robust against possible outliers. $N\left(log_2\left(\frac{R_i}{G_i}\right)\right) \rightarrow log_2\left(\frac{R_i}{G_i}\right) - f\left(log_2\left(\frac{R_i}{G_i}\right)\right)$ $f\left(log_2\left(\frac{R_i}{G_i}\right)\right) = RobustLOWESS\left(log_2\sqrt{R_i \cdot G_i}, log_2\left(\frac{R_i}{G_i}\right)\right)$
LOESS (quadratic fit)	LOESS normalization normalizes data on each microarray slide by local regression of log_2 ratio against intensity using weighted linear least squares and a 2nd degree polynomial model. This model is used to calculate normalized expression values for each gene.

$$N\left(log_2\left(\frac{R_i}{G_i}\right)\right) \to log_2\left(\frac{R_i}{G_i}\right) - f\left(log_2\left(\frac{R_i}{G_i}\right)\right)$$

$$f\left(log_2\left(\frac{R_i}{G_i}\right)\right) = LOESS\left(log_2\sqrt{R_i \cdot G_i}, log_2\left(\frac{R_i}{G_i}\right)\right)$$

Robust LOESS (quadratic fit) — Robust LOESS normalization normalizes data on each microarray slide by local regression of $\log_2$ ratio against intensity using weighted linear least squares and a 2nd degree polynomial model. The robust version of LOESS performs additional fitting iterations and assigns lower weight to outliers in the regression. The method assigns zero weight to data outside six mean absolute deviations. This model is used to calculate normalized expression values for each gene. Robust LOESS needs more time to complete than simple LOESS but produces results more robust against possible outliers.

$$N\left(log_2\left(\frac{R_i}{G_i}\right)\right) \to log_2\left(\frac{R_i}{G_i}\right) - f\left(log_2\left(\frac{R_i}{G_i}\right)\right)$$

$$f\left(log_2\left(\frac{R_i}{G_i}\right)\right) = RobustLOESS\left(log_2\sqrt{R_i \cdot G_i}, log_2\left(\frac{R_i}{G_i}\right)\right)$$

Rank Invariant — Rank Invariant normalization normalizes data on each microarray slide by selecting a number of genes which are non-differentially expressed, fit a normalization curve through these genes and use this curve coupled with interpolation methods to normalize the genes present on the slide. Genes are ranked based on the signal intensities of the two channels and the rank invariant set of genes is determined by those genes whose proportional rank difference is smaller than a given threshold. Rank Invariant normalization is useful especially when data on a microarray slide appear not to be very homogeneous (e.g. the histogram of expression is bimodal).

$$N\left(log_2\left(\frac{R_i}{G_i}\right)\right) \to log_2\left(\frac{N(R_i)}{G_i}\right)$$

$$N(R_i) \to RankInvariant(G_i, R_i)$$

No normalization — If this option is selected, ARMADA will continue without performing any data normalization. It is highly not recommended unless the user wishes to perform a comparison study or any other purposes that include un-normalized data.

$$N\left(log_2\left(\frac{R_i}{G_i}\right)\right) \to log_2\left(\frac{R_i}{G_i}\right)$$

If Rank Invariant normalization is selected, the following window will appear prompting the user to set certain parameters (or leave the default values unchanged):

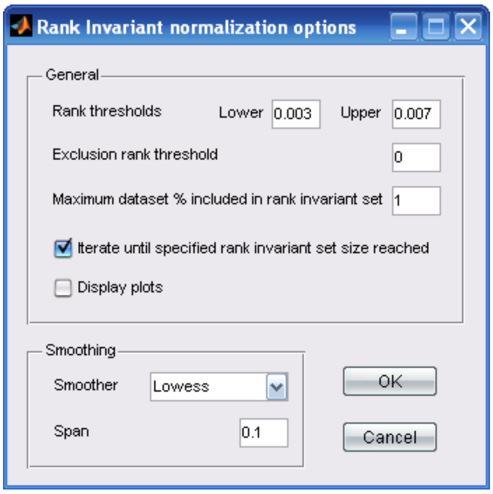

In the **General** panel the user should set parameters concerning the determination of the rank invariant set of genes while in the **Smoothing** panel, the type of smoothing curve that will be fit using the set of rank invariant genes and will be used to normalized the genes on the array. The following table describes the several parameters and their values:

**Parameter**	**Description**
Rank thresholds	The thresholds for the lowest average rank and the highest average rank, which are used to determine the invariant set. The rank invariant set is a set of data points whose proportional rank difference is smaller than a given threshold. These two thresholds are usually being set empirically to limit the spread of the invariant set, but should allow enough data points to determine the normalization relationship. Parameter values must lie between 0 and 1.
Exclusion rank threshold	This parameter filters the invariant set of data points, by excluding genes whose average rank (between 'Channel 2' or 'Red' or 'Cy5' and 'Channel 1' or 'Green' or 'Cy3') is in the highest N ranked averages or lowest N ranked averages. This parameter is useful if the user wishes to exclude rank invariant genes whose ranks are very 'high' or very 'low' respectively, in order to ensure that this genes won't affect the normalization curve as 'outliers'.
Maximum dataset % included in rank invariant set	This parameter stops the rank invariant set definition iterative process when the number of genes in the invariant set reaches *x* percent of the total number of array genes. If set to 0, the iterative process continues until no more genes from the array under process are eliminated.
Iterate until specified rank invariant set size reached	This option controls the iterative process which determines the rank invariant set of genes. When checked, the rank invariant selection algorithm repeats the process until either no more genes are eliminated, or a predetermined percentage of genes (**Maximum dataset % included in rank invariant set**) is reached. When unchecked, the algorithm performs only one iteration of the process.
Display plots	This option controls whether to display MA (Ratio-Intensity) plots of un-normalized and rank invariant normalized genes of the slide under process after the completion of the procedure.
Smoother	This option defines the method that will be used to fit a curve using the rank invariant set of genes determined by the rank

invariant selection algorithm. The available methods are **Lowess**, **Running Mean** and **Running Median**. For a description of Lowess the user should see also the LOWESS/LOESS normalization descriptions.

Span — The span value modifies the running window size (proportion of neighbouring points to the currently processed point) for the smoothing function. If the span value is less than 1, the window size is taken to be a fraction of the number of points in the data. If span value is greater than 1, the running window contains as many data points as the value given.

For more details concerning Rank Invariant normalization, the user should see (4).

Concerning the **Dye-Swap options** panel in the normalization window, the list **Channel – Dye correspondence** is used to determine which channel corresponds to which dye, for example, if the reference samples were labeled with Cy3 prior to hybridization it should be assigned to 'Channel 1' (and Cy5 to 'Channel 2'). Typically, reference samples are labeled with Cy3 and treated samples with Cy5 which is also the default setting to ARMADA. If for any reason Cy3 corresponds to Channel 2, this should be declared by choosing **Cy3 is channel 2**. Alternative, if there is a dye-swap experimental design, the user can press the **Launch Dye-Swap editor** button and the following window will appear:

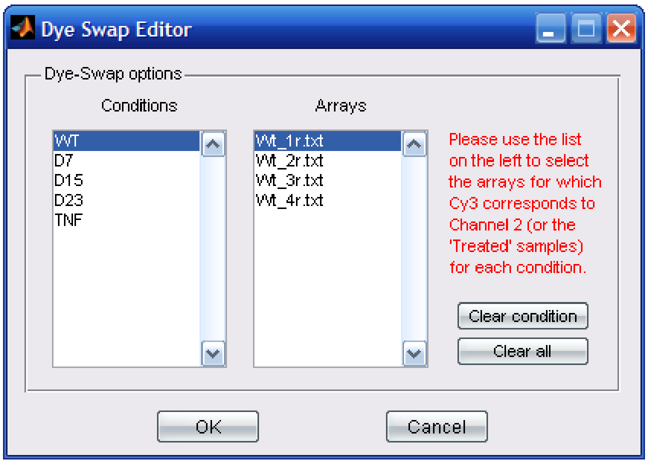

From there, the user can select which arrays from which experimental condition correspond to a dye-swap hybridization. In other words, the user should select only the arrays for which Cy3 (or 'Green') corresponds to Channel 2, or the channel user for treated samples. If the user makes a mistake in the selection of the arrays, the **Clear condition** and **Clear all** buttons will reset the arrays for the selected condition or all the arrays to the default which is Cy3 corresponding to Channel 1 or 'Control' channel. After finishing with the selection of dye-swapped arrays, the user should click **OK**. Attention should be paid to the dye-swap options as they affects directly the calculation of the $\log_2$ ratio between channels and thus gene expression.

Concerning the **Other** panel in the normalization window, if subgrid meta-coordinates are present on the slides, the user is given the choice to select subgrid normalization by checking the **Subgrid normalization** box to possibly allow considering several spatial dependent properties such

as local background noise (caused by the robotic printing of the arrays) in the normalization procedure instead of performing normalization on the whole slide. Finally, if the user checks the **Display timebar** box, a timebar will be displayed presenting the progress and the remaining time of the normalization procedure. While a timebar gives an estimate of the time required for normalization (especially for large datasets), it consumes computer memory resources causing the normalization procedure to take more time, thus the default choice is not to display a timebar.

After making the necessary selections, the user should click **OK** to normalize the data. Depending on the normalization algorithm chosen, the normalization procedure might take some time. At this point it should be noted that the **Statistics** menu will not be enabled if normalization is not performed. ARMADA workflow does not allow performing any statistical tests without having normalized the data first in order to scale them and be able to make rational comparisons among different experimental conditions. This does not apply when the users has imported external data for process unless those data are not normalized.

4. Statistical Operations

After completing data preprocessing and normalization which excludes poor quality spots and scales data within each array, a proper statistical test can reveal several genes which are statistically distinguished among different experimental configurations. The result of statistical selection is usually a far smaller set of genes (compared to the initial dataset) which provides valuable information about the experiment. Gene expression pattern analysis can be also performed using a clustering algorithm in order to reveal genes belonging to groups with common expression. This section presents the statistical selection and clustering methods implemented in this platform, along with how these can help the user reaching to a conclusion.

4.1. Statistical Selection

This section presents the statistical tests supported by ARMADA for the extraction of differentially expressed gene lists as well as the statistical analysis workflow which includes the *Trust Factor* filtering, between slide normalization, imputation of possible missing values caused by image analysis, user flagging or filtering steps and multiple statistical testing correction methods. In order to perform statistical analysis, the user should click **Statistics** → **Statistical Selection.** This will bring up the **Statistical Selection** preferences window which is shown below:

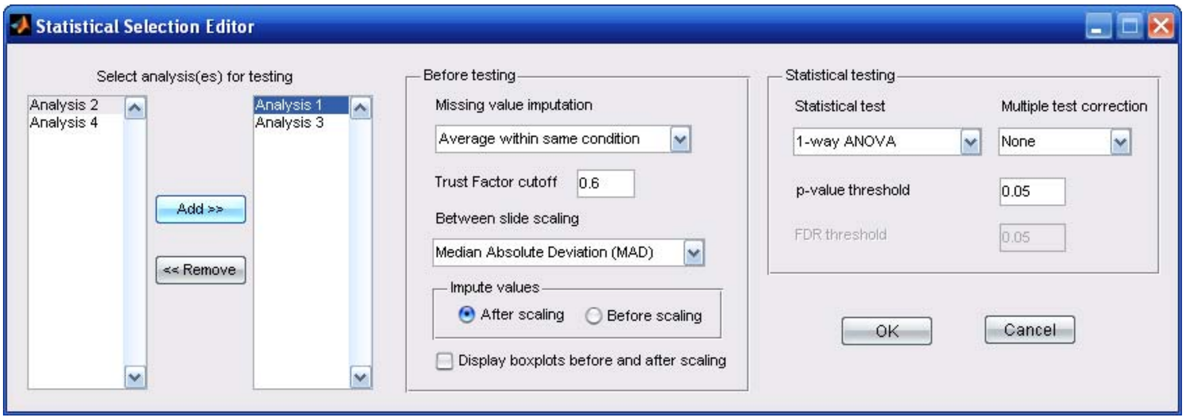

In this window, the user can set up the parameters that best fit his needs or leave the defaults. In the left side of the window, there are two lists. The left one contains the pool of available Analysis objects created so far and the right list contains the Analysis object for which the user wishes to perform statistical testing. Different Analysis objects can be added to or removed from the list for testing by clicking on the **Add** >> or << **Remove** buttons. All the parameters can be set by the options available in the **Before testing** and **Statistical testing** panels. The following table describes the available options in the **Before testing** panel:

**Option**	**Description**
Missing value imputation	This option determines how missing values from the dataset will be imputed. If **Average within the same condition** is selected, then

	missing values for each gene are imputed per experimental condition by averaging the average expression of the remaining present values of the gene of interest from the same experimental condition. If **k-nearest neighbor (kNN)** is selected, then missing values are imputed using the whole dataset used a kNN based value estimation (5).
Trust Factor cutoff	The Trust Factor is defined for each experimental condition as: *TF* = *#Appearances/#Replicates*. The number of appearances for each gene is determined by the initial filtering steps: for example, if one gene in a specific slide is found sensitive either to any of the filters applied, then it is marked as absent. If one gene is filtered out from all replicates for a given condition, then the TF for this gene is zero. This gene is then marked as 'unreliable' and is excluded from further analysis. The user is prompted to supply a cutoff or leave the default value.
Between slide scaling	This option determines the between slide normalization method. If **Median Absolute Deviation (MAD)** is selected, then expression values among all arrays for the selected Analysis will be scaled using the MAD. MAD is defined as $MAD = median\left(Y_i - \tilde{Y}\right)$ where $\tilde{Y}$ is the median of the data and $\lvert Y \rvert$ is the absolute value of *Y*. This is a variation of the average absolute deviation that is less affected by extremes in a distribution tail because the data in the distribution tails have less influence on the calculation of the median than they do on the mean. This value is calculated for each condition of the selected Analysis and then subtracted from it in order to make data more easily compared. If **Quantile normalization** is selected, then data are normalized between slides using the Quantile normalization algorithm (6). If **No scaling** is selected, then no between slides normalization is performed.
Impute values	This option determines whether missing value imputation will take place before (**Before scaling**) or after (**After scaling**) between slide normalization. It is recommended to perform missing value imputation after between slide normalization as data are standardized.
Display boxplots before and after scaling	If this box is checked, a boxplot (the user should see also section 5.5) will be displayed, depicting the distributions of the data before and after between slide normalization.

In the case of selecting **k-nearest neighbor (kNN)** in the **Missing value imputation** options list, the following window will appear prompting the user to set some parameters for the imputation:

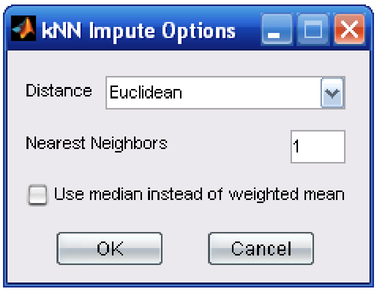

The following table explains each option and parameter:

**Option**	**Description**
Distance	Determines the distance metric that will be used to calculate the distances among gene vectors determined by the gene expression values for all experimental conditions in the selected Analysis. For more

	information on distance metrics, the user should see Appendix D.
Nearest Neighbors	The number of nearest neighbors that will be used to impute the missing values.
Use median instead of weighted mean	If checked, missing values will be imputed based on the median of the nearest neighbors expression values instead of the weighted mean.

In the case of selecting **Quantile normalization** in the **Between slide scaling** options list, the following window will appear prompting the user to set some parameters for scaling:

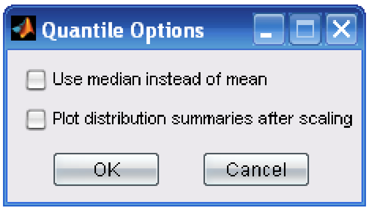

The following table explains each option and parameter:

**Option**	**Description**
Use median instead of mean	If checked, the median of the ranked values will be used instead of the mean.
Plot distribution summaries after scaling	If checked, a plot presenting the gene expression distributions among all slides of the selected Analysis and the summary quantiles distribution will be displayed.

In the **Statistical testing** panel, the user can select the test to be performed, select a multiple test correction method and set the cutoff values that will determine which genes are statistically differentially expressed. The following table describes the available options in the **Statisticaltesting** panel:

**Option**	**Description**
Statistical test	Available statistical tests are 1-way ANOVA (ref. here, Kerr), Kruskall Wallis (non-parametric equivalent of ANOVA), t-test and Time Course ANOVA. Time Course ANOVA should be used when each experimental configuration has its own control (e.g. when performing a time course experiment, where one possible configuration is that each separate time point has its own control). In this case, ANOVA is performed among each point's fold changes instead of among expression values.
Multiple test correction	Available multiple testing correction methods, are the following:<br>None: No multiple testing correction.<br>Bonferroni: Bonferroni multiple testing correction procedure (7).<br>FDR Benjamini-Hochberg: Benjamini-Hochberg False Discovery Rate control procedure (8).

pFDR Storey (Boostrap)	Storey positive False Discovery Rate control procedure. True null hypothesis is calculated from the tuning parameter based on bootstrap (9).
pFDR Storey (Polynomial)	Storey positive False Discovery Rate control procedure. True null hypothesis is calculated from the tuning parameter based on polynomial fit (9).

The user should consult Appendix C for more information on multiple testing correction issues.

p-value threshold	A p-value cutoff threshold (when no or Bonferroni multiple testing correction chosen) to determine the number of differentially expressed genes.
FDR threshold	An FDR threshold value (when all other multiple testing correction methods chosen) to determine the number of differentially expressed genes.

In the case of selecting **Time Course ANOVA** in the **Statistical test** options list, the following window will appear:

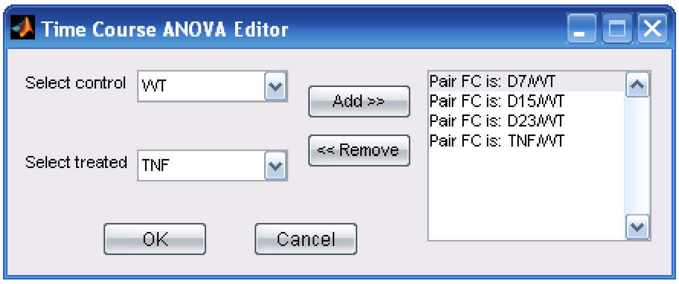

In this preferences window, the user should define pairs of experimental conditions so that fold changes can be calculated for time course ANOVA to run. The user can define pairs by using the **Select control** and **Select treated** lists and using the **Add** >> and << **Remove** buttons to add or remove pairs respectively.

After setting all the desired parameters in the statistical selection preferences window, the user should clink **OK**. It should be noted that the user can define a different workflow for each Analysis in the right list by selecting the Analysis object and setting the desired parameters. After clicking **OK**, ARMADA will start the statistical selection process for each of the selected Analysis objects. For each Analysis object, the following window will appear:

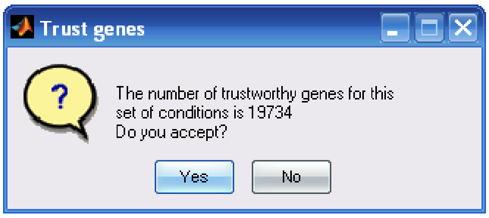

This is the result of the Trust Factor cutoff. The user should click **Yes** or **No** depending on preference. If **No** is clicked, ARMADA will stop the process for the Analysis object under processing and will jump to the next one. If the user click **Yes**, the following window will appear after the application of a statistical test:

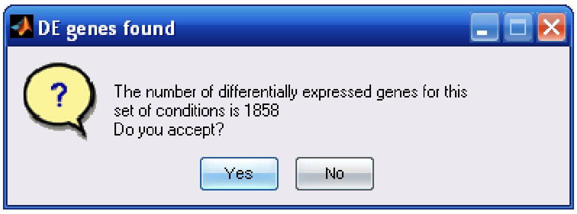

This is the result of the statistical test concerning the number of differentially expressed genes found. If the user clicks **Yes**, ARMADA stores the results and continues with the next Analysis object. If the user clicks **No**, ARMADA will skip the result for the Analysis object under processing and will jump to the next one. Differentially expressed genes can be viewed by hitting the **DE List** button on the main window.

4.2. Fold Change Calculation

Apart from statistical testing which leads to statistical score values, fold changes can provide useful estimations on how much a gene is differentiated compared to its control or other conditions. In order to calculate fold changes, the user should select an Analysis object from the Analysis Objects list and click **Statistics** → **Fold Change Calculation** and the following window will appear:

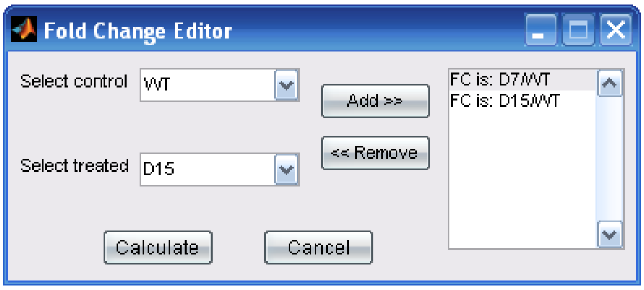

In this preferences window, the user should define pairs of experimental conditions so that fold changes can be calculated. The user can define pairs by using the **Select control** and **Select treated** lists and using the **Add** >> and << **Remove** buttons to add or remove pairs respectively. The **Select control** and **Select treated** lists contain the names of the experimental conditions of the Analysis object selected in the Analysis Object list. After finishing with pair assignment, the user should click **Calculate** to calculate fold changes based on the assigned pairs.

4.3. Clustering

The term *cluster analysis* or *clustering* encompasses a number of different algorithms and methods for grouping objects of similar kind into respective categories. A general question facing researchers in many areas of inquiry is how to organize observed data into meaningful structures. In other words cluster analysis is an exploratory data analysis tool which aims at sorting different objects into groups in a way that the degree of association between two objects is maximal if they belong to the same group and minimal otherwise. Given the above, cluster analysis can be used to discover structures in data without providing an explanation/interpretation. In other words, cluster analysis simply discovers structures in data without explaining why they exist.

Clustering differentially expressed genes in a microarray experiment helps scientists to organize these genes into groups. Genes that belong to the same group might have something in common about how they are activated or even the way they interact with each other. For example, a group of similarly expressed genes might affect the transcription of proteins that catalyze reactions from specific pathway, and thus giving clues to the scientists to further examine this particular pathway of the cell cycle. There are several methods of clustering; ARMADA supports hierarchical, k-means and fuzzy C-means clustering. This section presents how the user can perform cluster analysis using one of the three mentioned methods.

4.3.1. Hierarchical clustering

In hierarchical clustering the data are not partitioned into a particular cluster in a single step. Instead, a series of partitions takes place, which may run from a single cluster containing all objects to *n* clusters each containing a single object. Hierarchical clustering may be represented by a two dimensional diagram known as clustergram (or clustering heatmap) which illustrates the fusions or divisions made at each successive stage of analysis. An example of such a clustergram is given below:

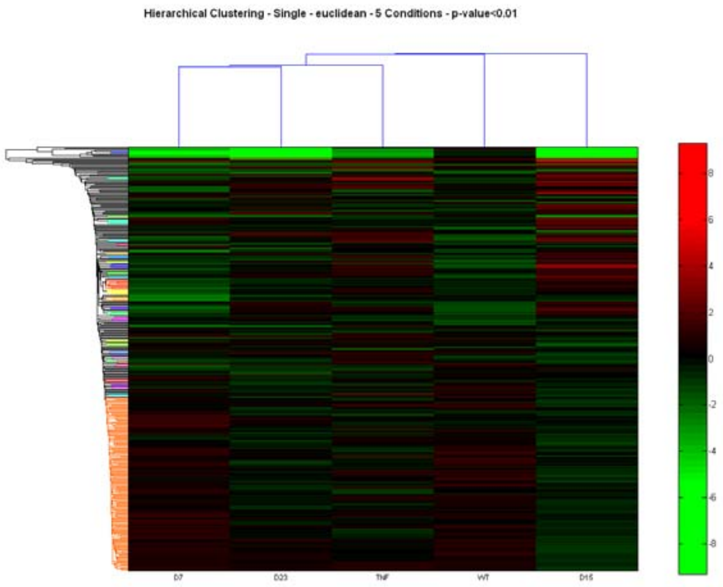

For further information concerning hierarchical clustering, the user should see (10). In order to perform hierarchical clustering the user should select an Analysis object from the Analysis Objects list and click **Statistics** → **Clustering** → **Hierarchical**. The following preferences window will appear:

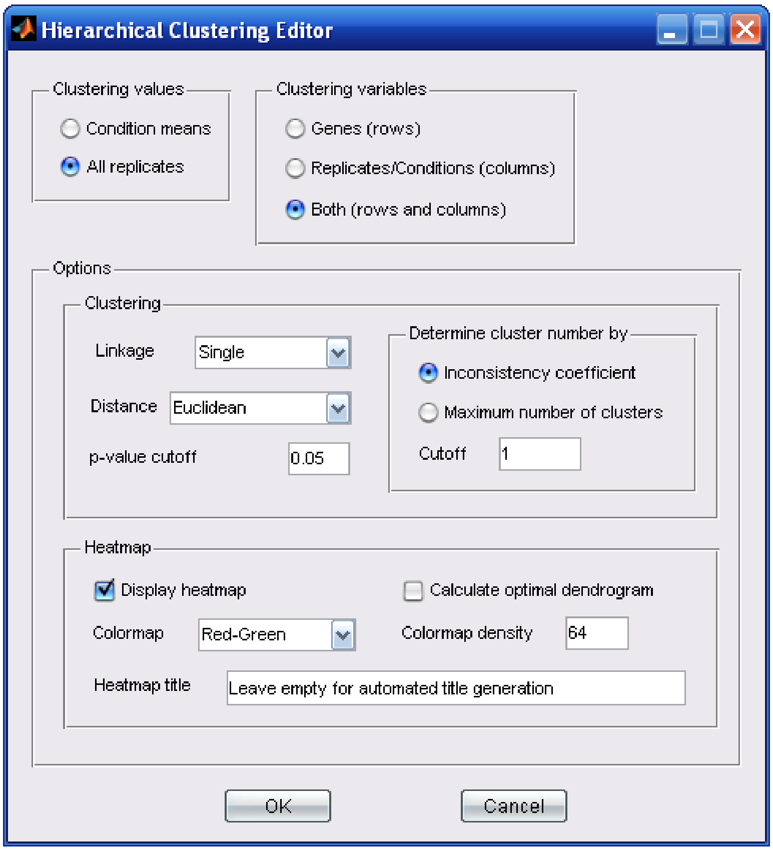

The following table explains the available options in the two upper panels, **Clustering values** and **Clustering variables**:

	**Option**	**Description**
**Clustering values**	Condition means	If selected, gene expression values to be clustered are the mean expression value among replicates for each condition of the selected Analysis.
	All replicates	If selected, gene expression values to be clustered are all the values from all array replicates from each condition of the selected Analysis. This option sometimes serves also as quality control of an experiment. If all replicates of an experimental condition are not clustered together, the missing replicate might be of low quality.
**Clustering variables**	Genes (rows)	If selected, hierarchical clustering will be performed for genes, revealing clusters of genes with similar expression.
	Replicates/Conditions (columns)	If selected, hierarchical clustering will be performed for conditions or replicates (depending on the choice in the **Clustering values** panel), revealing clusters of conditions.
	Both (rows and columns)	If chosen, hierarchical clustering will be applied to both genes and conditions/replicates, constructing thus a tree clustering diagram (dendrogram) for both genes and replicates or conditions.

The following table explains the available options in the bottom panel, **Options**:

	Option	Description
Clustering	Linkage	The linkage algorithm to be used for data clustering (for further information the user should see Appendix D on distances and linkages).
	Distance	The distance metric to be used for data clustering (for further information the user should see Appendix D on distances and linkages).
	p-value cutoff	A p-value cutoff, additional to the statistical test p-value cutoff, in order to cluster fewer genes than those determined by the statistical test. For example, if the statistical test was performed with a p-value cutoff of 0.05, the user can enter 0.01 to cluster fewer genes than those determined by the cutoff of 0.05.
	Inconsistency coefficient	The inconsistency coefficient cutoff to determine the number of clusters based on the dendrogram[6] (11).
	Maximum number of clusters	The maximum number of clusters to which the dataset can be grouped into.
	Cutoff	Either the inconsistency coefficient cutoff or the maximum number of clusters.
Heatmap	Display heatmap	If checked, a clustering heatmap will be displayed.
	Calculate optimal dendrogram	If checked, the dendrogram on the clustering heatmap will be optimized for better clustering results. However, it can take a considerable amount of time.
	Colormap	The user should see section 5.1.
	Colormap density	The user should see section 5.1.
	Heatmap title	A title for the heatmap to be created, if chosen.

After setting the desired parameters (or leave the defaults) the user should click **OK**. Hierarchical clustering will be performed and ARMADA will store the result. Gene clusters can be viewed by hitting the **Cluster List** button on the main window.

4.3.2. k-means clustering

k-means clustering is one of the simplest algorithms that solves the well known clustering problem. It differs from hierarchical clustering in that the number of clusters, *k*, needs to be determined at the onset. The goal is to divide the objects into *k* clusters such that some metric relative to the centroids (cluster centers) of the clusters is minimized. These centroids should be placed in a skillful way because different location causes different results. The better choice is to place them as much as possible far away from each other. The next step is to take each point belonging to a given data set and associate it to the nearest centroid. When no point is pending, the first step is completed and an early grouping is done. At this point re-calculation of k new centroids should be performed based on the results of the first grouping. This procedure is repeated until the

[6] Only one among **Inconsistency coefficient** and **Maximum number of clusters** can be provided to determine the number of returned clusters.

centroids stop changing positions. k-means clustering is very useful when there is an *a priori* estimation of the number of clusters that the data should be grouped into. For further information on k-means clustering, the user should see (10) In order to perform k-means clustering, the user should select an Analysis object from the Analysis Objects list and click **Statistics** → **Clustering** → **k-means**. The following preferences window will appear:

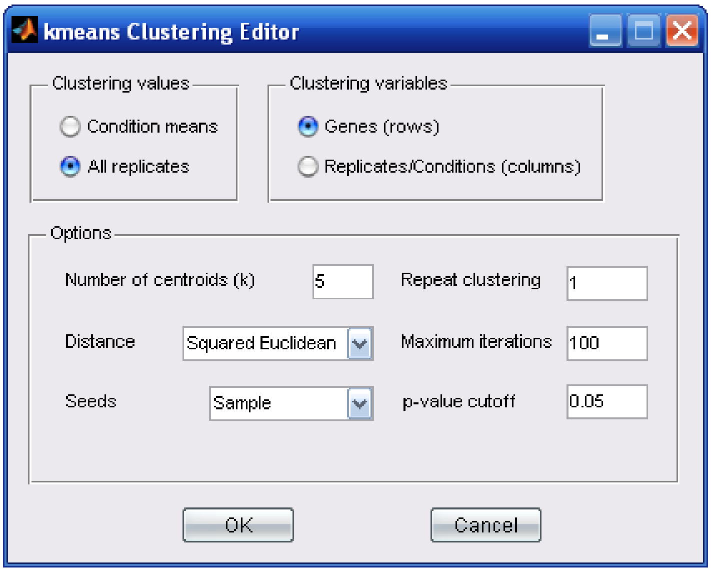

The following table explains the available options in the two upper panels, **Clustering values** and **Clustering variables**:

	**Option**	**Description**
**Clustering values**	Condition means	If selected, gene expression values to be clustered are the mean expression value among replicates for each condition of the selected Analysis.
	All replicates	If selected, gene expression values to be clustered are all the values from all array replicates from each condition of the selected Analysis.
**Clustering variables**	Genes (rows)	If selected, hierarchical clustering will be performed for genes, revealing clusters of genes with similar expression.
	Replicates/Conditions (columns)	If selected, hierarchical clustering will be performed for conditions or replicates (depending on the choice in the **Clustering values** panel), revealing clusters of conditions.

The following table explains the available options in the bottom panel, **Options**:

**Option**	**Description**
Number of centroids (k)	The number of clusters that the genes should be grouped into.
Distance	The distance metric to be used for data clustering (for further information the user should see Appendix D on distances and linkages).
Seeds	Method used to choose the initial cluster centroid positions. The following methods are available:
	Sample — If selected, k observations from the data matrix to be clustered are selected randomly to be the initial centroids.

	Uniform	If selected, k random points are selected uniformly from the range of the data matrix to be clustered.
	Clusters	If selected a preliminary clustering phase is performed on a random 10% subsample of the data matrix to be clustered. This preliminary phase is itself initialized using the 'Sample' option.
Repeat clustering	Number of times to repeat the clustering process, each with a new set of initial cluster centroid positions. The solution with the smallest distance between clusters is returned.	
Maximum iterations	Maximum centroid convergence iterations.	
p-value cutoff	A p-value cutoff, additional to the statistical test p-value cutoff, in order to cluster fewer genes than those determined by the statistical test. For example, if the statistical test was performed with a p-value cutoff of 0.05, the user can enter 0.01 to cluster fewer genes than those determined by the cutoff of 0.05.	

After setting the desired parameters (or leave the defaults) the user should click **OK**. k-means clustering will be performed and ARMADA will store the result. Gene clusters can be viewed by hitting the **Cluster List** button on the main window. The user can also consult section 5.7 on how to plot gene expression profiles for clusters formed with the k-means algorithm.

4.3.3. Fuzzy C-means clustering

While partitional clustering methods such as k-means or hierarchical clustering assign each gene to a single cluster, these methods do not provide information about the influence of a given gene for the overall shape of clusters. Fuzzy partitioning methods such as fuzzy c-means clustering can solve this problem by attributing cluster membership values to genes. The cluster where the membership for each gene is highest is probably the cluster which it belongs to, but there is the possibility to check what happens with possible membership to other clusters by looking other memberships for specific genes. For more information on fuzzy c-means clustering as well as the algorithm implemented in ARMADA, the user should see (12). In order to perform fuzzy c-means clustering, the user should select an Analysis object from the Analysis Objects list and click **Statistics → Clustering → Fuzzy C-Means**. The following preferences window will appear:

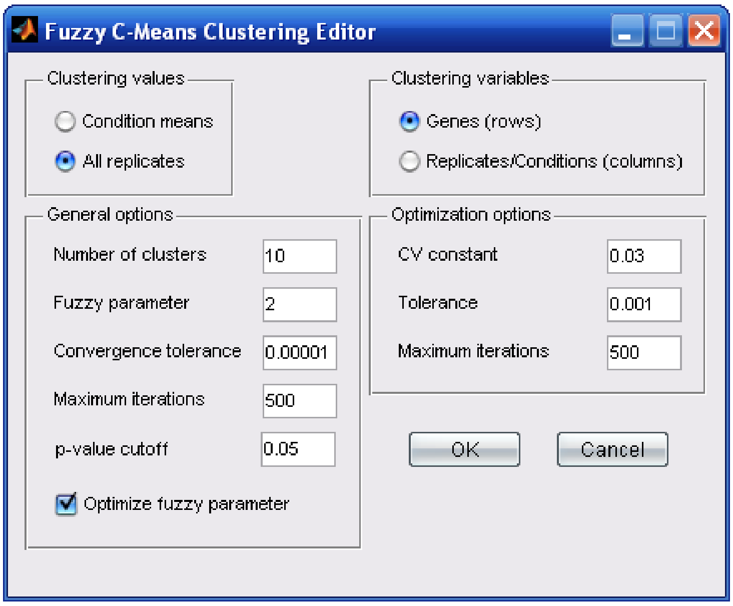

The following table explains the available options in the two upper panels, **Clustering values** and **Clustering variables**:

	**Option**	**Description**
**Clustering values**	Condition means	If selected, gene expression values to be clustered are the mean expression value among replicates for each condition of the selected Analysis.
	All replicates	If selected, gene expression values to be clustered are all the values from all array replicates from each condition of the selected Analysis.
**Clustering variables**	Genes (rows)	If selected, hierarchical clustering will be performed for genes, revealing clusters of genes with similar expression.
	Replicates/Conditions (columns)	If selected, hierarchical clustering will be performed for conditions or replicates (depending on the choice in the **Clustering values** panel), revealing clusters of conditions.

The following table explains the available options in the bottom panel, **Options**:

	**Option**	**Description**
**General options**	Number of clusters	The number of clusters that the genes should be grouped into.
	Fuzzy parameter	The parameter in fuzzy c-means clustering algorithm
	Convergence tolerance	The maximum error allowed between two consecutive values of the constrained fuzzy partition matrix (cluster membership matrix).
	Maximum iterations	Maximum number of iterations for centroid convergence.
	p-value cutoff	A p-value cutoff, additional to the statistical test p-value cutoff, in order to cluster fewer genes than those determined by the statistical test. For example, if the statistical test was performed with a p-value cutoff of 0.05, the user can enter 0.01 to cluster fewer genes than those determined by the cutoff of 0.05.
	Optimize fuzzy parameter	If checked, fuzzy parameter value will be optimized as proposed in (12).

Optimization options	CV constant	**C**oefficient of **V**ariation of the set of distances between genes (the user should see (12)).
	Tolerance	Allowed tolerance to be used in the fuzzy parameter optimization algorithm.
	Maximum iterations	Maximum number of iterations for the convergence of the fuzzy parameter.

After setting the desired parameters (or leave the defaults) the user should click **OK**. Fuzzy c-means clustering will be performed and ARMADA will store the result. It should be noted that if the box **Optimize fuzzy parameter** is checked, the running time of the algorithm might increase considerably (depending on the size of the dataset and the number of clusters). Gene clusters and cluster memberships can be viewed by hitting the **Cluster List** button on the main window. The user can also consult section 5.7 on how to plot gene expression profiles for clusters formed with the fuzzy c-means algorithm. For a complete description of the parameters in the table above as well as the fuzzy c-means clustering algorithm, the user should see (12).

4.4. Classification

Apart from classical statistical methods used for the selection of differentially expressed genes, several other techniques are used, derived especially from the area of Machine Learning for the classification of genes and the prognosis and prediction of new data. These methods are mainly classified into two categories: *unsupervised* learning and *supervised* learning. In unsupervised learning, the algorithm is given a set of objects and is trying to group them into classes without any prior knowledge of these classes or any labeled output. Classical unsupervised techniques are clustering techniques (the user should see section 4.3). Supervised learning algorithms make use of a set of classified examples and they are trying, given this sample of input-output pairs, to determine the function that maps any input to any output such that disagreement with future input-output pairs is minimized. Supervised learning usually refers to *classification* problems; the term classification usually refers to a prediction or learning problem in which the variable to be predicted assumes one of *k* unordered values, (c_1, c_2, …, c_k), arbitrarily relabeled as (1, 2, …, k) or sometimes (0, 1, …, k-1). The k values correspond to k predefined classes, e.g., tumor class or bacteria type. Classification algorithms are given a set of samples and their class label and try to predict the correct class for new data and regression problems where the output is a set of real numbers instead of class labels (10). Classification algorithms supported in ARMADA are (Linear) Discriminant Analysis, k-Nearest Neighbor algorithms (kNN) and Support Vector Machines (SVM) classification.

The performance of classifiers can be evaluated by several techniques. Three of them are supported in ARMADA:

i) N-fold cross validation. In this technique, the *p* data used to train the classifier are randomly split in *n* independent datasets of size approximately *p*/*n*. Subsequently, *n* rounds of validation

follow, where in each round, *n-1* datasets are used to train the classifier and *1* to test it. The misclassification error is the average number of misclassified instances.

ii) Leave-m-out. In this technique, *m* rounds of classification are performed, where in each round, *m* samples are left out from the dataset in order to be used later to validate the classifier built with the rest *p-m* samples. A widely used value for *m* is *m=1*. The misclassification error is the average number of misclassified instances.

iii) Training-and-test. In this technique, a percentage of the dataset is held out and the rest is used to train the classifier. The misclassification error is the average number of misclassified instances from the held-out part of the dataset.

At this point, the user should note that usually, prior to building a classifier using any classification technique, the number of variables used as variables that can discriminate among classes, have to be reduced in order to remove noise. For example, in a microarray experiment where microarrays contain several thousands of genes, not all of them are differentially expressed among different experimental conditions (classes) and they only add noise to the experiment. This procedure of noise removal in classification procedures is called *feature selection*. Feature selection can be performed in ARMADA by any of the statistical selection methods that are supported (section 4.1). For this reason, classification algorithms become available in ARMADA only after the statistical selection procedure. The rest of this section presents how the user can build and use classifiers in ARMADA. For more information on classification, feature selection and classifier evaluation techniques, the user should see (13).

4.4.1. (Linear) Discriminant Analysis

Discriminant Analysis (DA) may be used for two objectives: either to assess the adequacy of classification, given the group memberships of the objects under study, or to assign objects to one of a number of (known) groups of objects. DA may thus have a descriptive or a predictive objective. In both cases, some group assignments must be known before carrying out the DA. Such group assignments, or labelling, may be arrived at in any way. For example, in microarray classification studies, one group might represent samples from healthy tissues while the other(s) from diseased tissues. For more information on DA the user should see (10). Apart from building a DA classifier, ARMADA offers a batch process module that allows the user to tune the classifier (e.g. select the best discriminant function type of class prior probabilities) for a specific problem studied using a representative dataset. The following sub-sections describe the tuning and classification process using DA in ARMADA.

4.4.1.1. (Linear) Discriminant Analysis – Tuning

In order to perform DA classifier tuning, the user should select an Analysis object from the Analysis Objects list and click **Statistics → Classification → Discriminant Analysis → Tune**. The following preferences window will appear:

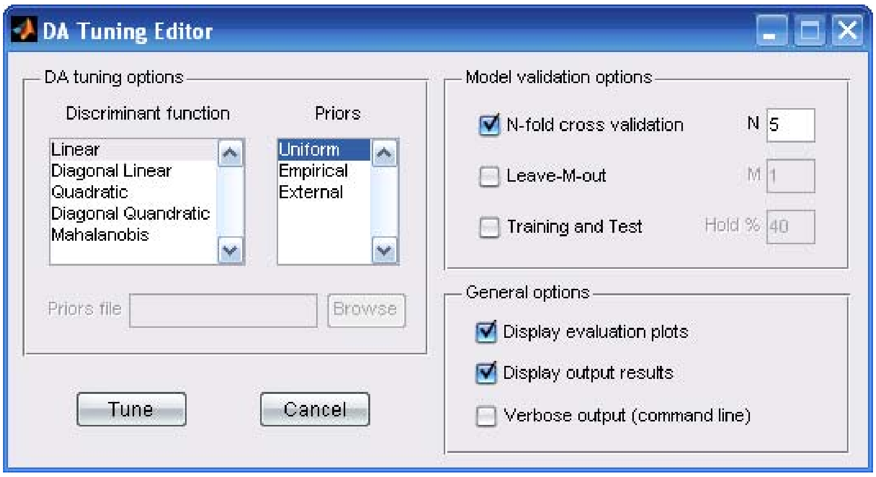

The following table explains the available options in the **DA tuning options**, **Model validation options** and **General options** panels:

	**Option**	**Description**
**DA tuning options**	Discriminant function	The type of discriminant function. The following types are available (descriptions partially from MATLAB's help):<br>Linear — Fits a multivariate normal density to each group, with a pooled estimate of covariance.<br>Diagonal Linear — Similar to Linear, but with a diagonal covariance matrix estimate (naive Bayes classifiers).<br>Quadratic — Fits multivariate normal densities with covariance estimates stratified by group.<br>Diagonal Quadratic — Similar to Quadratic, but with a diagonal covariance matrix estimate (naive Bayes classifiers).<br>Mahalanobis — Uses Mahalanobis distances with stratified covariance estimates.
	Priors	The way that prior class probabilities are defined. The following options are available:<br>Uniform — Prior probabilities are derived from a uniform distribution (equal prior probabilities for each class).<br>Empirical — Class prior probabilities are estimated from the group relative frequencies in the training dataset.<br>External — User-defined class prior probabilities; in this case, the prior probabilities are given in a text tab delimited or Excel file.

	Priors file	A text tab delimited or Excel file containing class prior probabilities structured as follows: the 1st column should contain the class names and the 2nd the prior probabilities corresponding to each class in the 1st column (the user should also see Appendix A for an example).
**Model validation options**	N-fold cross validation	The user should check this box to perform N-fold cross validation of the classifier and supply N.
	Leave-M-out	The user should check this box to perform Leave-M-out validation of the classifier and supply M.
	Training and Test	The user should check this box to perform Training and Test validation of the classifier and supply the percentage of the dataset that should be held out for testing.
**General options**	Display evaluation plots	Displays classifier evaluation plots based on the tuning options and parameters (plots are based on the discriminant function types, class prior probabilities and validation methods).
	Display output results	A report containing classification evaluation statistics and confusion tables[7] is displayed in a separate window.
	Verbose output (command line)	Several messages are displayed during the classifier tuning procedure in the command line or in MATLAB's command window if MATLAB is present.

After setting the parameters, the user should click **Tune** and classifier tuning will be performed. Depending on the DA tuning results, the user can select the appropriate parameters to build a model that best fits the dataset under examination. If the user selects to display classifier evaluation plots by checking the box **Display evaluation plots**, the following example depicts how these plots are presented.

[7] A confusion matrix is a visualization tool typically used in supervised learning. Each column of the matrix represents the instances in a predicted class, while each row represents the instances in an actual class. One benefit of a confusion matrix is that it is easy to see if the classifier is confusing two classes (i.e. commonly mislabelling one as another).

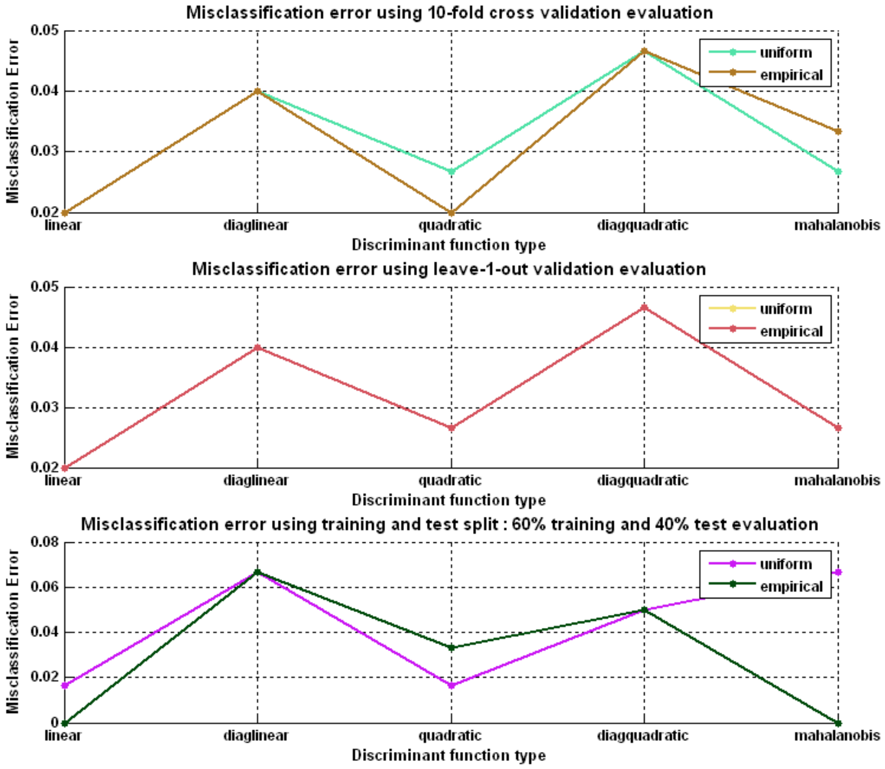

If the user selects to display a classifier evaluation report by clicking **Display output results**, a window like the following will appear presenting the classifier evaluation results:

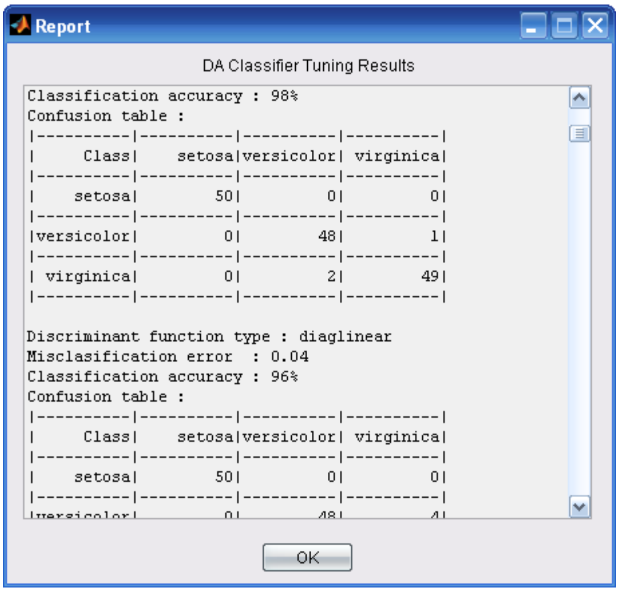

If the user right-clicks inside the report area, a context menu will appear allowing to export the report in a text tab delimited file or clear the report window.

4.4.1.2. (Linear) Discriminant Analysis – Classifying

In order to perform DA classification, the user should click **Statistics** → **Classification** → **Discriminant Analysis** → **Classify** and the following preferences window will appear:

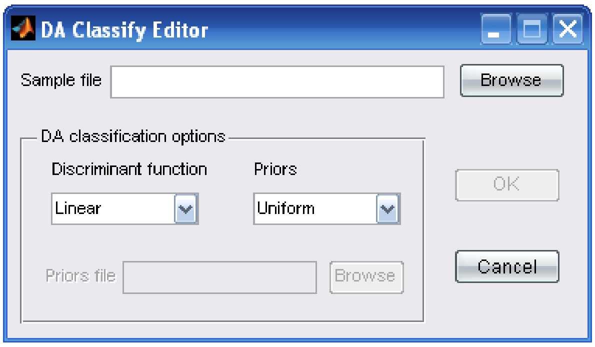

The user should select the file that contains the new samples to be classified using as training data the data imported to ARMADA. The file can be a text tab delimited or Excel file which should be structured as follows: the first column should contain variable names (e.g. gene names) that serve as unique variable identifiers. The first row should contain sample names that will be used to identify the new samples when they will be assigned to classes. The rest of the data should be numeric. Attention should be paid so that the number of variables-features is the same as the number of features used to build the classifier model. For an example of a file of new samples to be classified, the user should consult Appendix A.

The rest of the options in the DA Classify preferences window under the **DA classification options** panel (**Discriminant function**, **Priors**) are the same as in the case of tuning the DA classifier and their description can be found in section 4.4.1.1. After setting the desired parameters, the user should click **OK**. After the classification procedure, a report window will appear:

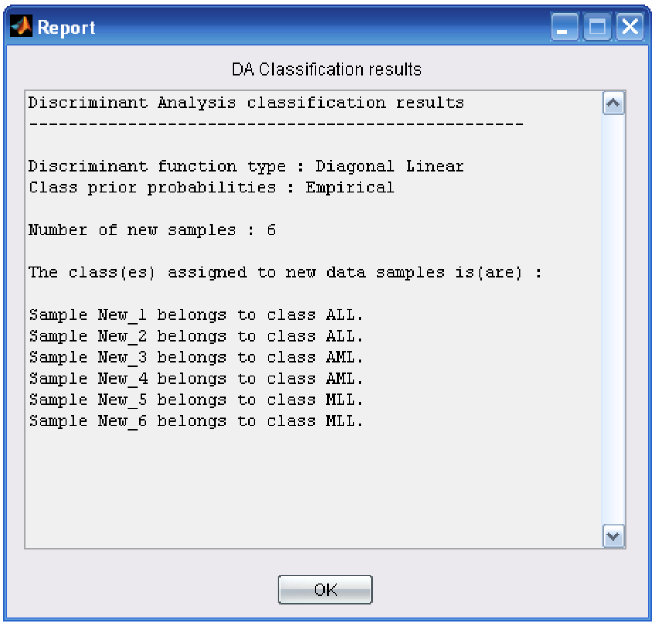

4.4.2. k–Nearest Neighbors

The k-Nearest Neighbors (kNN) classification is a very simple, yet powerful classification method. The key idea behind kNN classification is that similar observations belong to similar classes. Thus, one simply has to look for the class designators of a certain number of the nearest neighbors and weigh their class numbers to assign a class number to the unknown. The weighing

scheme of the class numbers is often a majority rule, but other schemes are conceivable. The number of the nearest neighbors, k, should be odd in order to avoid ties, and it should be kept small, since a large k tends to create misclassifications unless the individual classes are well-separated. One of the major drawbacks of kNN classifiers is that the classifier needs all available data. This may lead to considerable overhead, if the training data set is large. Apart from building a kNN classifier, ARMADA offers a batch process module that allows the user to tune the classifier (e.g. select the best number of nearest neighbors in combination with the proper distance function and tie breaking rule) for a specific problem studied using a representative dataset. The following sub-sections describe the tuning and classification process using kNN in ARMADA.

4.4.2.1. k–Nearest Neighbors – Tuning

In order to perform kNN classifier tuning, the user should select an Analysis object from the Analysis Objects list and click **Statistics → Classification → k – Nearest Neighbors → Tune**. The following preferences window will appear:

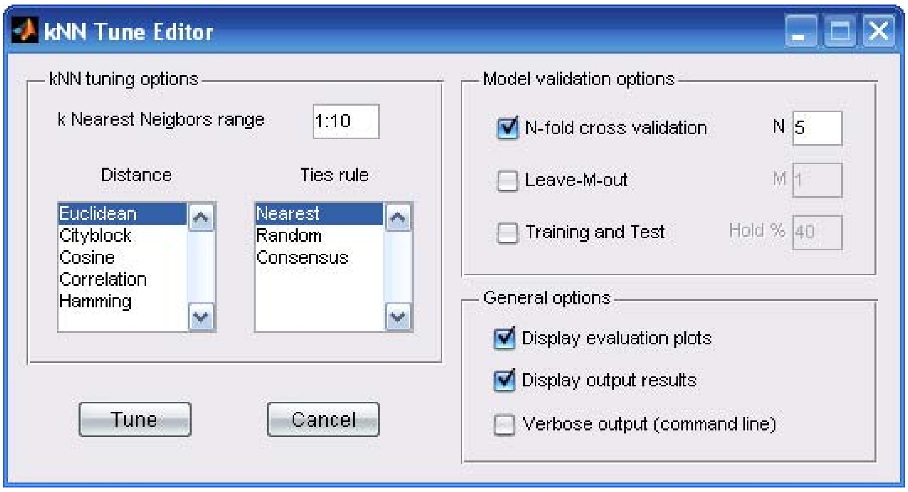

The following table explains the available options in the **kNN tuning options**, **Model validation options** and **General options** panels:

	Option	Description
**kNN tuning**	k Nearest Neigbors range	A range of values that should be used as number of nearest neighbors. It should be given as a series of numbers separated by commas or spaces, or in MATLAB's sequence number format, (e.g. 1:10 results in a range of 1-10 and 1:2:10 gives 1, 3, 5, 7, 9).

	Distance	The distance function used to calculate the distance of samples to their nearest neighbors. The following distances are available:<br>Euclidean — The Euclidean distance<br>Cityblock — The cityblock (Manhattan) distance.<br>Cosine — The cosine distance.<br>Correlation — Pearson's correlation distance.<br>Hamming — Hamming distance. It can be used only with binary data else it will generate an error.<br>For further information on distances, the user should see Appendix D.
	Ties rule	Tie breaking rules. The following rules are available:<br>Nearest — Majority rule with nearest point tie-break<br>Random — Majority rule with random point tie-break<br>Consensus — Consensus rule; when using the consensus option, points where not all of the k nearest neighbors are from the same class are not assigned to one of the classes. Because of this, it might generate errors when not used carefully.
**Model validation options**	N-fold cross validation	The user should check this box to perform N-fold cross validation of the classifier and supply N.
	Leave-M-out	The user should check this box to perform Leave-M-out validation of the classifier and supply M.
	Training and Test	The user should check this box to perform Training and Test validation of the classifier and supply the percentage of the dataset that should be held out for testing.
**General options**	Display evaluation plots	Displays classifier evaluation plots based on the tuning options and parameters (plots are based on the number of nearest neighbors, distances, tie-break rules and validation methods).
	Display output results	A report containing classification evaluation statistics and confusion tables[8] is displayed in a separate window.
	Verbose output (command line)	Several messages are displayed during the classifier tuning procedure in the command line or in MATLAB's command window if MATLAB is present.

After setting the parameters, the user should click **Tune** and classifier tuning will be performed. Depending on the kNN tuning results, the user can select the appropriate parameters to build a model that best fits the dataset under examination. If the user selects to display classifier evaluation plots by checking the box **Display evaluation plots**, the following examples depict how these plots are presented, according to selected validation methods (1st plot using n-fold cross validation, 2nd leave-m-out and 3rd training and test):

[8] A confusion matrix is a visualization tool typically used in supervised learning. Each column of the matrix represents the instances in a predicted class, while each row represents the instances in an actual class. One benefit of a confusion matrix is that it is easy to see if the classifier is confusing two classes (i.e. commonly mislabelling one as another).

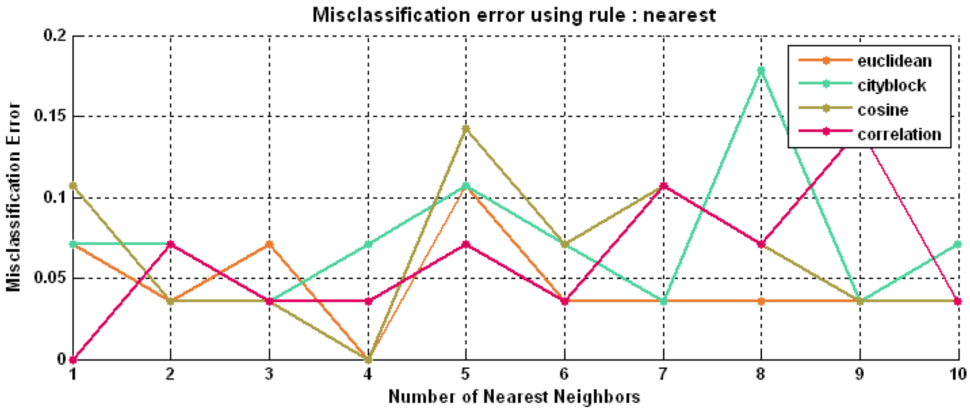
Misclassification error using rule : nearest
Misclassification Error
Number of Nearest Neighbors
euclidean
cityblock
cosine
correlation

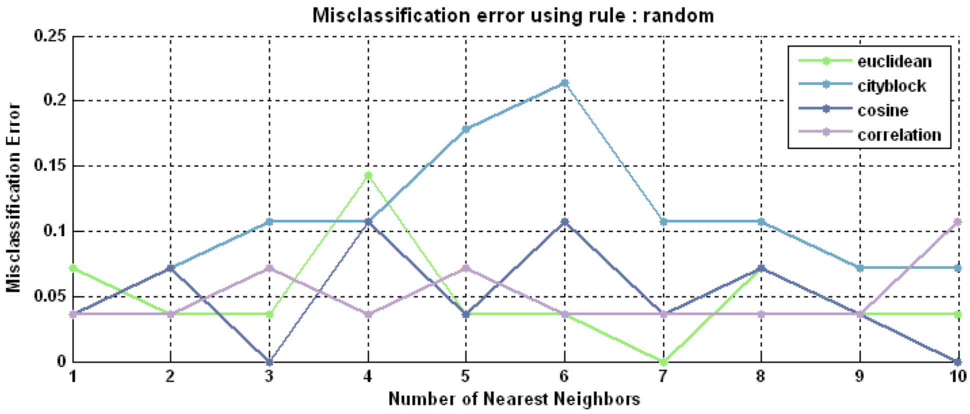
Misclassification error using rule : random
Misclassification Error
Number of Nearest Neighbors
euclidean
cityblock
cosine
correlation

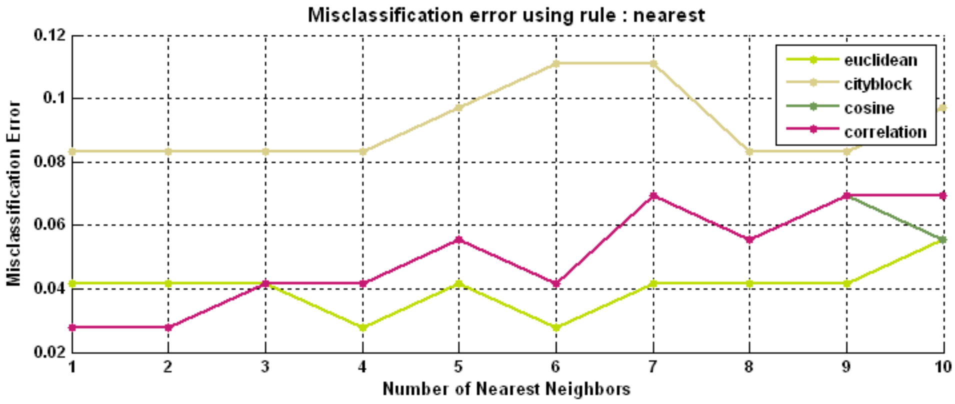
Misclassification error using rule : nearest
Misclassification Error
Number of Nearest Neighbors
euclidean
cityblock
cosine
correlation

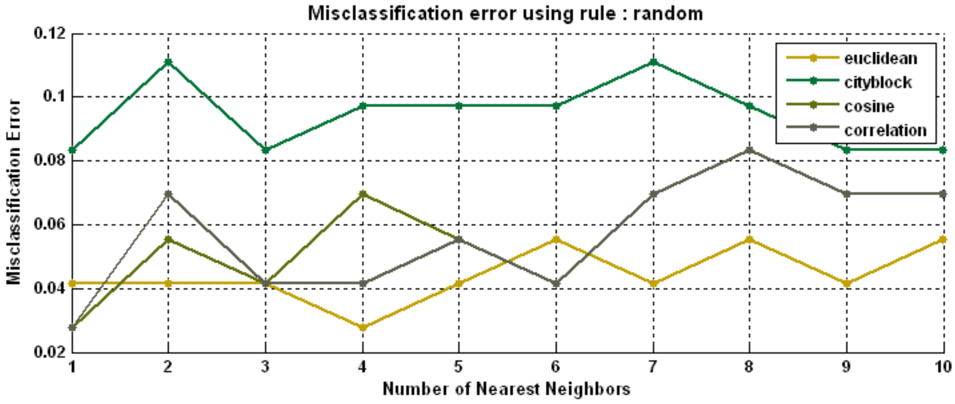
Misclassification error using rule : random
Misclassification Error
Number of Nearest Neighbors
euclidean
cityblock
cosine
correlation

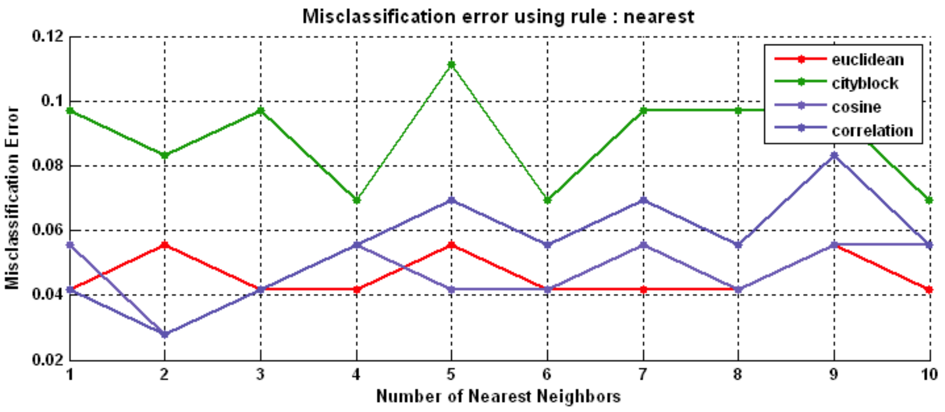

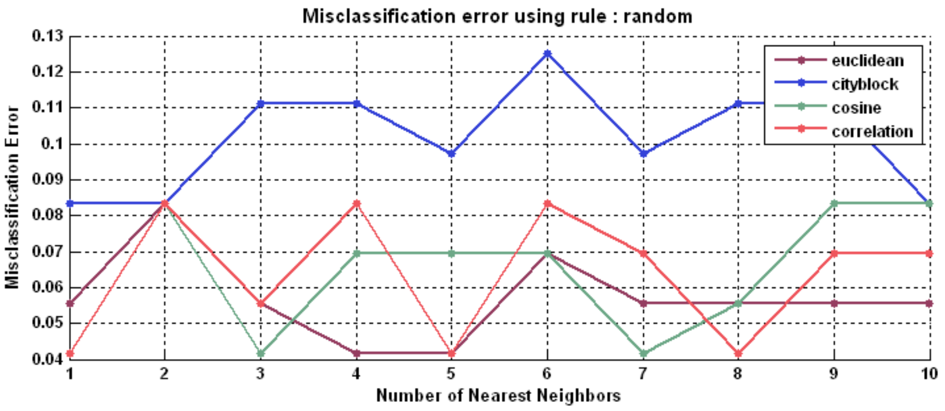

If the user selects to display a classifier evaluation report by clicking **Display output results**, a window like the following will appear presenting the classifier evaluation results:

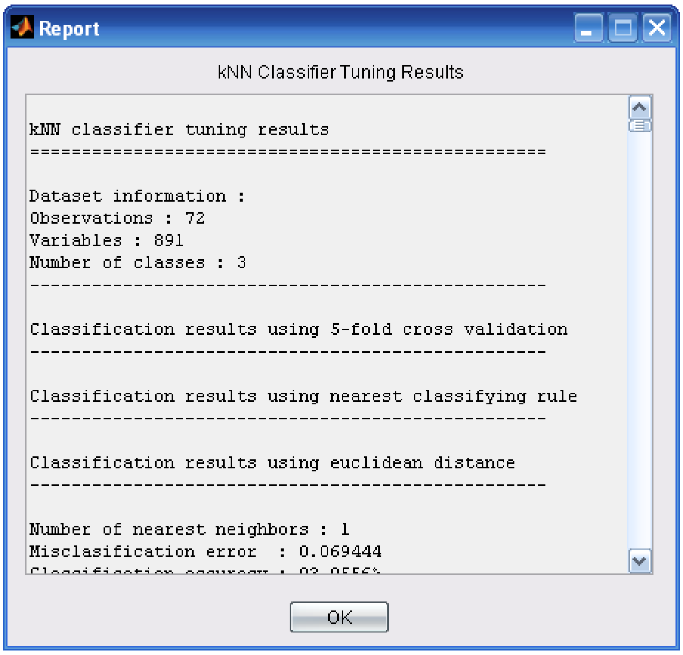

If the user right-clicks inside the report area, a context menu will appear allowing to export the report in a text tab delimited file or clear the report window.

4.4.2.2. k-Nearest Neighbors - Classifying

In order to perform kNN classification, the user should click **Statistics** → **Classification** → **k – Nearest Neighbors** → **Classify** and the following preferences window will appear:

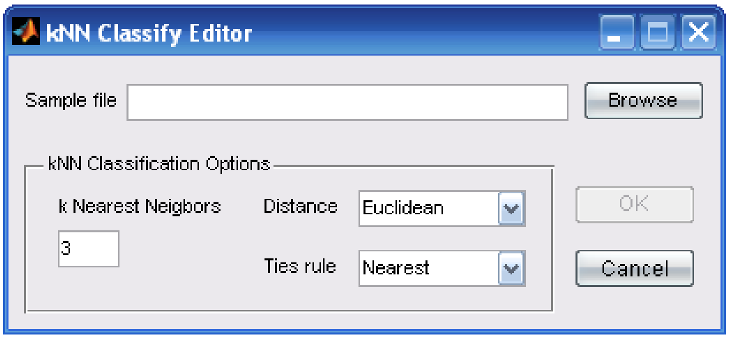

The user should select the file that contains the new samples to be classified using as training data the data imported to ARMADA. The file can be a text tab delimited or Excel file which should be structured as follows: the first column should contain variable names (e.g. gene names) that serve as unique variable identifiers. The first row should contain sample names that will be used to identify the new samples when they will be assigned to classes. The rest of the data should be numeric. Attention should be paid so that the number of variables-features is the same as the number of features used to build the classifier model. For an example of a file of new samples to be classified, the user should consult Appendix A.

The rest of the options in the kNN Classify preferences window under the **kNN classification options** panel (**k Nearest Neighbors**, **Distance** and **Ties rule**) are the same as in the case of tuning the kNN classifier and their description can be found in section 4.4.2.1. After setting the desired parameters, the user should click **OK**. After the classification procedure, a report window will appear:

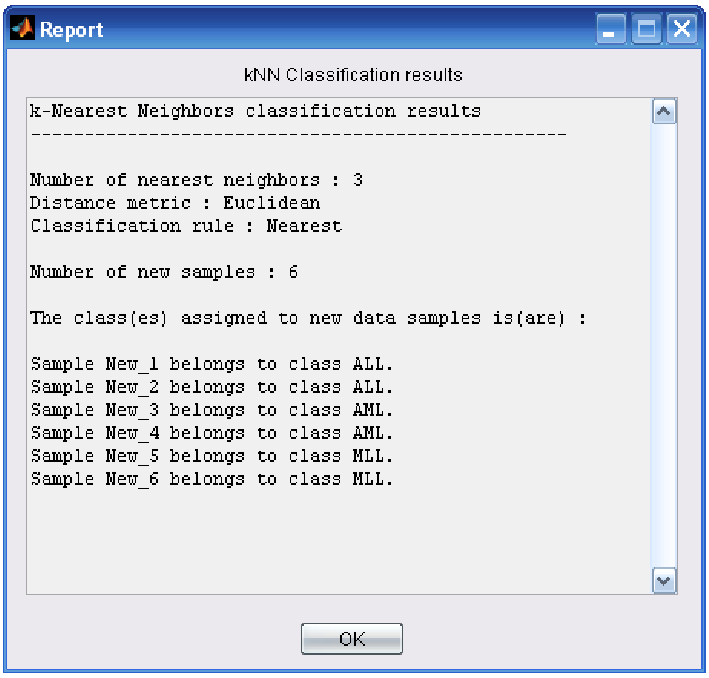

4.4.3. Support Vector Machines

Support Vector Machines (SVM) classification method was developed by Vapnik (14) for binary classification and have been extensively used for microarray data classification (e.g. (15)). Briefly, the optimal separating hyperplane between the two classes is computed by maximizing the margin between the classes' closest points. The points lying on the boundaries are called support vectors and the middle of the margin is the optimal separating hyperplane. The points of the "wrong" side of the discriminant margin are weighted down to reduce their influence. When a linear separator cannot be found, the points are projected into a higher dimensional space where the points effectively become linearly separable. A program able to perform such optimization tasks is called a Support Vector Machine. Although SVMs were initially developed for binary, classification problems, there exist several strategies that can deal with this problem. An example is "one-against-one" approach, in which *k(k-1)/2* binary classifiers are trained; the appropriate class is found by a voting scheme. The SVM classifier implemented in ARMADA is based on the OSU SVM Toolbox (http://sourceforge.net/projects/svm/) which supports mutliclass classification. The following sub-sections describe the tuning and classification process using SVMs in ARMADA.

4.4.3.1. Support Vector Machines – Tuning

In order to perform SVM classifier tuning, the user should select an Analysis object from the Analysis Objects list and click **Statistics** → **Classification** → **Support Vector Machines** → **Tune**. The following preferences window will appear:

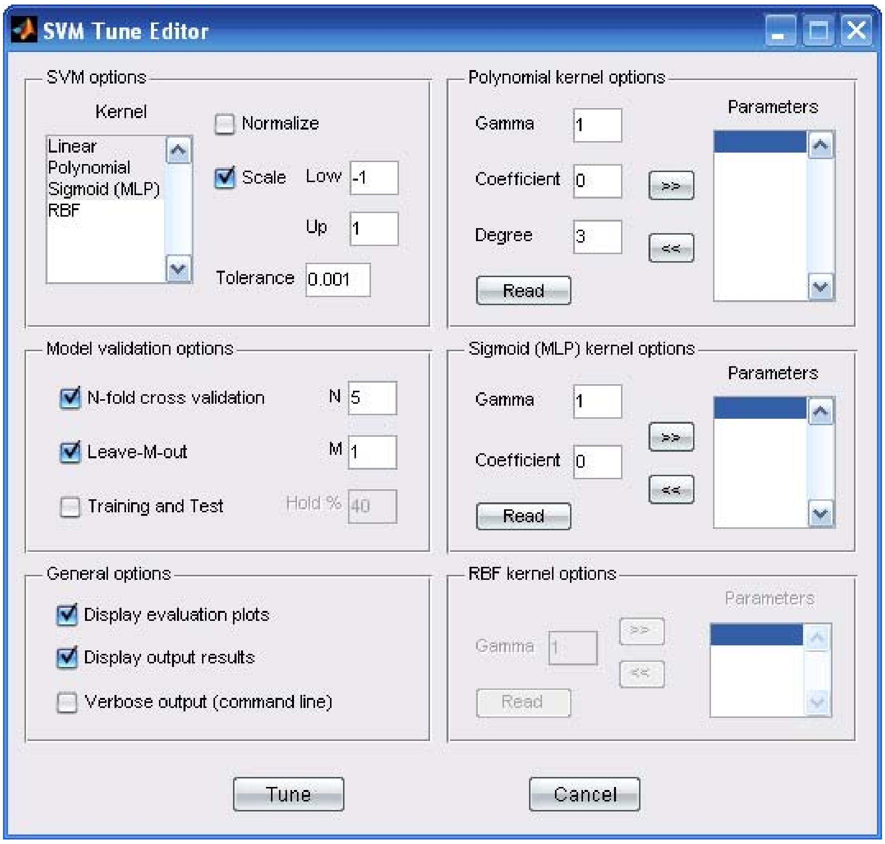

The following table explains the available options in the **SVM options**, **Model validation options**, **General options**, **Polynomial kernel options**, **Sigmoid (MLP) kernel options** and **RBF kernel options** panels:

	**Option**	**Description**
**SVM options**	Kernel	The kernel function type used to build the classifier model. The following kernel types are available (*X* denotes the data matrix):<br>Linear: The kernel function has the form $k(X)=w\cdot X-b=0$<br>Polynomial: The kernel function has the form $k(X,X^T)=\left(Gamma\cdot\langle X,X^T\rangle+Coefficient\right)^{Degree}$<br>Sigmoid (MLP): MLP stands for Multi-Layer Perceptron. The kernel function has the form $k(X,X^T)=tanh\left(Gamma\cdot\langle X,X^T\rangle+Coefficient\right)$<br>RBF: RBF stands for Radial Basis Function. The kernel function has the form $k(X,X^T)=e^{-Gamma\cdot\left\|X-X^T\right\|^2}$
	Normalize	Normalize input data matrix so that each column has mean 0 and standard deviation 1.
	Scale	Scale input data matrix so that all data values lie between a given range. The upper and lower limits can be given through the corresponding text boxes.
	Tolerance	Tolerance of termination criterion.
**Polynomial kernel**	Gamma	The gamma coefficient in the polynomial kernel model.
	Coefficient	The correction coefficient in the polynomial kernel model.
	Degree	The degree of the polynomial kernel model.
	Parameters	Triplets of parameters: they can be entered manually using the respective text boxes or be read by an external file by pressing the **Read** button. The file can be a text tab delimited or Excel file. For details on its format, the user should see Appendix A.
**Sigmoid kernel**	Gamma	The gamma coefficient in the sigmoid kernel model.
	Coefficient	The correction coefficient in the sigmoid kernel model.
	Parameters	Doublets of parameters: they can be entered manually using the respective text boxes or be read by an external file by pressing the **Read** button. The file can be a text tab delimited or Excel file. For details on its format, the user should see Appendix A.
**RBF kernel**	Gamma	The gamma coefficient in the sigmoid kernel model.
	Parameters	Parameter values: they can be entered manually using the respective text box or be read by an external file by pressing the **Read** button. The file can be a text tab delimited or Excel file. For details on its format, the user should see Appendix A.
**Model validation**	N-fold cross validation	The user should check this box to perform N-fold cross validation of the classifier and supply N.
	Leave-M-out	The user should check this box to perform Leave-M-out validation of the classifier and supply M.
	Training and Test	The user should check this box to perform Training and Test validation of the classifier and supply the percentage of the dataset that should be held out for testing.
**General**	Display evaluation plots	Displays classifier evaluation plots based on the tuning options and parameters (plots are based on the number of nearest neighbors, distances, tie-break rules and validation methods).

Display output results	A report containing classification evaluation statistics and confusion tables[9] is displayed in a separate window.
Verbose output (command line)	Several messages are displayed during the classifier tuning procedure in the command line or in MATLAB's command window if MATLAB is present.

After setting the parameters, the user should click **Tune** and classifier tuning will be performed. Depending on the SVM tuning results, the user can select the appropriate parameters to build a model that best fits the dataset under examination. If the user selects to display classifier evaluation plots by checking the box **Display evaluation plots**, the following example depicts how these plots are presented, according to selected validation methods:

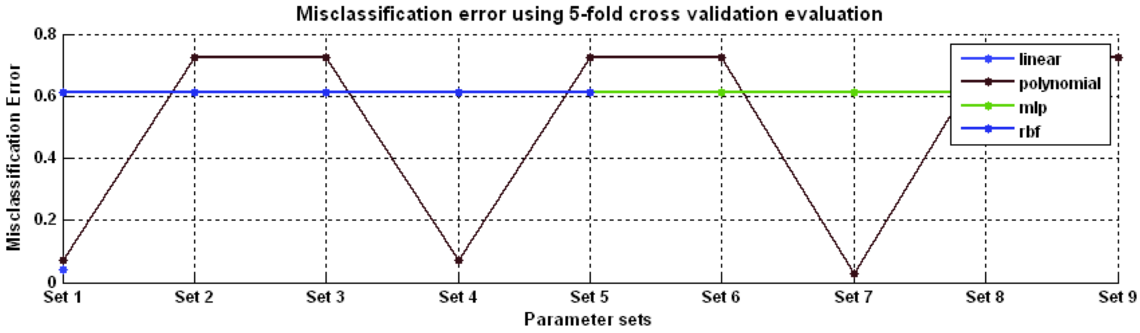

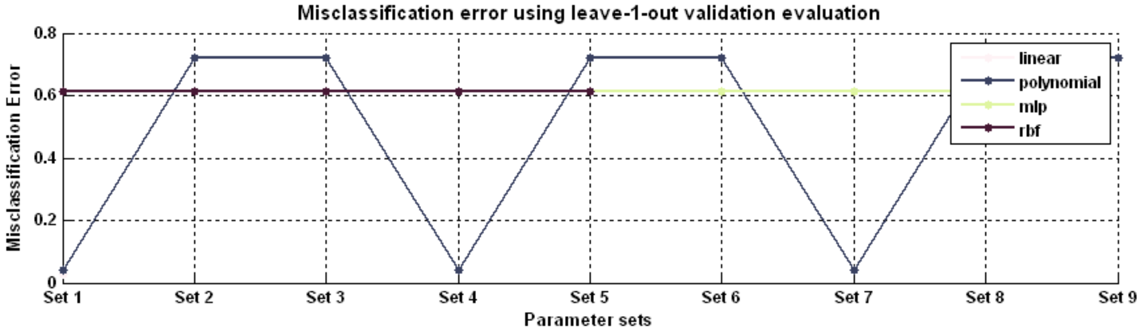

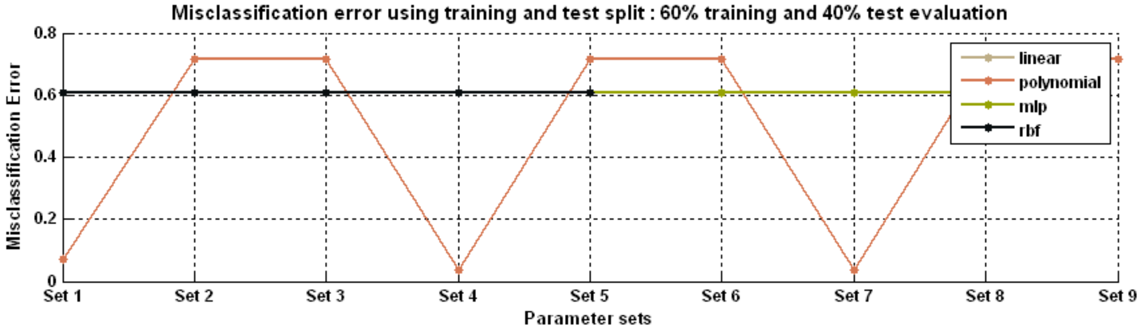

If the user selects to display a classifier evaluation report by clicking **Display output results**, a window like the following will appear presenting the classifier evaluation results:

[9] A confusion matrix is a visualization tool typically used in supervised learning. Each column of the matrix represents the instances in a predicted class, while each row represents the instances in an actual class. One benefit of a confusion matrix is that it is easy to see if the classifier is confusing two classes (i.e. commonly mislabelling one as another).

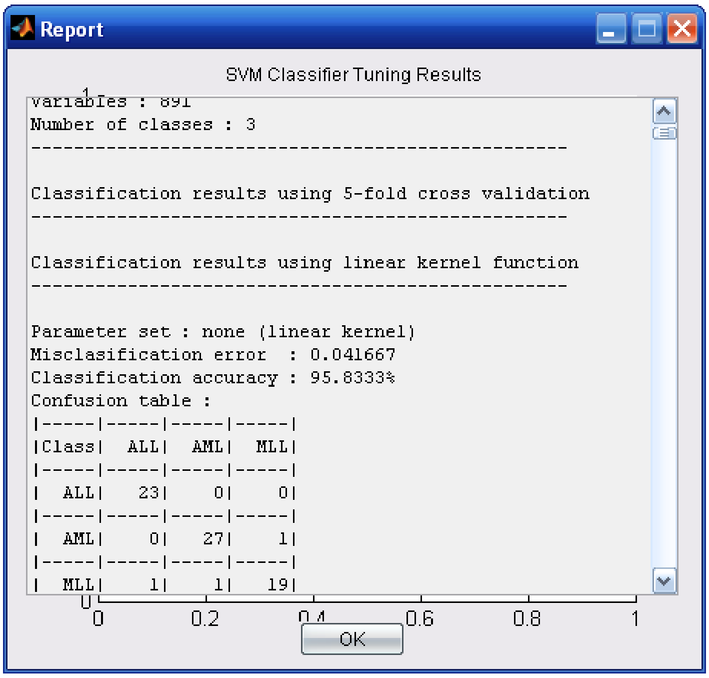

If the user right-clicks inside the report area, a context menu will appear allowing to export the report in a text tab delimited file or clear the report window.

4.4.3.2. Support Vector Machines - Training

In order to train an appropriate SVM classifier model (normally after a tuning session so as to evaluate the best kernel and respective parameters for the dataset under examination), the user should click **Statistics** → **Classification** → **Support Vector Machines** → **Train** and the following preferences window will appear:

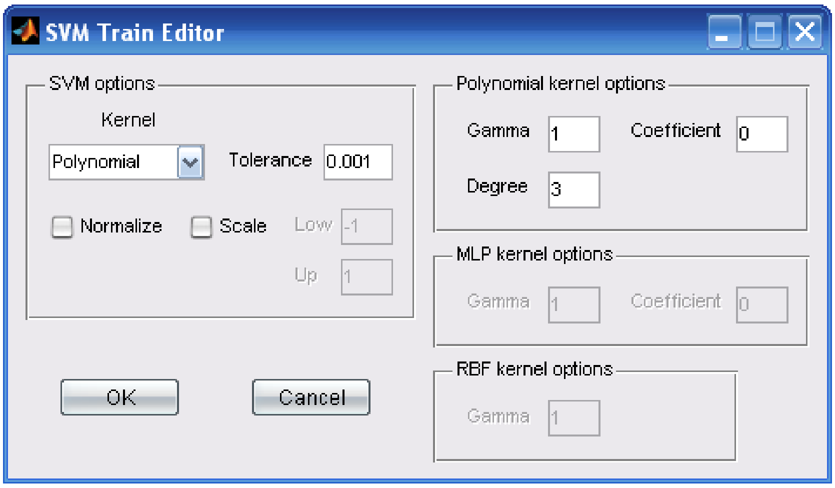

The options in the SVM training preferences window under the **SVM options** panel (**Kernel**, **Normalize**, **Scale** and **Tolerance**) are the same as in the case of tuning the SVM classifier and their description can be found in section 4.4.3.1. After setting the desired parameters, the user should click **OK**. After the classification procedure, a confirmation dialog will appear stating that the classifier training has finished. ARMADA will store the classifier model for further use.

4.4.3.3. Support Vector Machines - Classifying

In order to perform SVM classification model the user should click **Statistics** → **Classification** → **Support Vector Machines** → **Classify** and the following window will appear, prompting the user to select the file that contains the new samples:

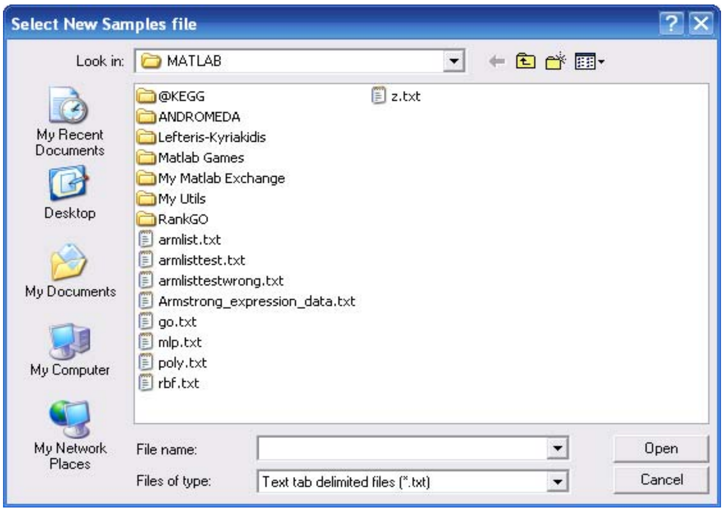

The file can be a text tab delimited or Excel file which should be structured as follows: the first column should contain variable names (e.g. gene names) that serve as unique variable identifiers. The first row should contain sample names that will be used to identify the new samples when they will be assigned to classes. The rest of the data should be numeric. Attention should be paid so that the number of variables-features is the same as the number of features used to build the classifier model. The classifier model that will be used is the one corresponding to the Analysis Object highlighted in the Analysis Object list. Example of a file of new samples to be classified can be found in Appendix A. After the classification process, a report window will appear:

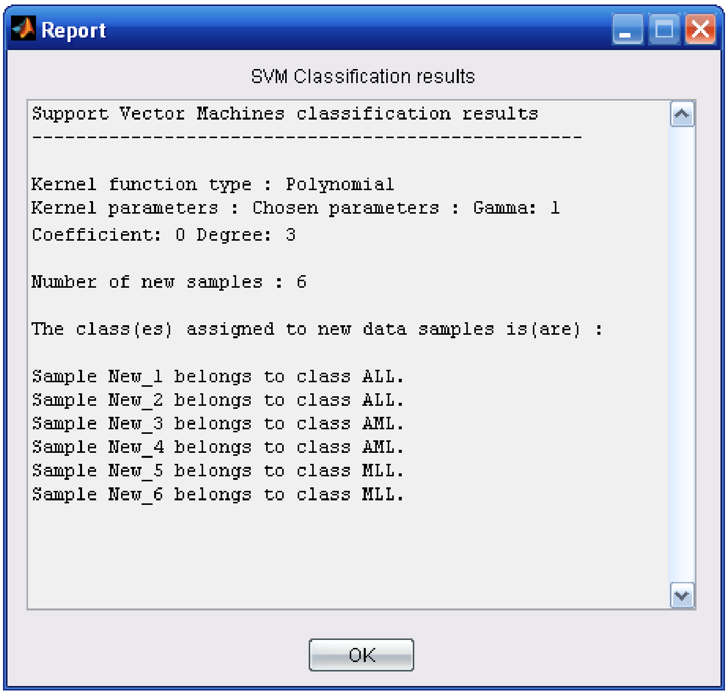

5. Graphical data exploration

The following sections describe several graphics which are customizable and accessible in ARMADA through the **Plots** menu in the main window. Such graphics include 2 or 3 dimensional array reconstructed images based on given data, MA plots, expression distribution plots across different arrays, boxplots, volcano plots and expression profiles across different arrays or conditions. The user should note that not all plots are available at any time of the analysis. For example MA plots become available after normalization and volcano plots become available after the statistical selection procedure and only if the selected Analysis has two conditions.

5.1. Array Images

An array image depicts a reconstructed spatial image of a microarray based on the data of the corresponding input file(s). The image can be created using several input data (e.g. the user can create an image based on Channel 1 mean signal or Channel 2 background median). Such images can help the user identify several spatial hybridization effects or the presence of artifacts on arrays responsible for high background contamination and perform quality control. To create array images, the user should select an array from the Arrays list and then click **Plots** → **Array Images**. The following window appears:

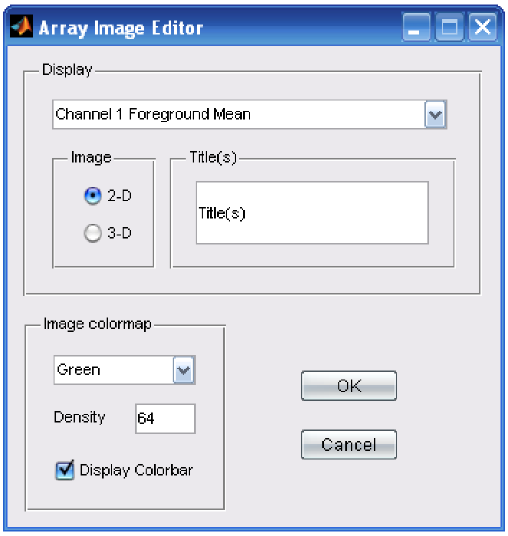

From there, the user is able to select the type of available data to be displayed on the reconstructed image, the image dimensionality to be displayed (2D or 3D), the color settings (**Image colormap**) as well as the color density (e.g. a density of 64 will create 64 intermediate colors between the basic colors that defined the colormap while a density of 256 will create 256 intermediate variations) and whether to display a bar depicting the color-data correspondence for the data range that was used to create the image. The following picture depicts the supported colormaps (taken from MATLAB's help) apart from Red and Green colormaps which are created by ARMADA. The default colormap is the 'Jet' colormap.

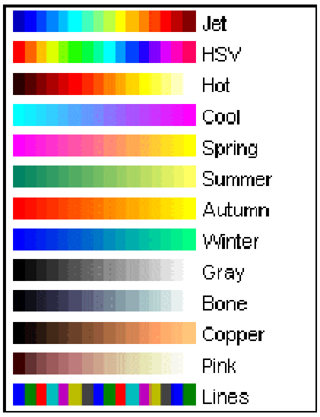

The following table presents the data types, derived from the imported files, that the user can use to create array images (choices may vary depending on the input data file type and the available data in the case of importing text tab delimited files):

**Data type**	**Description**
Channel 1 Foreground Mean	The foreground spot signal mean for channel 1 (or 'Cy3' or 'Green').
Channel 2 Foreground Mean	The foreground spot signal mean for channel 2 (or 'Cy5' or 'Red').
Channel 1 Foreground Median	The foreground spot signal median (if available) for channel 1 (or 'Cy3' or 'Green').
Channel 2 Foreground Median	The foreground spot signal median (if available) for channel 2 (or 'Cy5' or 'Red').
Channel 1 Background Mean	The background noise spot mean for channel 1 (or 'Cy3' or 'Green').
Channel 2 Background Mean	The background noise spot mean for channel 2 (or 'Cy5' or 'Red').
Channel 1 Background Median	The background noise spot median (if available) for channel 1 (or 'Cy3' or 'Green').
Channel 2 Background Median	The background noise spot median (if available) for channel 2 (or 'Cy5' or 'Red').
Channel 1 Foreground Standard Deviation	The foreground spot signal standard deviation (if available) for channel 1 (or 'Cy3' or 'Green').
Channel 2 Foreground Standard Deviation	The foreground spot signal standard deviation (if available) for channel 2 (or 'Cy5' or 'Red').
Channel 1 Background Standard Deviation	The background noise spot standard deviation (if available) for channel 1 (or 'Cy3' or 'Green').
Channel 2 Background Standard Deviation	The background noise spot standard deviation (if available) for channel 2 (or 'Cy5' or 'Red').
Channel 1 Foreground - Background (Mean)	The difference between mean signal and background noise for channel 1 (or 'Cy3' or 'Green').
Channel 2 Foreground - Background (Mean)	The difference between mean signal and background noise for channel 2 (or 'Cy5' or 'Red').
Channel 1 Foreground - Background (Median)	The difference between the medians (if available) of signal and background noise for channel 1 (or 'Cy3' or 'Green').
Channel 2 Foreground - Background (Median)	The difference between the medians (if available) of signal and background noise for channel 2 (or

	‘Cy5’ or ‘Red’).
Channel 1 Foreground / Background (Mean)	The signal-to-noise ratio between mean signal and background noise for channel 1 (or ‘Cy3’ or ‘Green’).
Channel 2 Foreground / Background (Mean)	The signal-to-noise ratio between mean signal and background noise for channel 2 (or ‘Cy5’ or ‘Red’).
Channel 1 Foreground / Background (Median)	The signal-to-noise ratio between the medians (if available) of signal and background noise for channel 1 (or ‘Cy3’ or ‘Green’).
Channel 2 Foreground / Background (Median)	The signal-to-noise ratio between the medians (if available) of signal and background noise for channel 2 (or ‘Cy5’ or ‘Red’).

The user is also able to provide titles for the images to be created. Titles are given in the **Title(s)** panel and should be as many as the arrays selected from the Arrays list on the main window. Different titles should be separated by a new line (Enter). The field should remain empty for automatic title generation. After specifying the desired parameters the user should click **OK**. Below, there are some examples of 2 and 3 dimensional array images created with different colormaps:

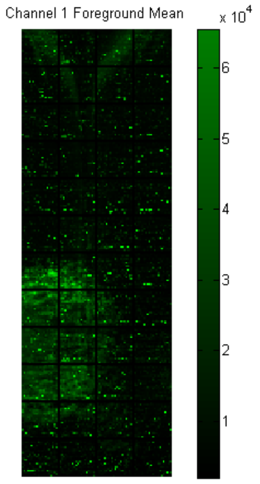

2D Image of Channel 1 Foreground Mean for array Wt_1r.txt
Colormap : Green

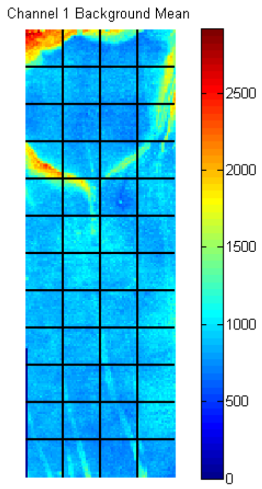

2D Image of Channel 1 Background Mean for array d15_1r.txt
Colormap : Jet

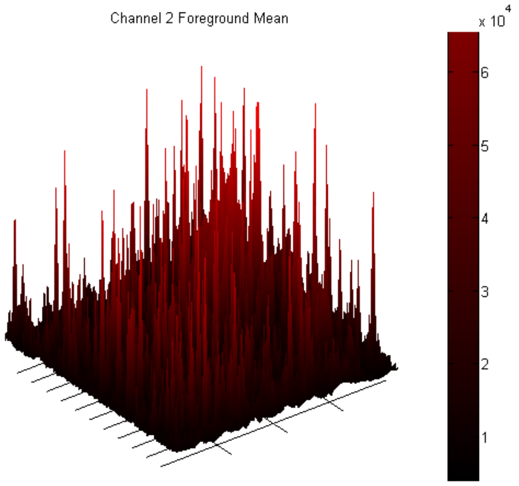

3D Image of Channel 2 Foreground Mean for array d23_1r.txt
Colormap : Red

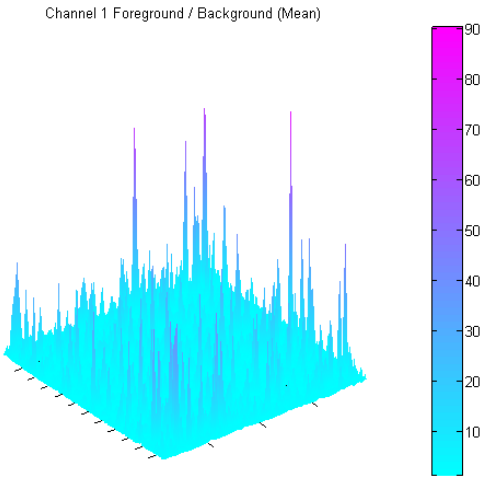

3D Image of Channel 1 Foreground/Background (Mean) for array d23_1r.txt
Colormap : Cool

If the user clicks on any of the array images created, individual spot data are displayed (as in **Raw Image** in ARMADA's main window). The user should also note that array spatial images are available only if grid coordinates and meta-coordinates are provided with the input files and that if meta-coordinates exist, the array images are available right after importing the data files to ARMADA.

5.2. Normalized and Un-normalized images

An array normalized or un-normalized image depicts an array spatial image reconstructed using the normalized or un-normalized $\log_2$ ratio between the two channels. Such images may help the user identify individual characteristics of microarrays, identify possible differentially expressed transcripts and check the effects of normalization procedures as well as compare normalized versus un-normalized images. To create (un)normalized array images, the user should select an Analysis from the Analysis Object list and click **Plots → (Un)Normalized Images**. The following window appears:

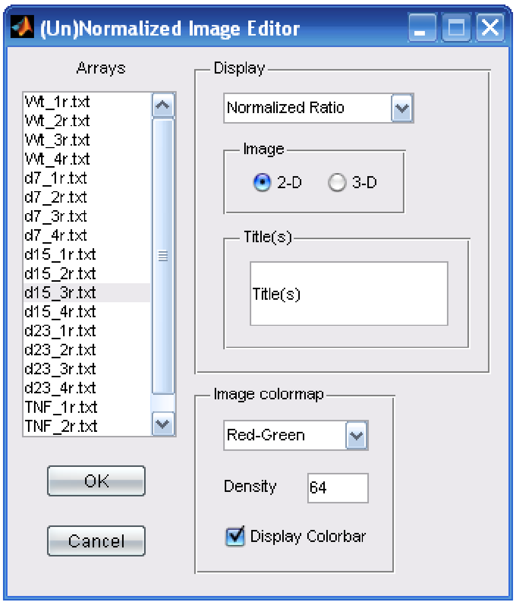

In the **Arrays** list, the (un)normalized images preferences window displays the arrays from the currently selected Analysis in the Analysis Object list. The user can select one or multiple arrays for image creation and, as with the array images described in section 5.1, there are options on what data to use for image creation, the image color settings and the colormap density as well as whether to display a colorbar or not. As before, the user may supply own titles, or leave the filed **Title(s)** empty for automatic figure title generation. The following table presents the data types the user can use to create array images:

**Data type**	**Description**
Normalized Ratio	Between channels $\log_2$ ratio as calculated after data normalization.
Unnormalized Ratio	Between channels $\log_2$ ratio as calculated prior to data normalization.

After finishing with setting the desired parameters, the user should click **OK** in order to create the images. Below, there are two examples of 2 and 3 dimensional array normalized or un-normalized images created with different colormaps:

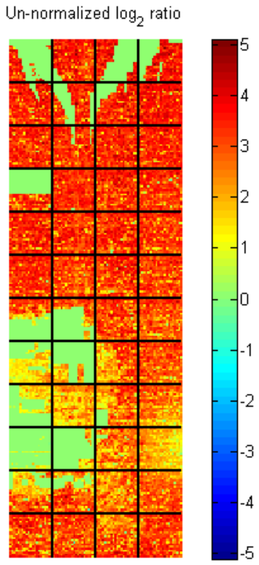

2D Image of Un-normalized ratio
for array Wt_1r.txt
Colormap : Jet

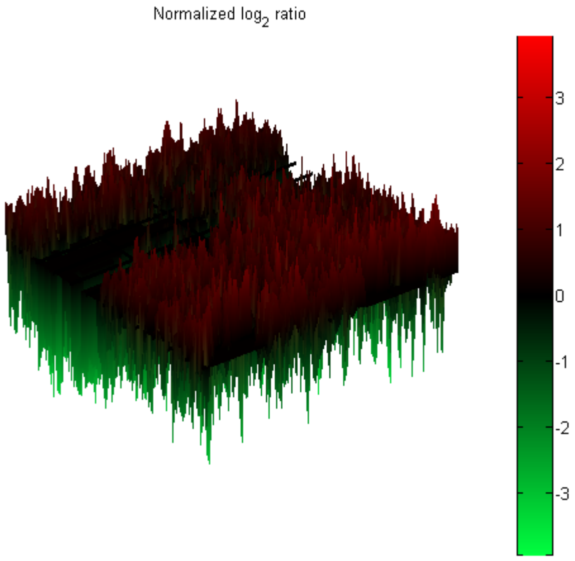

3D Image of Normalized ratio for array Wt_1r.txt
Colormap : Red-Green

If the user clicks on any of the images created, individual spot data are displayed (as in **Normalized Image** in ARMADA's main window). The user should also note that (un)normalized spatial images are available only if grid coordinates and meta-coordinates are provided with the input files and that if meta-coordinates exist, the (un)normalized images are available right after the data normalization step (the user should see also 3.4).

At this point it should be noted that for better image exploration, as well as image saving, exporting and other figure operations, the user can utilize several figure controls and utilities which are provided by MATLAB's interface and are briefly explained in Appendix B.

5.3. Array plots

An array plot can depict the comparison of several input data (e.g. Channel 1 mean signal vs Channel 2 mean signal) based on the input files or can depict the comparison between different arrays for the same measurements as well as $\log_2$ ratios or intensities if normalization has been performed. Such images can help the user identify several phenomena connected to the nature of the experiment or identify correlations or differences between different dyes or different arrays of the same or another experimental condition. To create array plots, the user should click **Plots → Array Plots** and the following window will appear:

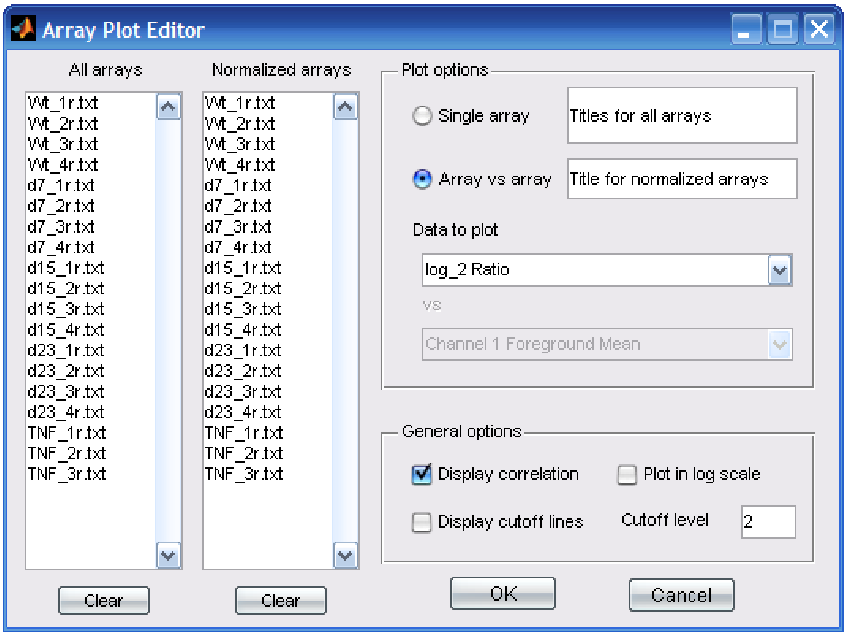

In the **All arrays** list, all the imported arrays of the experiment are displayed and the user can select which arrays to plot. On the other hand, the **Normalized arrays** list displays the normalized arrays which correspond to the conditions of the analysis highlighted in the Analysis Object list on ARMADA's main window. In the **Plot options** panel, when the **Single array** choice is selected, the **Normalized arrays** list and the bottom drop down list are deactivated and reactivated when the **Array vs array** choice is selected. When the **Single array** choice is selected, the user can choose which measurements to plot in a 2-D plot for each array by selecting from the drop down lists **Data to plot**. Additionally, the letters **H** and **V** which appear next to the lists when **Single array** is selected, represent the **H**orizontal and **V**erical axis respectively. The user can also provide titles for the plots or leave the corresponding fields empty for automatic title generation. Concerning the measurements which are available for plotting, the user should see section 5.1. The **Clear** buttons below the array lists clear the selections allowing the user to make new ones.

The General options panel allows the user to display the Pearson correlation coefficient between the two measurements selected in the 2-D plot as well as plotting in log2 scale by checking the **Display correlation** or **Plot in log scale** boxes respectively. The user can also check the **Display cutoff lines** box. If selected, the resulting plots will also depict the line which crosses the beginning of the axis system ($y=x$) and two lines parallel to it, at a distance chosen by the **Cutoff level** number n ($y=x-n$, $y=x+n$ respectively). After making the necessary selections, the user should click **OK**. Below, there are two examples of array plots. The data on the plots are viewable, selectable and exportable through the use of a right-click context menu. For further details on these operations the user should consult section 5.4.

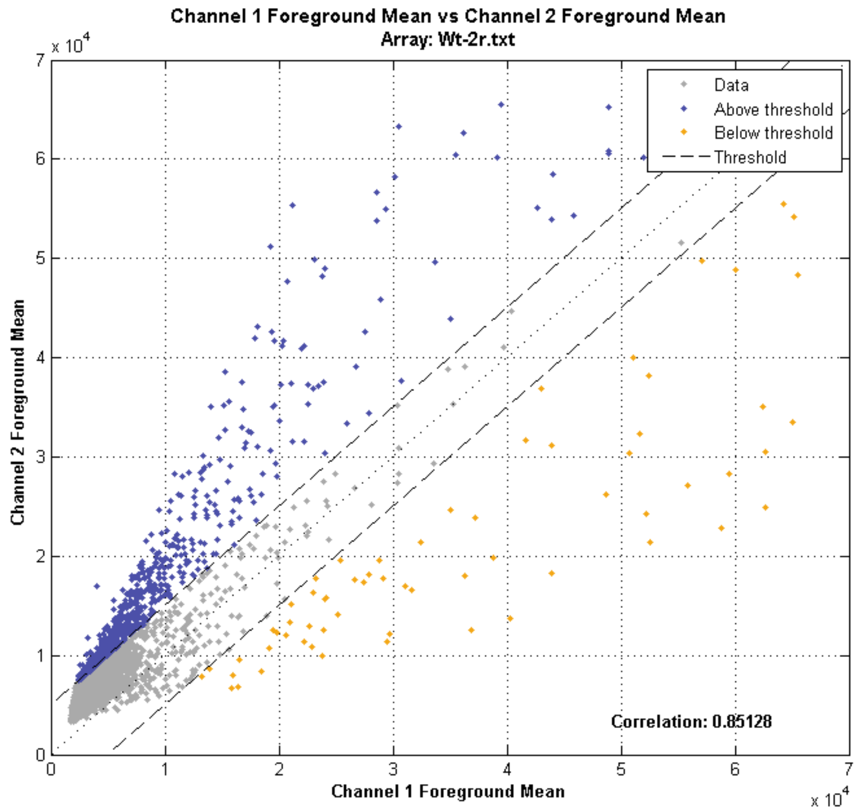

Channel 1 Foreground Mean vs Channel 2 Foreground Mean
Array: Wt-2r.txt
x 10^4
Data
Above threshold
Below threshold
Threshold
Correlation: 0.85128
Channel 2 Foreground Mean
Channel 1 Foreground Mean

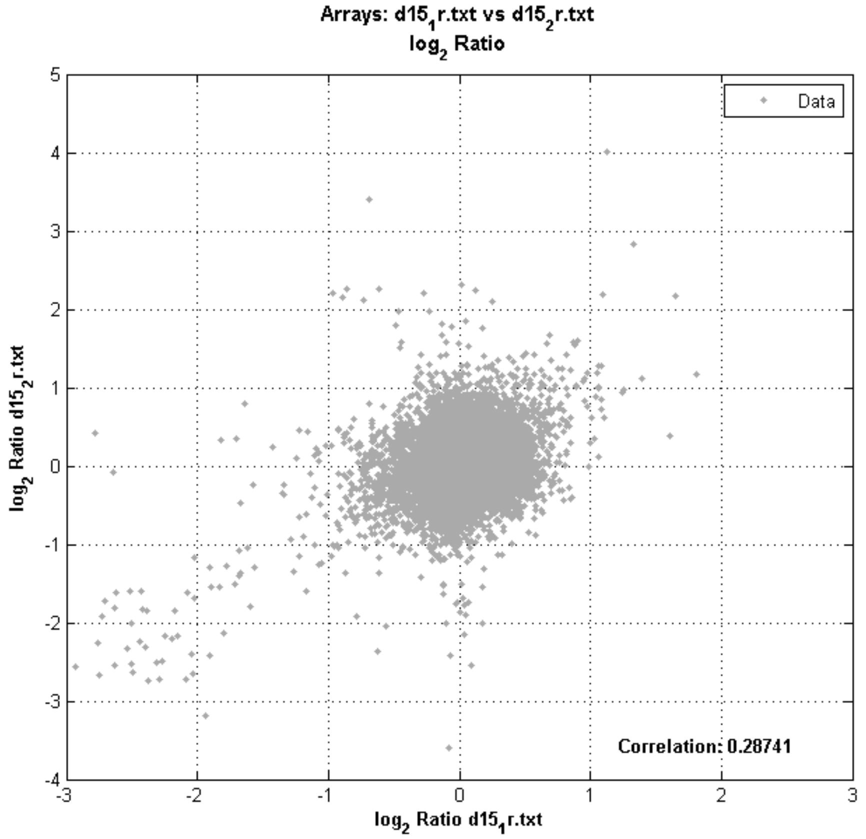

Arrays: d15_1r.txt vs d15_2r.txt
log_2 Ratio
Data
Correlation: 0.28741
log_2 Ratio d15_2r.txt
log_2 Ratio d15_1r.txt

5.4. MA Plots

MA plots of microarray data (7) are plots of log_2 ratio of two channel intensities versus the mean log_2 expression of the two. MA plots are applied to the red ('Channel 2' or 'Cy5') and green ('Channel 1' or Cy3) channels and are representations of the data from single arrays which can be useful in depicting the effects of various normalization methods and quality control issues. To create MA plots, the user should select an Analysis from the Analysis Object list and click **Plots** → **MA Plots**. The following window will appear:

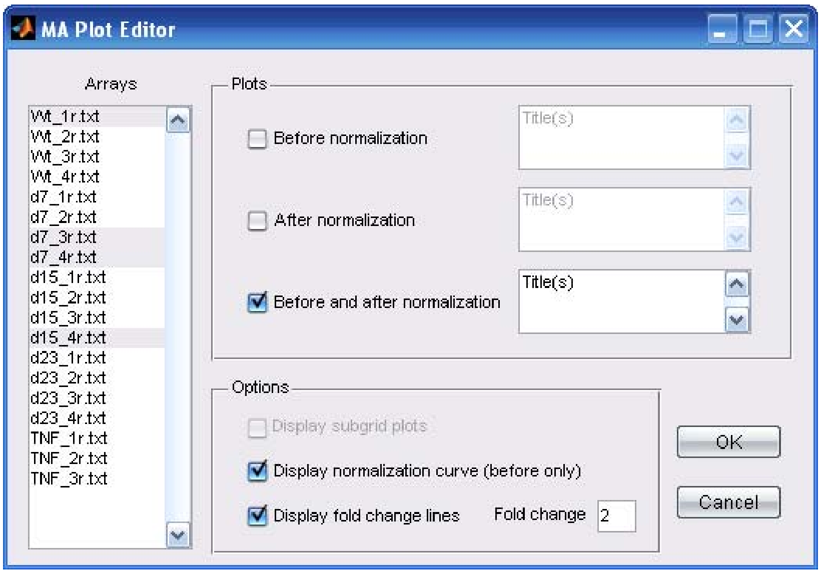

In the **Arrays** list, the user can select one or multiple arrays for which to display MA plots. In the **Plots** panel, the user can select to display MA plots before the normalization procedure, after the normalization procedure or both and also supply the respective titles (one for each selected array, separated by new line (Enter)) if desired. Leaving the title(s) boxes as is or empty will cause automatic plot title generation.

In the **Options** panel, the user may choose to display the above selected plots for each part of the array subgrid (this option is enabled only when subgrid normalization has been performed, the user should see 3.4), choose whether to also display the normalization curve calculated by any of the normalization methods described in section 3.4 (this option applies only to MA plots presenting data before normalization) and finally, choose whether or not to display a fold change line depicting desired thresholds in fold changes among channels. The threshold should be filled in the field **Fold change**. Attention should be paid that while the users fill the fold change in natural scale, the fold change lines are presented in the figures in log_2 scale (e.g. for a fold change of 2, the corresponding threshold lines are at 1 and -1 because $log_2(2)=1$). After setting the parameters, the user should click **OK** for creating the plots. It should be noted here that **MA Plots** in **Plots** menu is activated only after the normalization procedure. The next three subsections describe several figure functionalities provided for each of the three categories of MA plots (before normalization, after normalization and before/after normalization).

5.4.1. MA plots before normalization

Below there is an example of an MA plot before normalization for a specific array:

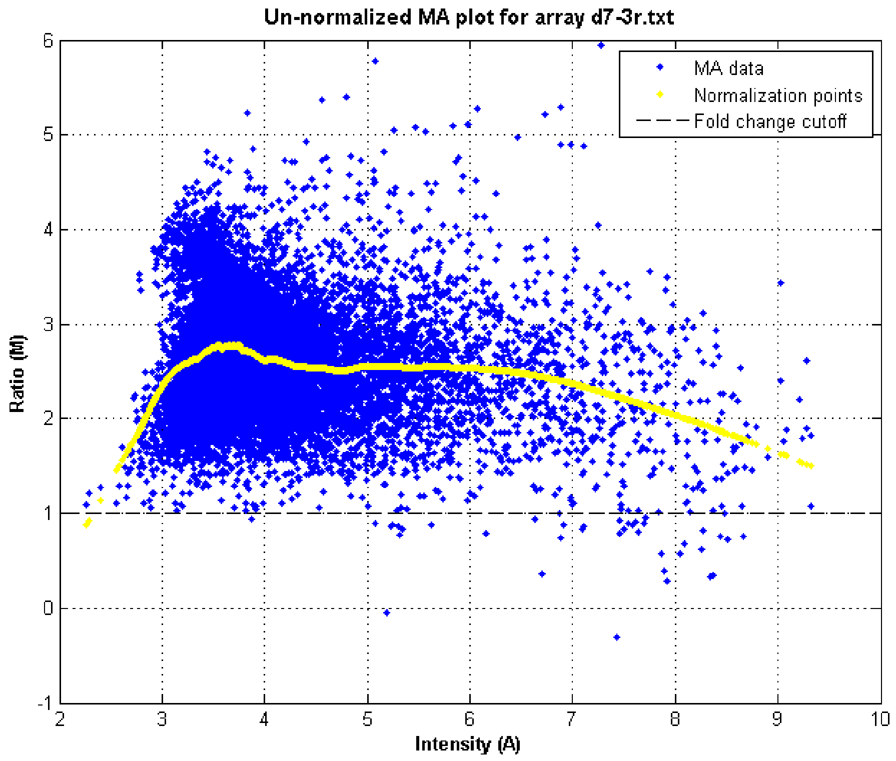

The image created right after clicking **OK** in the MA plots preferences window, is on data exploration mode. In this mode, the user can click on any data point on the figure and then more specific information will be displayed for that point (the GeneID, ratio value etc.). If the user right-clicks inside the figure, the following menu will appear:

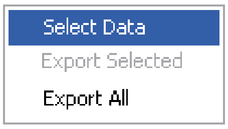

If the user clicks on **Select Data**, the menu title will be check marked and the figure will enter in data selection mode (the cursor will become a cross, +). In this mode, the user can select several data points (genes) by setting a rectangular area which includes the desired data points with the help of the crosshair cursor. To export the selected data points in text tab delimited format, the user should right-click on the *edge* of the specified rectangular area and select **Export Selected**. A new window will appear prompting the user to select a location to place the new file that will contain information (GeneIDs and expression values) on the selected data points. Below there is an example of how the figure looks like when data selection mode is on:

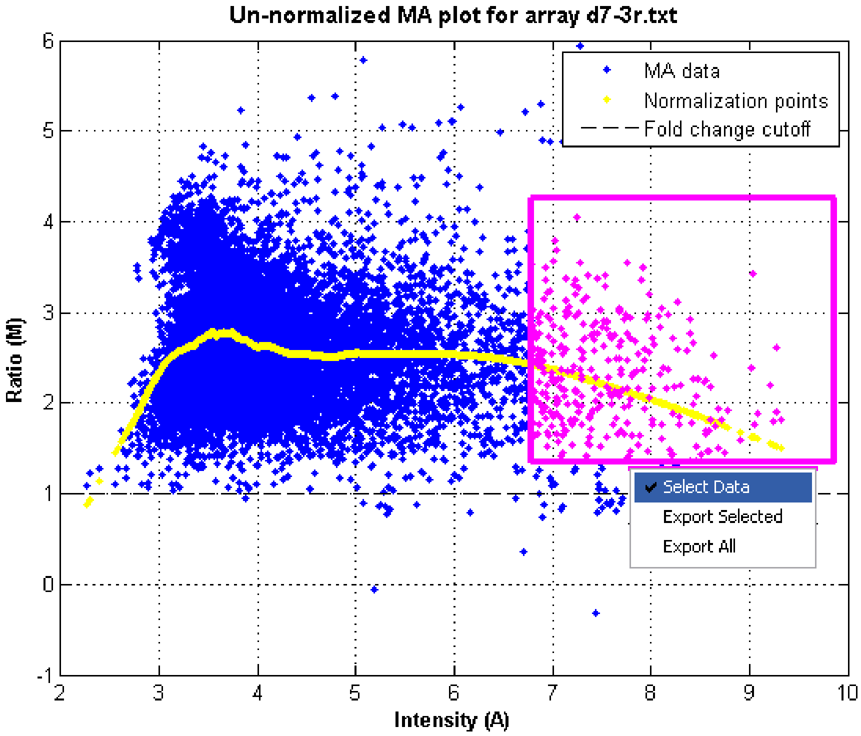

The following table explains the functions of the items displayed in the menu appearing after right-clicking:

Name	Function
Select Data	Switches between data exploration and data selection modes.
Export Selected	While on selection mode, exports data points defined by the rectangular selection area. The user must right-click on one of the *edges* of the selection area.
Export All	Exports all data points, regardless of mode status.

5.4.2. MA plots after normalization

The same things concerning image modes, apply also in the case of MA plots after the normalization procedure with the only difference that when the user right-clicks inside the image the appearing menu is different:

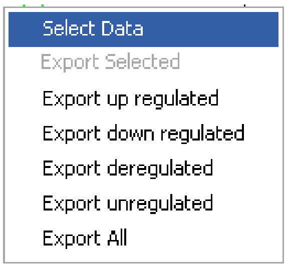

Below there is an example of an MA plot after normalization for a specific array:

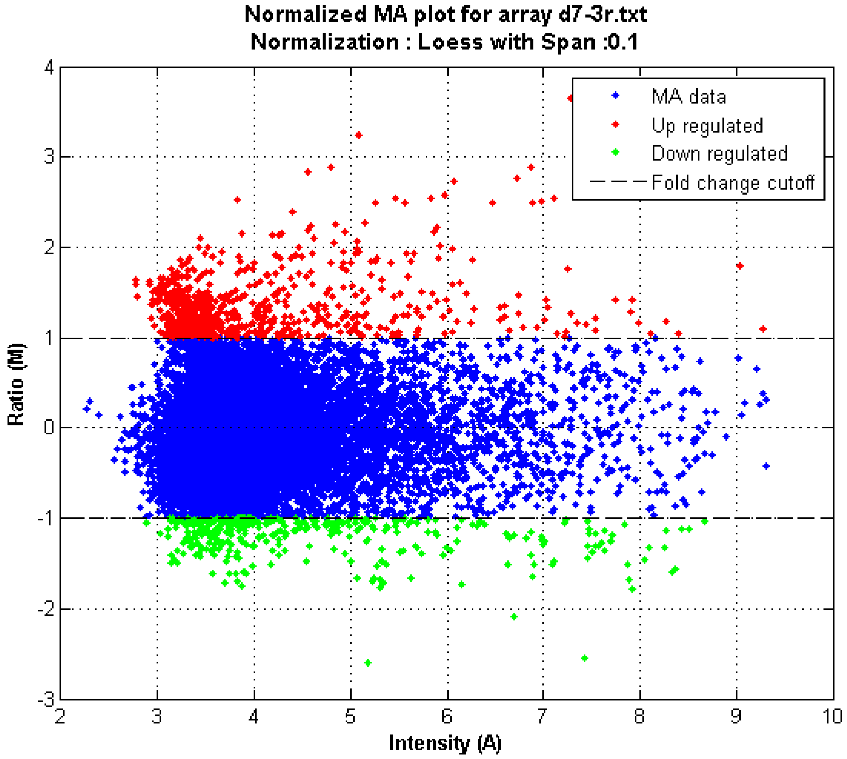

The user should note that red and green points will appear only if the **Display fold change lines** option in the MA plots preferences window has been enabled and a proper fold change threshold value has been provided. The following table explains the functions of the items displayed in the menu appearing after right-clicking:

**Name**	**Function**
Select Data	Switches between data exploration and data selection modes.
Export Selected	While on selection mode, exports data points defined by the rectangular selection area. The user must right-click on one of the *edges* of the selection area.
Export up regulated	Exports up regulated genes (red data points). Available only if fold change thresholds have been provided.
Export down regulated	Exports down regulated genes (green data points). Available only if fold change thresholds have been provided.
Export deregulated	Exports up and down regulated genes (red and green data points). Available only if fold change thresholds have been provided.
Export unregulated	Exports up unregulated genes (blue points). If fold change thresholds have not been provided, exports all data points.
Export All	Exports all data points, regardless of mode status.

5.4.3. MA plots before and after normalization

The same things concerning image modes, apply also in the case of MA plots before and after the normalization procedure. In this case, the output figure consists of two panels: the upper panel contains the MA plot for un-normalized data while the bottom panel contains the MA plot for normalized data. If the user right clicks inside one of the two panels, the menus that appear are the

same as the ones in the cases of MA plots before normalization (5.3.1) and MA plots after normalization (5.3.2). Below there is an example of an MA plots before and after normalization for a specific array:

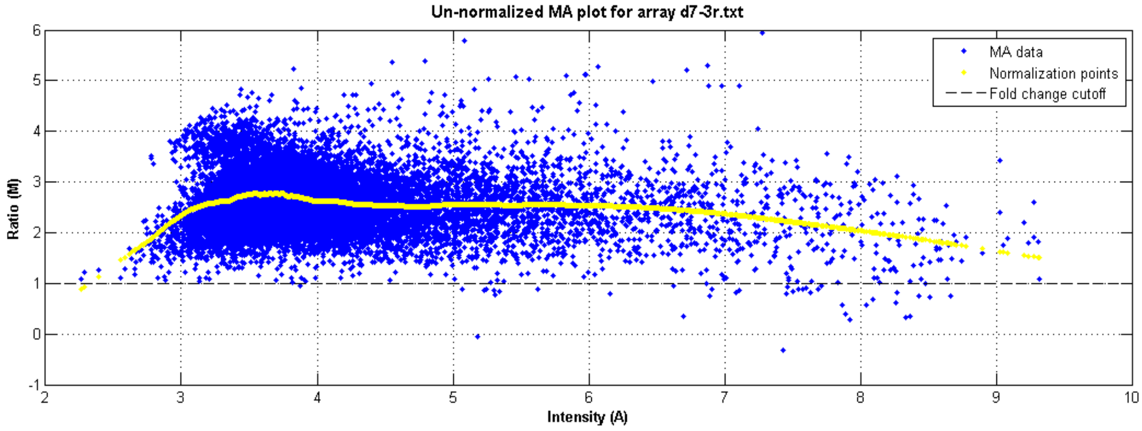

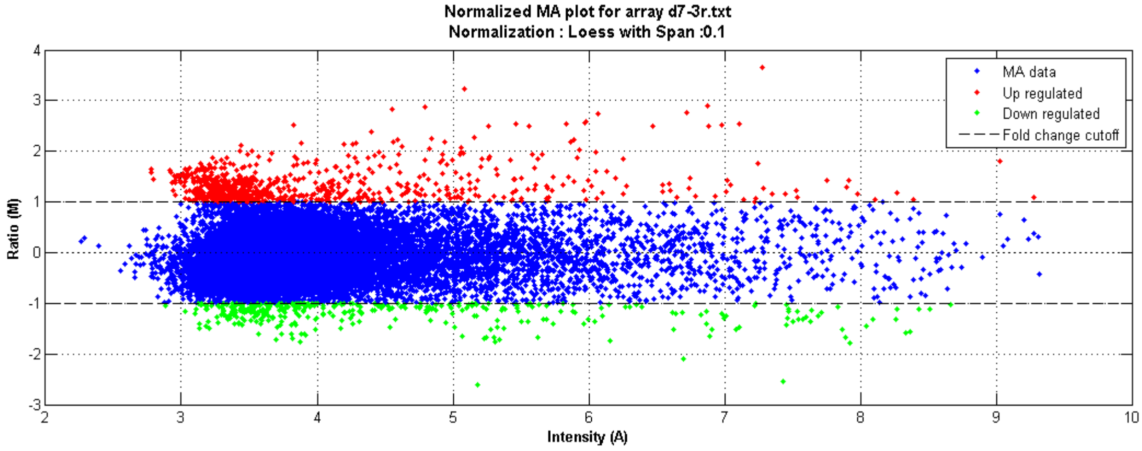

5.4.4. Subgrid MA plots

If subgrid normalization is performed at the presence of array meta-coordinates (the user should see 3.4), MA plots for each subgrid block are also possible (by checking **Display subgrid plots** in the MA plots preferences window). However, the functionalities of simple MA plots (such as data selection and exporting) are not available in the cases of subgrid MA plots. Subgrid MA plots consist of an image with as many blocks as the number of blocks in each slide. Below, there are two examples of subgrid MA plots: the first picture depicts a subgrid MA plot before data normalization while the second depicts a subgrid MA plots after data normalization.

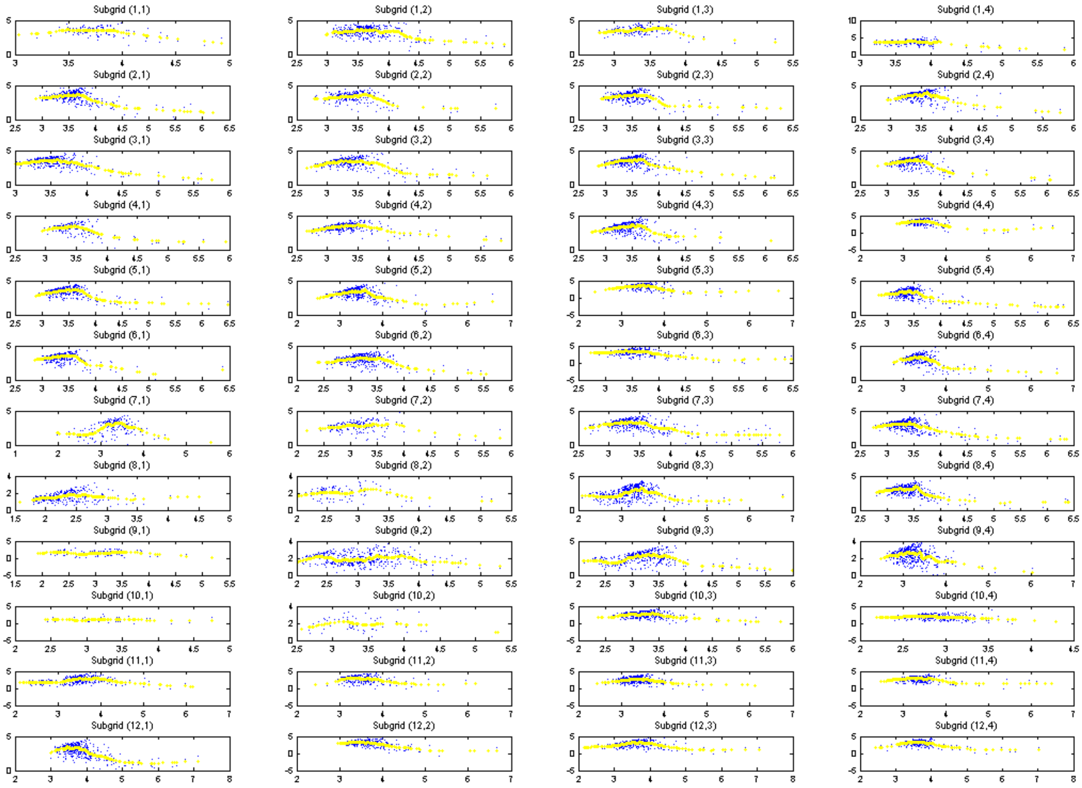

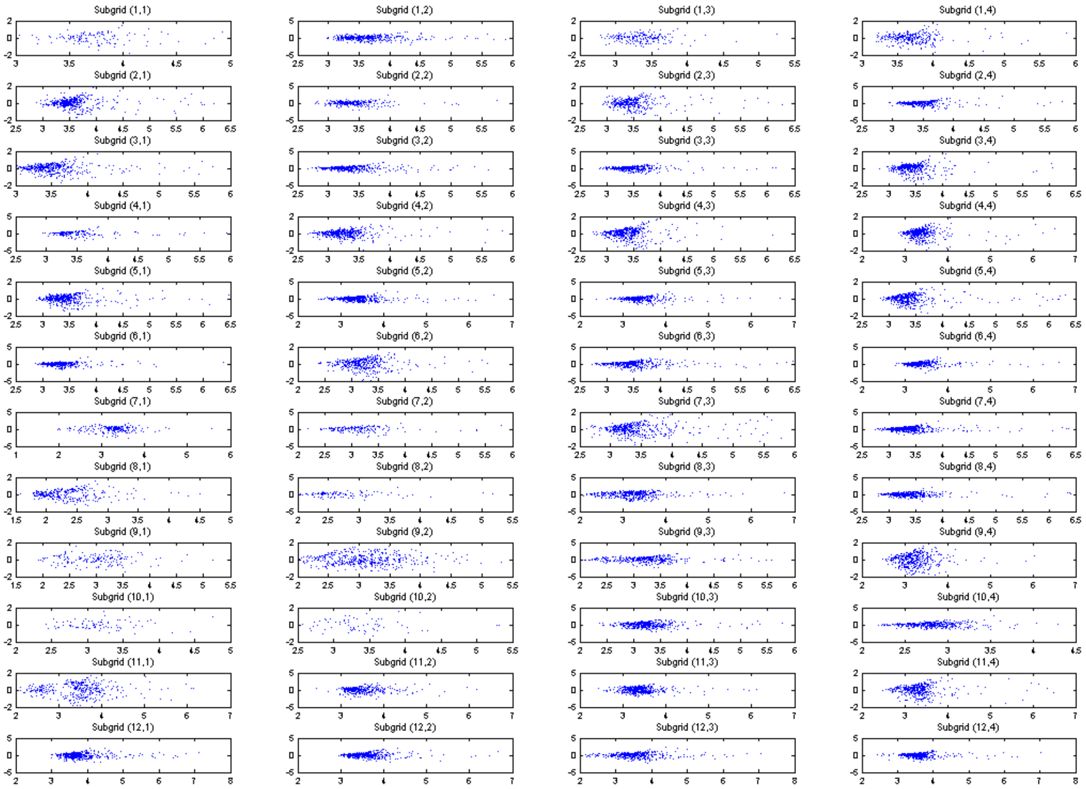

5.5. Expression Distributions

Slide expression distributions are histograms depicting the gene expression (log_2 ratio) distributions for specific arrays. They are useful for determining the nature of the data (e.g. the normality or the bimodality of gene expression distributions) and subsequently decide on which normalization method is suitable for specific datasets. They can also be used for quality control issues and for visualizing normalization effects. To create slide distribution histograms, the user should select an Analysis from the Analysis Object list and click **Plots → Slide Distributions**. The following window will appear:

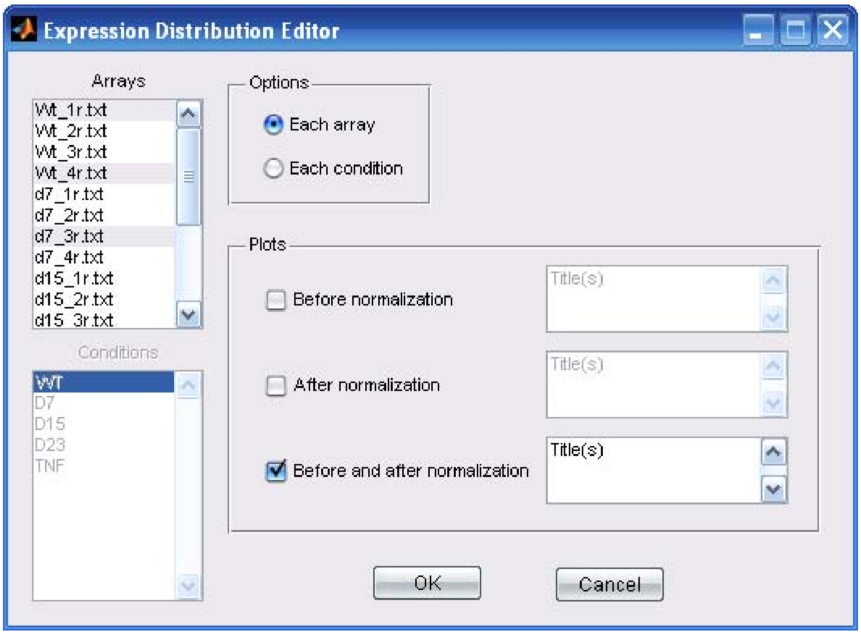

In the **Arrays** list the user can select one or multiple arrays for which to create gene expression distributions. The **Options** panel determines whether expression distributions will be created for individual arrays by selecting **Each array** (in this case, the **Arrays** list is activated and the user may select arrays from there) or for conditions by selecting **Each condition** (in this case, the **Arrays** list is deactivated and the user may select conditions from the **Conditions** list which is activated). As with MA plots, the user may choose to plot expression histograms for data before normalization, after normalization or make combined diagrams with data before and after normalization. Below there are several examples of expression histograms:

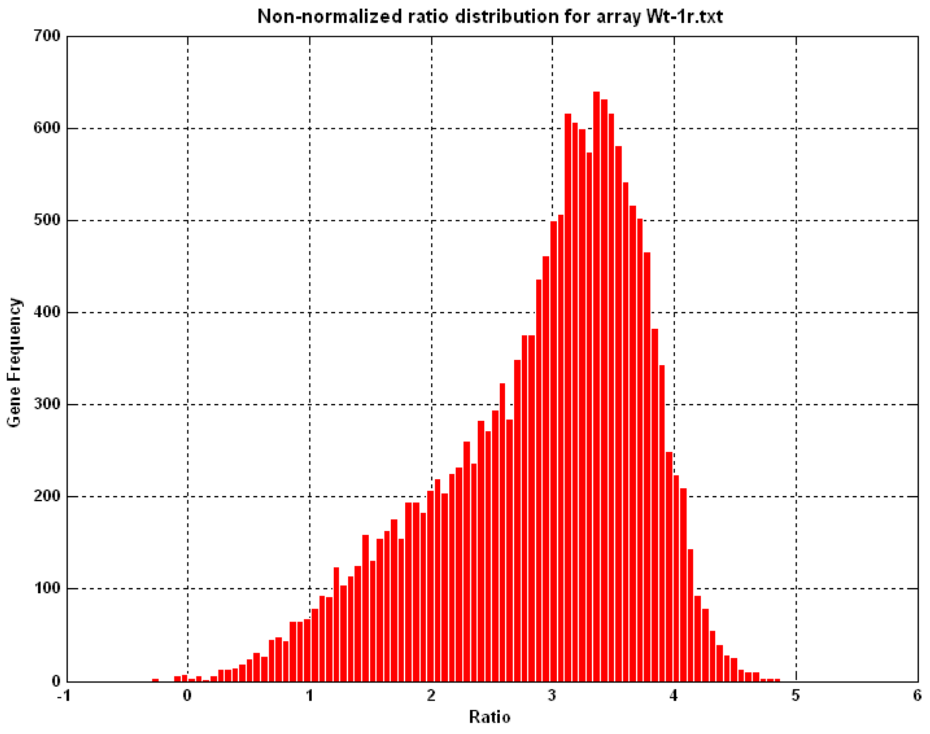

Non-normalized ratio distribution for array Wt-1r.txt
Gene Frequency
Ratio
0
100
200
300
400
500
600
700
-1
0
1
2
3
4
5
6

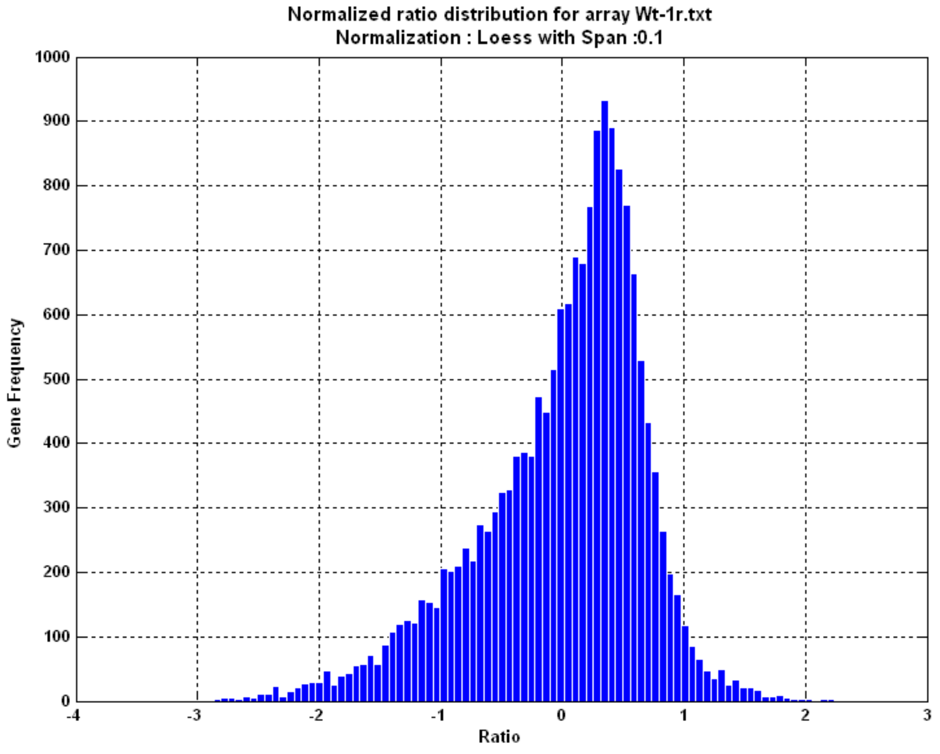

Normalized ratio distribution for array Wt-1r.txt
Normalization : Loess with Span :0.1
Gene Frequency
Ratio
0
100
200
300
400
500
600
700
800
900
1000
-4
-3
-2
-1
0
1
2
3

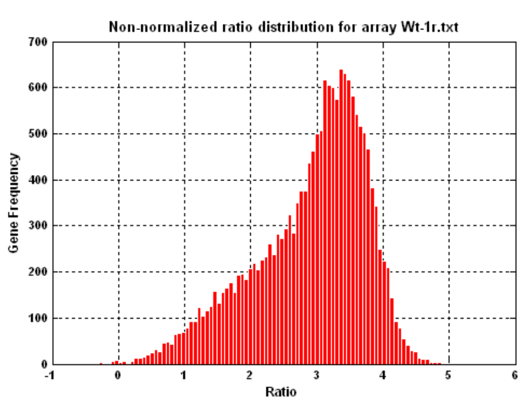
Non-normalized ratio distribution for array Wt-1r.txt
Gene Frequency
Ratio

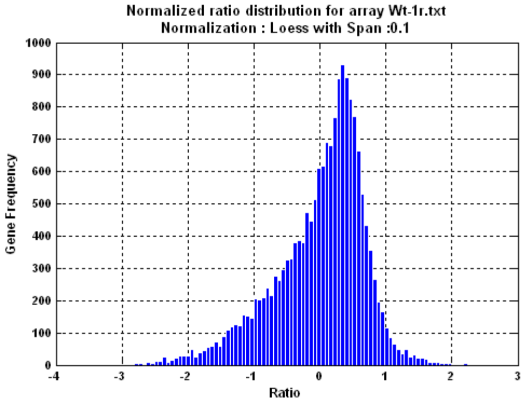
Normalized ratio distribution for array Wt-1r.txt
Normalization : Loess with Span :0.1
Gene Frequency
Ratio

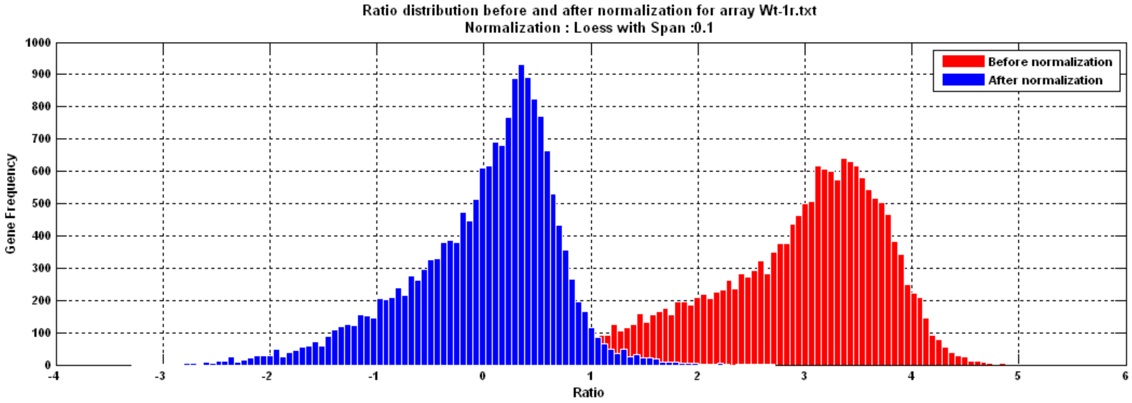
Ratio distribution before and after normalization for array Wt-1r.txt
Normalization : Loess with Span :0.1
Before normalization
After normalization
Gene Frequency
Ratio

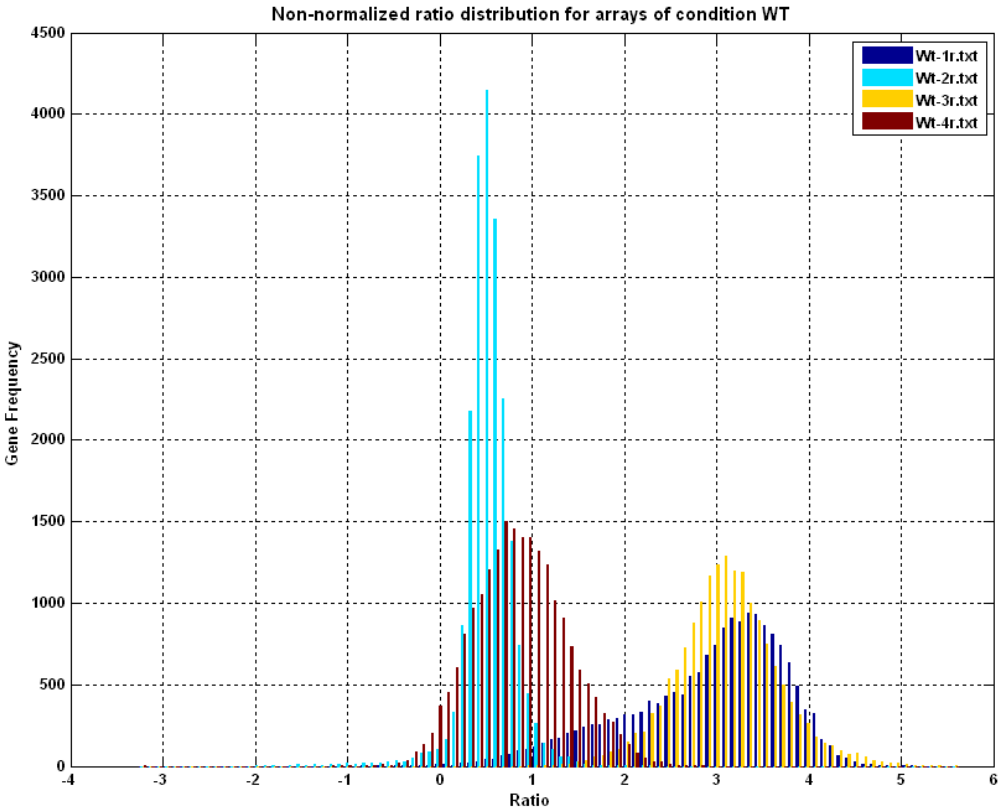
Non-normalized ratio distribution for arrays of condition WT
Wt-1r.txt
Wt-2r.txt
Wt-3r.txt
Wt-4r.txt
Gene Frequency
Ratio

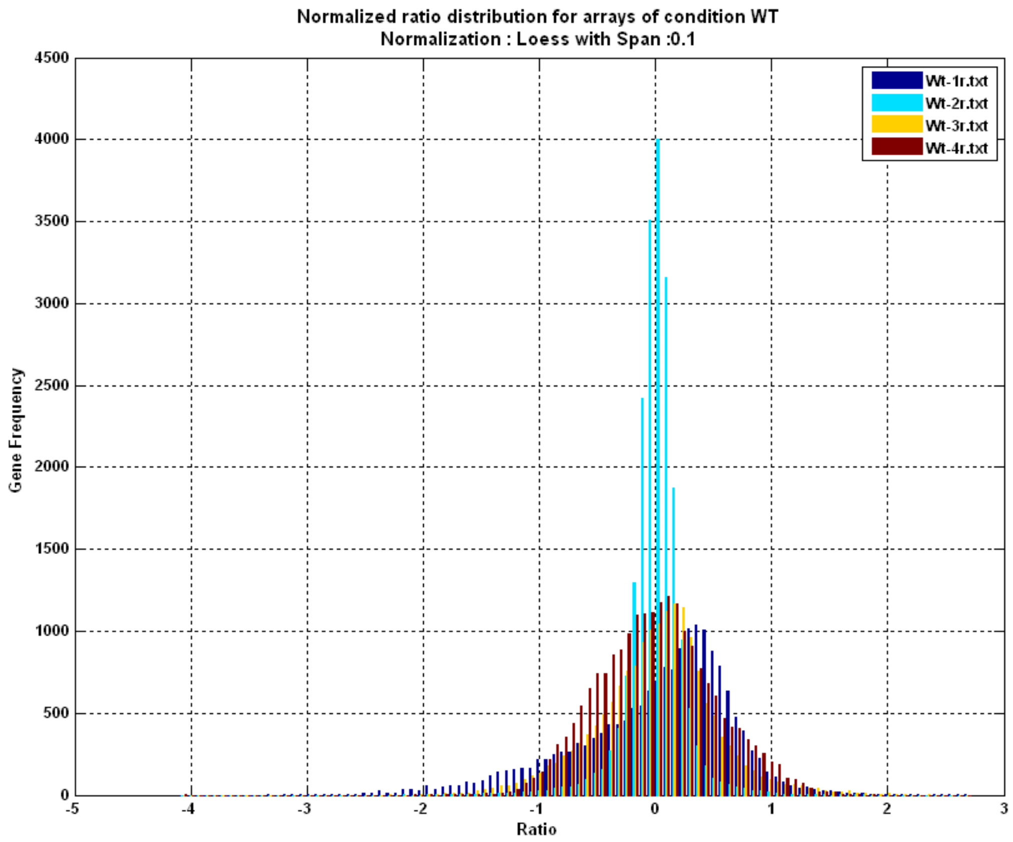
Normalized ratio distribution for arrays of condition WT
Normalization : Loess with Span :0.1
Gene Frequency
Ratio
Wt-1r.txt
Wt-2r.txt
Wt-3r.txt
Wt-4r.txt

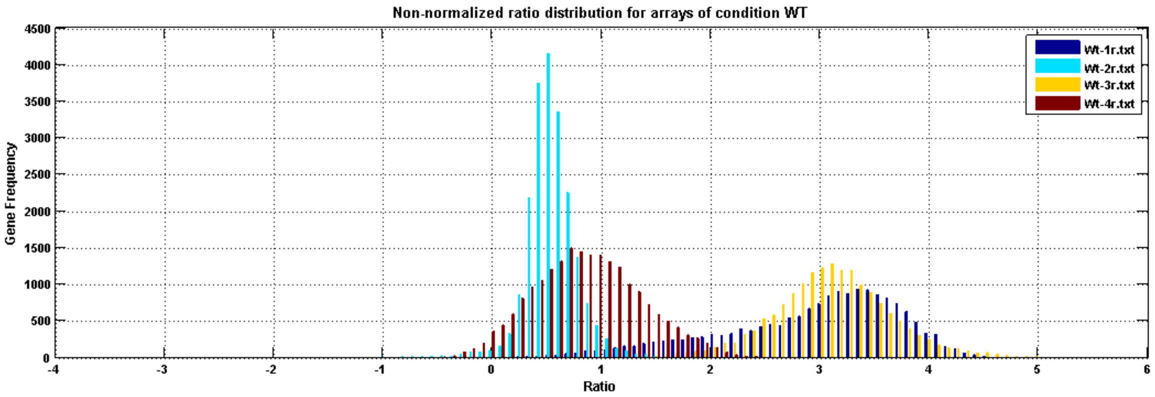
Non-normalized ratio distribution for arrays of condition WT
Gene Frequency
Ratio
Wt-1r.txt
Wt-2r.txt
Wt-3r.txt
Wt-4r.txt

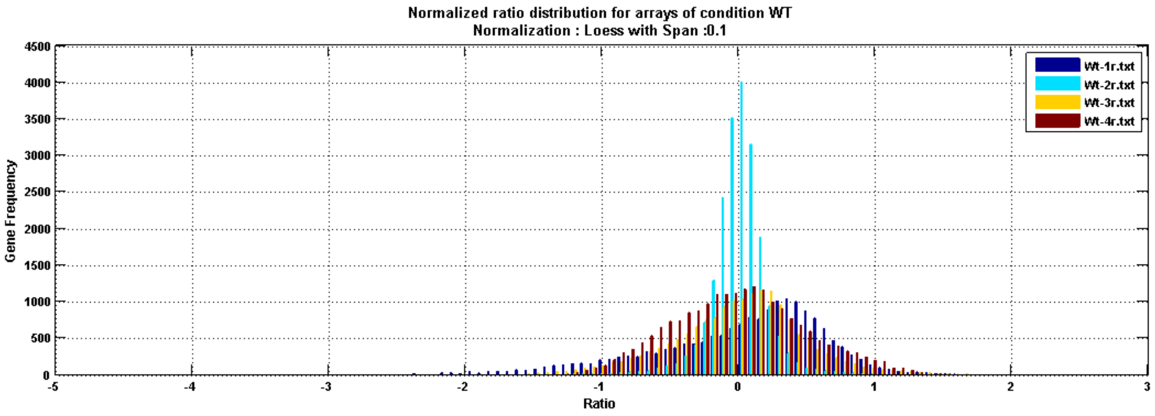
Normalized ratio distribution for arrays of condition WT
Normalization : Loess with Span :0.1
Gene Frequency
Ratio
Wt-1r.txt
Wt-2r.txt
Wt-3r.txt
Wt-4r.txt

The user should note that **Expression Distributions** in the **Plots** menu become available only after the normalization procedure.

5.6. Boxplots

In descriptive statistics, a boxplot is a convenient and commonly used way of graphically presenting groups of numerical data. A boxplot also indicates which observations, if any, might be considered outliers and is able to visually show different types of populations, without making any assumptions of the underlying statistical distribution. The spacings between the different parts of the box help indicate variance, skewness and identify outliers. For more information on boxplots the user should see (16).

In the case of microarray data, boxplots are used to summarize the gene expression distributions and identify their shape and several characteristics. They are useful for quality control as well as depicting differences between distributions among different slides and assessing the results of data normalization. Boxplots are available right after data importing. To create boxplots with ARMADA, the user should select an Analysis from the Analysis Object list and click **Plots** → **Boxplots**. The following window will appear:

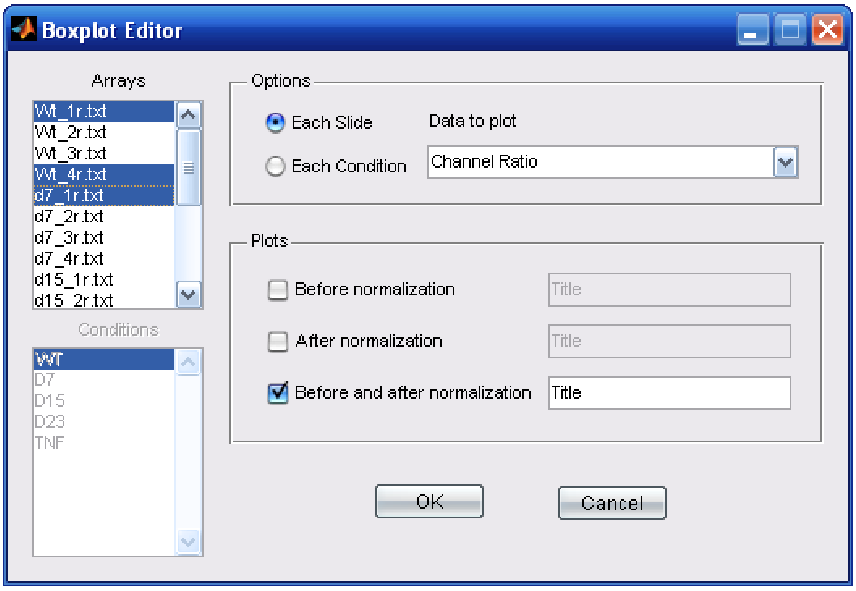

The interface is similar to the interface of **Expression Distributions** (5.3) with the selections in the **Options** and **Plots** panels denoting exactly the same configurations as with **Expression Distributions** and the only difference being the list **Data to plot**. This list contains the types of data that the user can visualize by using boxplots. All data apart from the Channel Ratio can be plotted only before normalization. For a description of the data types for which boxplots can be created, the user should look at the table in section 5.1 as they are exactly the same. The channel ratio is the $\log_2$

ratio between the two channels depicting gene expression. Below there are several examples of boxplots for data before and after normalization[10]:

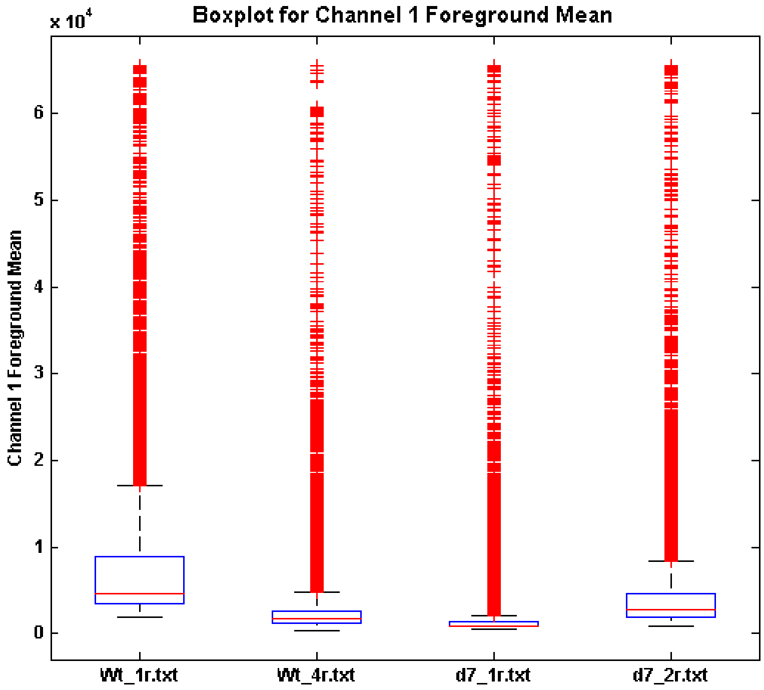

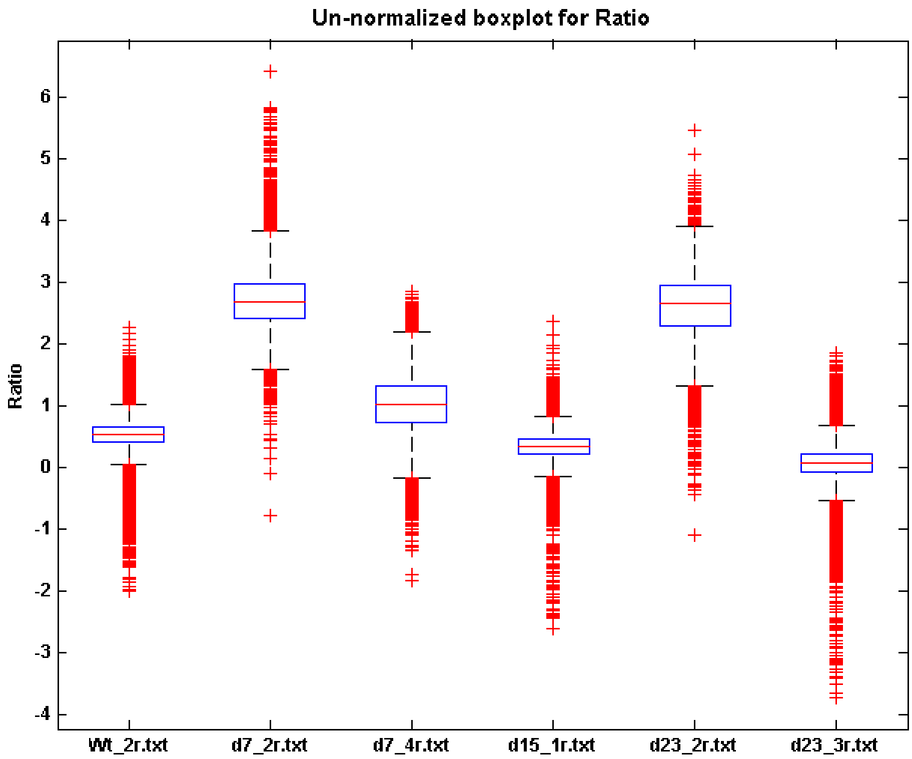

[10] By examining the boxplots before and after normalization, the effect of normalization is immediately seen: gene expression distribution are scaled and centered so that they can be compared using statistical tests.

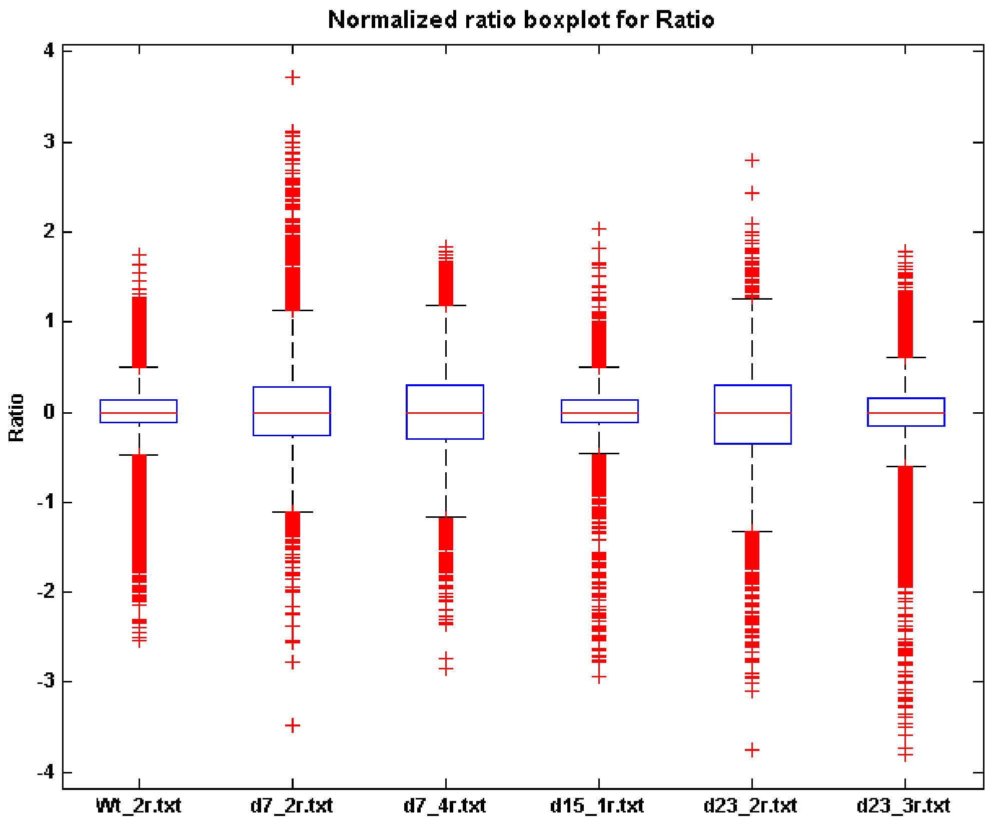
Normalized ratio boxplot for Ratio
Ratio
Wt_2r.txt
d7_2r.txt
d7_4r.txt
d15_1r.txt
d23_2r.txt
d23_3r.txt

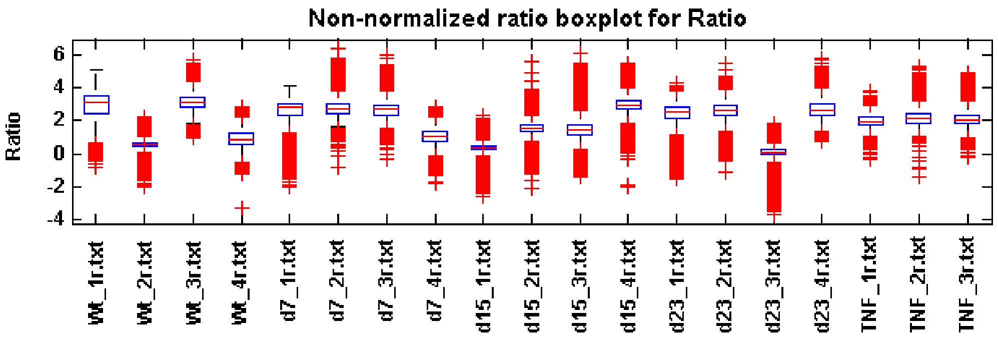
Non-normalized ratio boxplot for Ratio
Ratio

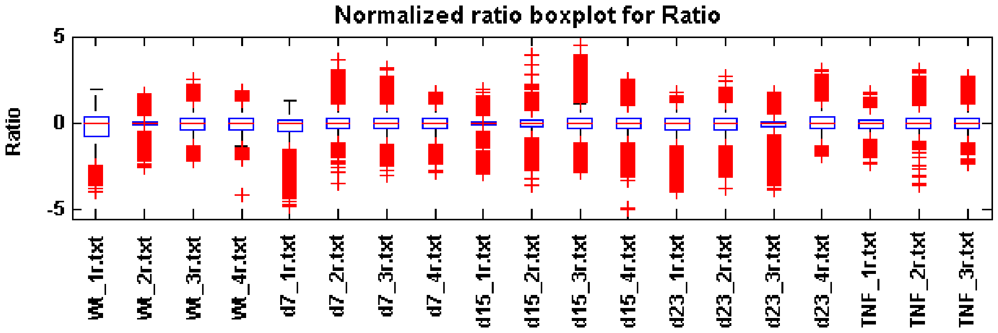
Normalized ratio boxplot for Ratio
Ratio

5.7. Volcano Plots

Volcano plots are useful for visualizing differentially expressed genes that have already been detected using a statistical test. In ARMADA, they are a plot of the $\log_2$ fold change on the horizontal axis and the quantity $-\log_{10}$(p-value) where the p-value comes from a statistical test (the user should see section 4). The volcano plot can be used to visualize differentially expressed genes, and also to show that large fold changes do not necessarily equal statistical significance or the opposite. Moreover, volcano plots can be created only for pairwise statistical comparisons (e.g. when performing a t-test between control and samples treated with a specific drug) and not in cases where the user seeks statistically significant genes among several conditions (e.g. using 1-way ANOVA to identify differentially expressed genes in at least one among five experimental configurations). Thus, the **Volcano Plots** command in the **Plots** menu is enabled only after a statistical test has been performed and when the selected Analysis in the Analysis Object list contains 2 experimental conditions. To create volcano plots, the user should select an Analysis from the Analysis Object list and click **Plots** → **Volcano Plots**. The following window will appear:

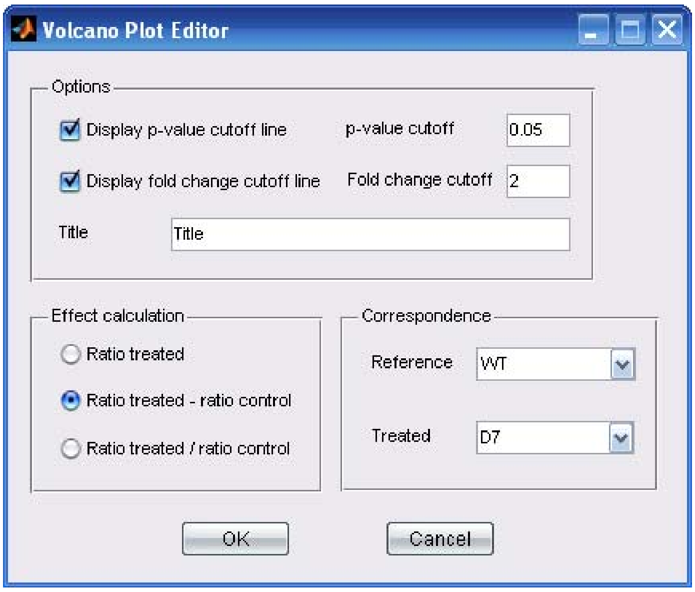

In the **Options** panel, the user should choose whether to display a p-value threshold line or not by checking or unchecking the **Display p-value cutoff line** box and provide a p-value threshold. The user can also select whether to display a fold change threshold line or not by checking or unchecking the **Display fold change cutoff line** box and provide a fold change threshold. As in MA plots (5.3) the fold change threshold is provided in natural scale but converted to $\log_2$ scale for the construction of fold change lines. A title for the volcano plot can be given in the **Title** field, else the field should be left empty or as is for an automatic title generation. In the **Correspondence** panel, the user should tell ARMADA which condition corresponds to the reference condition and which to the treated (e.g. with a drug) condition so that proper fold changes can be calculated. The table below explains the different options in the **Effect calculation** panel (how fold change is calculated in each case):

Option	Description
Ratio treated	This option should be chosen when the volcano plot should be created by defining the fold change as the log_2 ratio between channels in cases where there is only one experimental condition in the project or the current Analysis and Cy3 channel represents the reference samples while Cy5 channel represents the treated samples. In such case, it is also the only available option. It might also occur in other case studies depending on what the user wishes to see (e.g. when the analyst is interested to examine what is happening when comparing the treated sample to the common reference but not to the control which can be the 1st time point in a time point experiment). The fold change is thus $FC = log_2\left(\frac{Cy5_{treated}}{Cy3_{reference}}\right)$.
Ratio treated – ratio control	This option is the default in volcano plots in ARMADA when the project or the current Analysis includes more than one experimental condition. Is such cases, there is usually a control condition which has to be compared to several other conditions and the common reference labelled by Cy3 is common to all samples under examination. In these cases the fold change is calculated as $FC = log_2\left(\frac{Cy5_{treated}}{Cy3_{common\ reference}} \Big/ \frac{Cy5_{control}}{Cy3_{common\ reference}}\right) = log_2\left(\frac{Cy5_{treated}}{Cy3_{common\ reference}}\right) - log_2\left(\frac{Cy5_{control}}{Cy3_{common\ reference}}\right)$ as derived from logarithm properties.
Ratio treated/ratio control	This option should be used only when conducting statistical tests on non log_2 transformed data and should generally be avoided as it will not produce valid plots when used with log_2 transformed data[11].

After setting all the proper parameters, the user should click **OK**. Below there is an example of a volcano plot created with fold change calculated with the option **Ratio treated – ratio control** (which was proper for the example used):

[11] ARMADA log_2 transforms data by default. However, it is possible to get non log_2 transformed data when the user is importing from external data by selecting **Log ratio** options in the external data import wizard (2.5.3). For these reasons, this option is included in volcano plots.

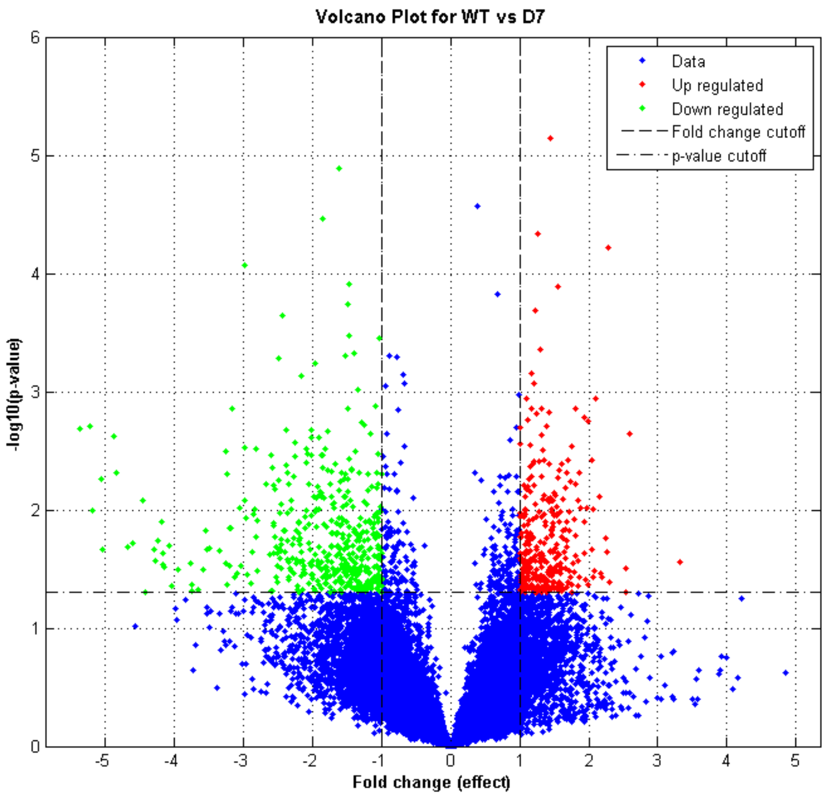

As with MA plots (5.3), volcano plot figures exist in two modes: data exploration mode, where the user can click on any data point on the figure and more specific information will be displayed for that point (the GeneID, ratio value, p-value etc.) and data selection mode where the user can select several data points to export. Below, there is an example of a volcano plot in data selection mode:

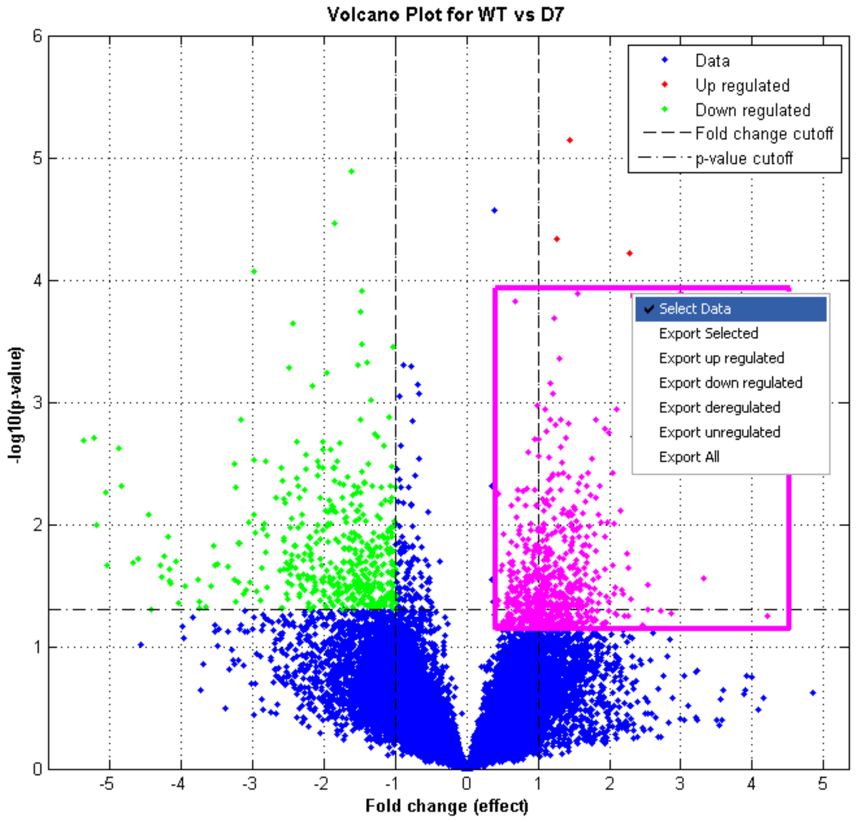

If the user right-clicks inside the volcano plot area, the following menu will appear:

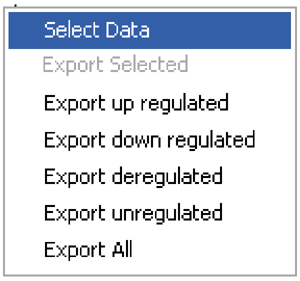

The following table explains the functions of the items displayed in the menu appearing after right-clicking:

Name	Function
Select Data	Switches between data exploration and data selection modes.
Export Selected	While on selection mode, exports data points defined by the rectangular selection area. The user must right-click on one of the *edges* of the selection area.
Export up regulated	Exports up regulated genes (red data points). Available only if fold change and/or p-value thresholds have been provided.
Export down regulated	Exports down regulated genes (green data points). Available only if fold change and/or p-value thresholds have been provided.
Export deregulated	Exports up and down regulated genes (red and green data points). Available only if fold change and/or p-value thresholds have been provided.
Export unregulated	Exports up unregulated genes (blue points). If fold change and p-value thresholds have not been provided, exports all data points.
Export All	Exports all data points, regardless of mode status.

5.8. Expression Profiles

Expression profile plots allow the analyst to follow the expression of specific genes across different experimental conditions or across different time points in a time course experiment with the help of a graphic which displays the gene expression (their log_2 channel ratio) against the different experimental conditions or time points. Expression profile plots are especially useful for the display of expression patterns after the application of a clustering algorithm (4.3) such as k-means clustering (4.3.2) as they allow the user to identify specific patterns and give initiatives for further research. Expression profile plots are also helpful for overlying the expression of many genes across different conditions and identify several phenomena such as genes with reverse expression, early or late deregulated genes (compared to each other others) etc. To create expression profile plots, the user should select an Analysis from the Analysis Object list and click **Plots → Expression Profiles**. The following window will appear:

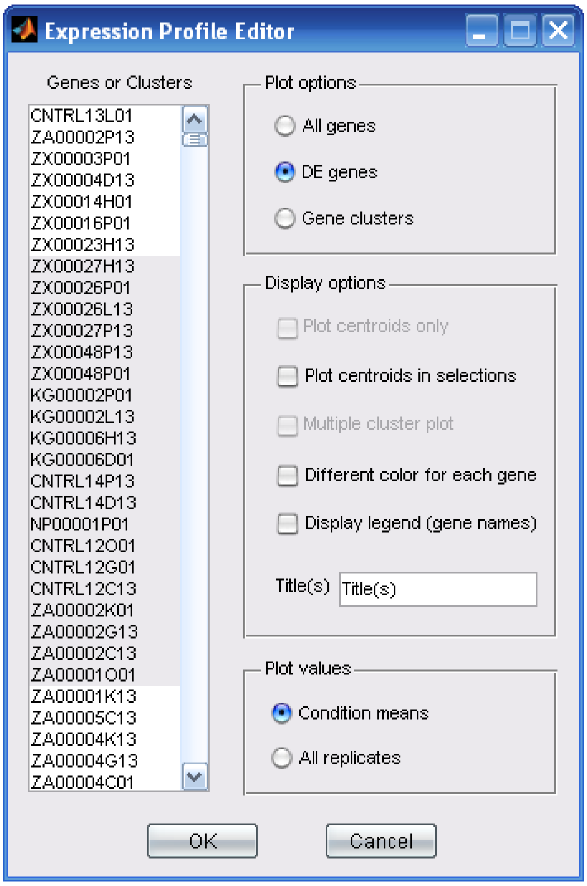

In the left part of the expression profile preferences window, the **Genes or Clusters** list displays the GeneIDs or the gene cluster numbers for the selected Analysis from the Analysis Object list, depending on the selection on the **Plot options** panel. The following table explains in detail all the user options from the **Plot options**, **Display options** and **Plot values** panels:

	**Option**	**Description**
**Plot options**	All genes	This option gives the ability to plot expression profiles using normalized values for several genes out of all the genes that passed the preprocessing filtering steps. The user may select genes by their GeneID from the list **Genes or Clusters** on the left of the expression profile preferences window.
	DE genes	This option gives the ability to plot expression profiles using normalized values for several genes but only from the **D**iferentially **E**xpressed genes that were determined after the statistical selection process (4.1). The user may select genes by their GeneID from the list **Genes or Clusters** on the left of the expression profile preferences window. If statistical selection has not been performed, this option will not be available.

**Display options**	Gene clusters	This option gives the ability to plot expression profiles using normalized values for genes belonging to clusters determined by the clustering algorithm used after the statistical selection process (4.3). The user may select genes by their cluster number from the list **Genes or Clusters** on the left of the expression profile preferences window. If clustering has not been performed, this option will not be available.
	Plot centroids only	This box is enabled only for the **Gene clusters** option in the **Plot options** panel. If checked, it will produce expression profile plots but instead of using all the genes for each cluster, it only uses the gene centroid[12] expression pattern reflecting the general deregulation motif of the genes belonging to each cluster. The **Plot centroids only** box is enabled only if k-means (4.3.2) or fuzzy c-means (4.3.3) clustering has been performed and disables the **Plot values** panel as the centroids have been calculated by the clustering algorithm.
	Plot centroids in selections	This box is available for all the options in the **Plot options** panel. If checked, the expression profile plots will also display a centroid calculated using the mean expression of the selected genes or the genes belonging to the each gene cluster. Error bars are also created displaying expression standard deviation.
	Multiple cluster plot	This box is enabled only for the **Gene clusters** option in the **Plot options** panel. If checked, expression profiles will be displayed in one figure with multiple plots instead of multiple figures (one for each cluster).
	Different color for each gene	This box, if checked will display the expression of each gene with a different color instead of using only one color for each gene. It should be checked when plotting gene clusters because it offers better visualization.
	Display legend (gene names)	This box, if checked will create a legend in the figure, containing GeneIDs that correspond to different lines in the expression profile plot. It should not be used when plotting a large number of genes for proper visualization purposes.
	Title(s)	By filling this field, the user can provide title(s) for plots. As with other plot preference windows in ARMADA, the number of titles should match the number of figures to be created (e.g. 5 titles for 5 figures of different clusters).
**Plot values**	Condition means	This option, if chosen will produce expression profile plots where gene expression is calculated from the mean of all the arrays for each condition.
	All replicates	This option, if chosen will produce expression profile plots using expression values from all replicates for each condition.

Below, there is an example of an expression profile plot for 11 genes selected from the list of differentially expressed genes after applying a statistical test. The plot was produced with the option **DE genes** in the **Plot options** panel chosen, the **Different color for each gene** and **Display legend (gene names)** boxes checked and the **All replicates** option from the **Plot values** panel chosen:

[12] Cluster centroids are usually calculated by averaging the expression of all the genes belonging to a cluster, defining thus a 'meta-gene' which reflects the expression pattern of the entire cluster.

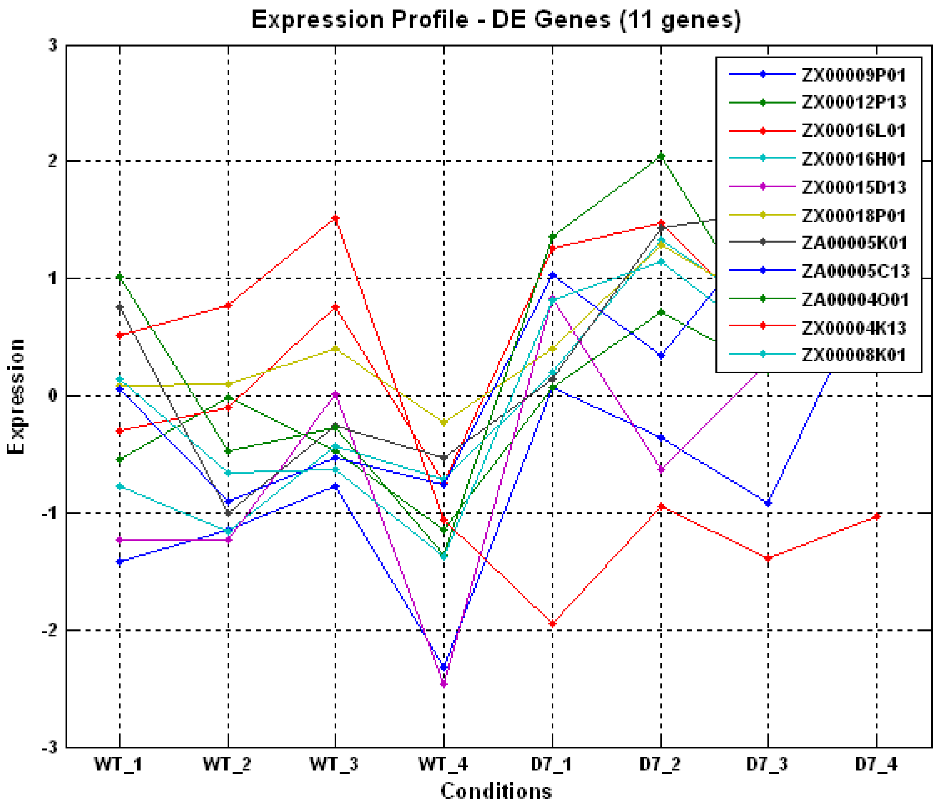

To illustrate another example, the following figure presents an expression profile plot for 10 gene clusters presented in a graph with multiple plots. The plot was produced with the option **Gene clusters** in the **Plot options** panel chosen, the Plot centroids in selections, Multiple cluster plot boxes checked and the **Condition means** option from the **Plot values** panel selected:

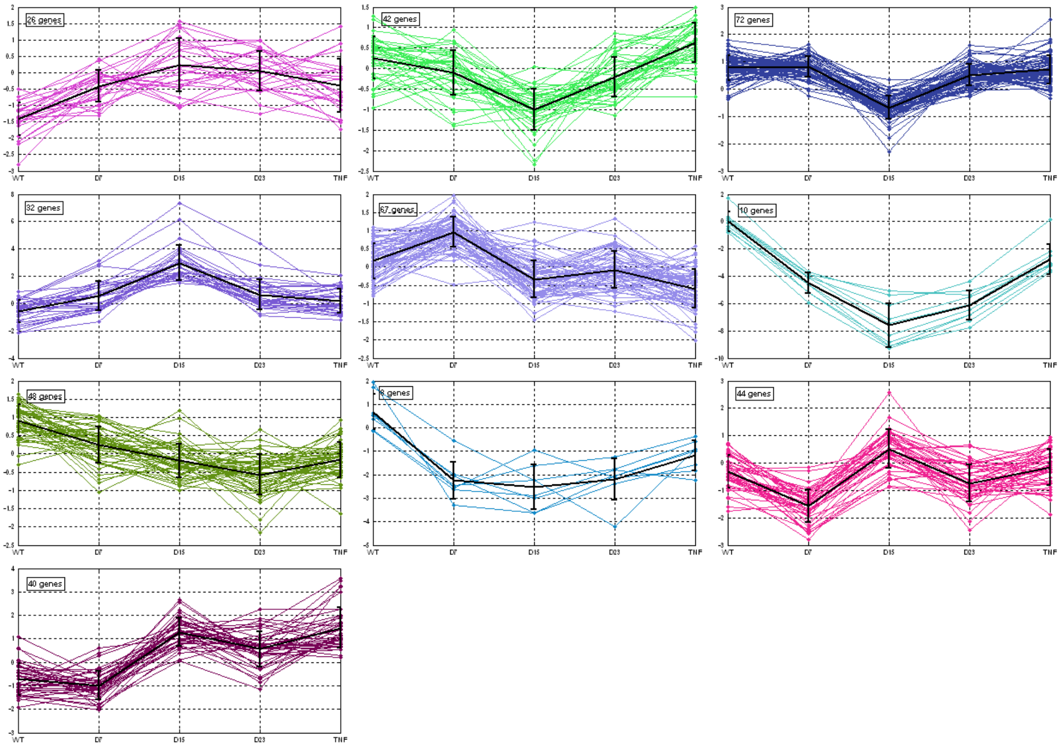

The following figure was created with exactly the same options but with the **Plot centroids only** box checked and the **Plot centroids in selections** unchecked.

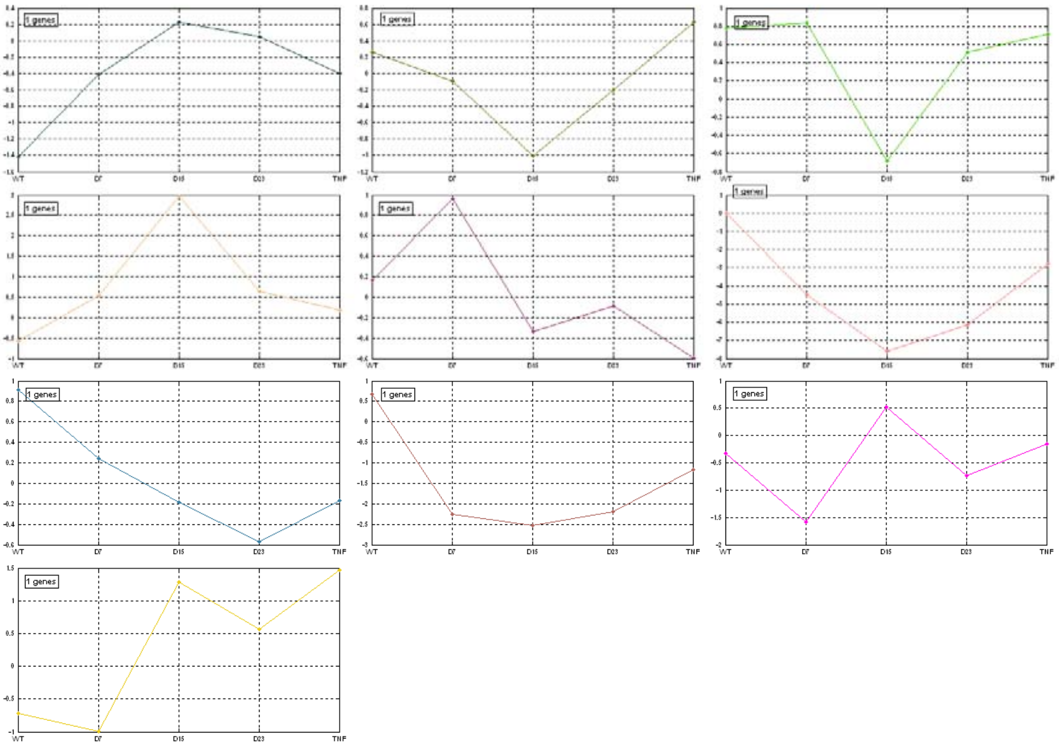

As with most figures in ARMADA, if the user click on specific data points, more information on that data point will be displayed. It should be noted that **Expression Profiles** become available in the **Plots** menu after the normalization process.

6. Exporting Data

This section presents the various data types that can be exported using ARMADA's exporting functionalities as well as how to export and save figures. In summary, the subsection of this section explains how the user can customize and export normalized gene lists, differentially expressed gene lists and cluster lists, as well as how to export figures in various formats using MATLAB's figure interface and controls.

6.1. Exporting gene lists

After completing several steps of data analysis and exploration, the user can export two kinds of gene lists from ARMADA: normalized gene lists and differentially expressed gene lists. Both lists can contain the same data (apart from several statistics which are produced only after the application of statistical tests). Exporting normalized gene lists becomes available right after the normalization step while exporting differentially expressed gene lists only after the statistical selection process. To export normalized gene lists, the user should select an Analysis from the Analysis Object list on the main window and click on **File** → **Export Data** → **Normalized Genes List**, or right-click on the selected Analysis from the Analysis Object list and select **Export Normalized List**. To export differentially expressed gene lists, the user should select an Analysis from the Analysis Object list and click **File** → **Export Data** → **DE Genes List**, or right-click on the selected Analysis from the Analysis Object list and select **Export DE List** or click on the **Export DE List** shortcut button on the main window. In all cases the user will be prompted to specify a location for the output file to be saved at.

The normalized and differentially expressed gene lists are text tab delimited or Excel files which contain data separated in different columns. The user is able to specify the output file format (Excel or text tab delimited) as well as the data fields to be exported by clicking on **File** → **Export Settings** → **Gene Lists**. The following preferences window will appear:

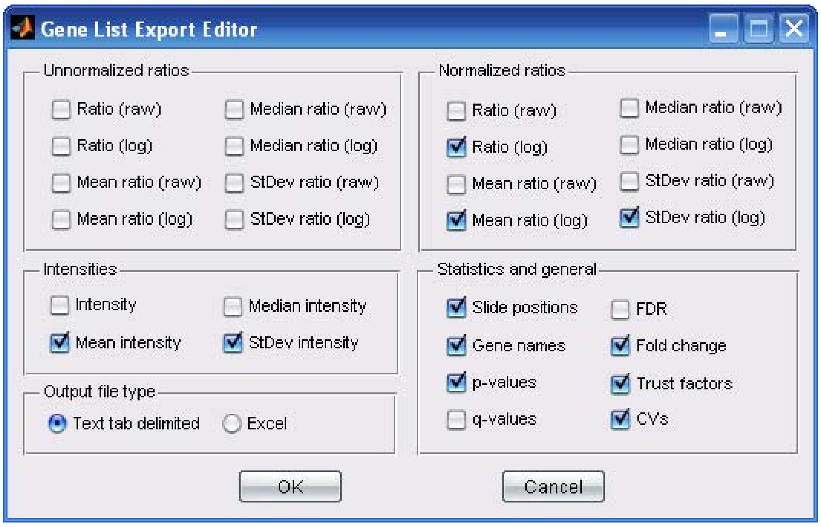

The following table explains the data types that are exporting by checking each of the boxes in the gene list export preferences window (the term ‘ratio’ denotes the ratio between channels and ‘intensity’ the intensity values calculated from the two channel signals):

	**Option**	**Description**
**Unnormalized ratios**	Ratio (raw)	The un-normalized ratio in natural scale for each replicate of each experimental condition.
	Ratio (log)	The un-normalized ratio in $\log_2$ scale for each replicate of each experimental condition.
	Mean ratio (raw)	The mean un-normalized ratio of the replicates for each condition in natural scale.
	Mean ratio (log)	The mean un-normalized ratio of the replicates for each condition in $\log_2$ scale.
	Median ratio (raw)	The median un-normalized ratio of the replicates for each condition in natural scale.
	Median ratio (log)	The median un-normalized ratio of the replicates for each condition in $\log_2$ scale.
	StDev ratio (raw)	The standard deviation of the replicates un-normalized ratio for each condition in natural scale.
	StDev ratio (log)	The standard deviation of the replicates un-normalized ratio for each condition in $\log_2$ scale.
**Normalized ratios**	Ratio (raw)	The normalized ratio in natural scale for each replicate of each experimental condition.
	Ratio (log)	The normalized ratio in $\log_2$ scale for each replicate of each experimental condition.
	Mean ratio (raw)	The mean normalized ratio of the replicates for each condition in natural scale.
	Mean ratio (log)	The mean normalized ratio of the replicates for each condition in $\log_2$ scale.
	Median ratio (raw)	The median normalized ratio of the replicates for each condition in natural scale.
	Median ratio (log)	The median normalized ratio of the replicates for each condition in $\log_2$ scale.
	StDev ratio (raw)	The standard deviation of the replicates normalized ratio for each condition in natural scale.
	StDev ratio (log)	The standard deviation of the replicates normalized ratio for each condition in $\log_2$ scale.
**Intensities**	Intensity	The intensity for each replicate of each experimental condition.
	Mean intensity	The mean intensity of the replicates for each condition.
	Median intensity	The median intensity of the replicates for each condition.
	StDev intensity	The standard deviation of the replicates intensity for each condition.
**Statistics and general**	Slide positions	The numbers denoting each gene’s unique positioning on the microarray slide (the user should see 2.5 and 2.5.2)
	Gene names	The genes’ identifier names (usually the chip manufacturer’s identification names) which serve as a textual identification for each gene.
	p-values	The p-value (or adjusted p-value) scores for each gene returned by the statistical test applied for the identification of differentially expressed genes (the user should see 4.1).
	q-values	The q-values returned by the False Discovery Rate estimation procedure (the user should see 4.1).
	FDR	The False Discovery Rate estimates returned (the user should see 4.1).

Output file type	Fold change	The fold change estimates as calculated by the process explained in section 4.2 for each condition.
	Trust factors	The trust factor estimates calculated by the process explained in section 4.1 for each condition.
	CVs	The coefficients of variation (StDev/Mean) for each condition.
	Text tab delimited	The output files are of text tab delimited format and can be opened by any text editor or spreadsheet editing programs (such as MS Excel). Text tab delimited files can also easily be imported to other tools for process or easily stored in local databases.
	Excel	The output files are of Excel format. They can be opened and processed with MS Excel or Open Office tools but they are larger than and not as flexible as text tab delimited files.

After selecting the preferred fields to be exported, the user should click **OK** and all files that are exported from that point forward will contain the data fields specified. If the user wishes to change the number of fields exported, the export gene list preferences window can be used again. It should be noted that the tables presented in ARMADA's main window and described in sections 2.6.9 and 2.6.10 will contain the fields specified in the export gene list preferences window.

6.2. Exporting gene cluster lists

The gene cluster files contain the results of the clustering processes (the user should see 4.3) and their format is standard. To export gene cluster files, the user should click on **File → Export Data → Gene Clusters List**, or right-click on the selected Analysis from the Analysis Object list and select **Export Clusters List** or click on the **Export Clusters** shortcut button on the main window. The user will then be prompted to select the storage location of the clusters file. As with the gene list files, the cluster output files can be either text tab delimited or Excel files. The following table explains the meaning of each header in the cluster files:

**Column name**	**Description**
Slide Position	The numbers denoting each gene's unique positioning on the microarray slide (the user should see 2.5 and 2.5.2)
GeneID	The genes' identifier names (usually the chip manufacturer's identification names) which serve as a textual identification for each gene.
ClusterNo	The cluster ID the genes belong to.
Feature depending on clustering algorithm	The value of a specific feature which can be different for each algorithm, e.g. for hierarchical clustering, the Silhouette value is returned, while for k-means clustering, the sum of distances from cluster centroid for each gene is returned. The user should see 4.3 for further details.
p-value	The p-value (or adjusted p-value) returned from statistical test applied.
Data columns	The rest of the columns until the end of the file contain normalized expression values according to what values the clustering is based on (e.g. means or replicates, the user should see 4.3).

Additionally, in the case of fuzzy c-means clustering, each gene's membership coefficient (the user should see 4.3.3) is returned to the c columns following the data columns.

6.3. Exporting figures

While the user could utilize simple screen capture tools (or even simply pressing the PrtScn key) to capture the diagrams created by ARMADA, it is better to use MATLAB's figure saving and exporting controls even if MATLAB is not present on the machine[13]. The following figure is used as an example of image or diagram exporting:

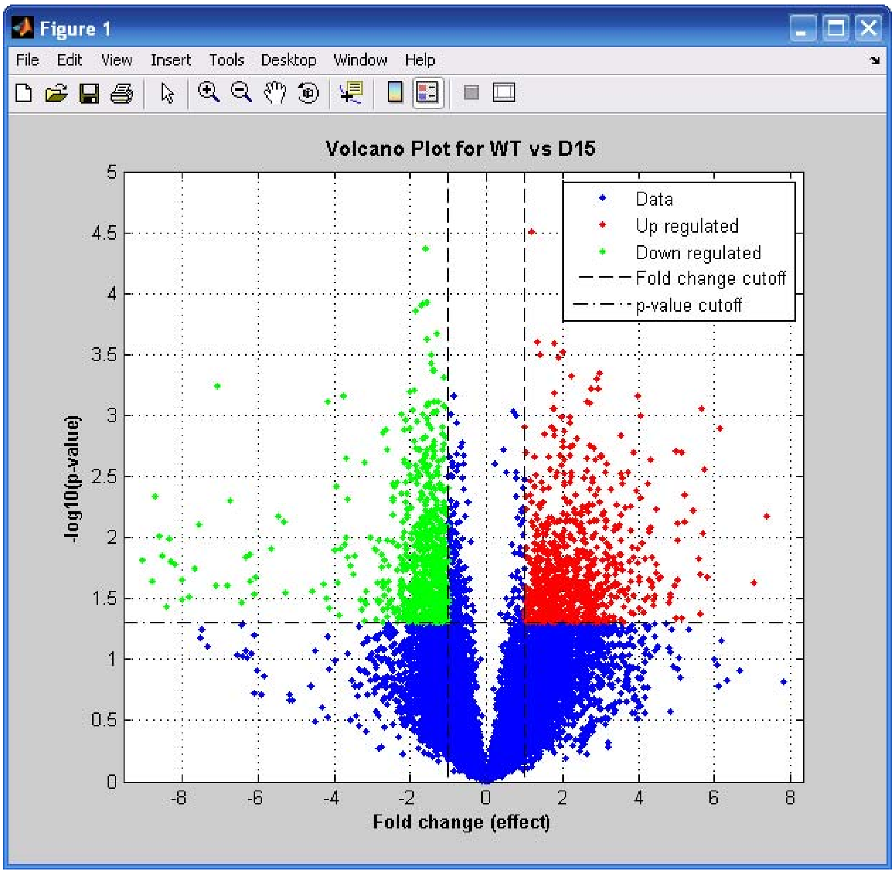

By clicking on **File** → **Save As…** the user is able to save the figure in any of the widely used image formats (e.g. .jpg or .png formats). To obtain more results of better quality the user should use the figure export setup which is accessible by clicking **File** → **Export Setup…**where a lot more parameters can be set in order to optimize the graphical output. For more information, the user should consult http://www.mathworks.com/access/helpdesk/help/techdoc/matlab.html under the Graphics → Preparing Graphs for Presentation section.

6.4. Exporting to .mat files

As ARMADA is addressed to both experienced and inexperienced users, the more experienced user can export the results from several analyses steps to a .mat file and import it to MATLAB for further processing with MATLAB's internal algorithms or use specific functions from several toolboxes. To be able to read ARMADA .mat file exports, MATLAB 7.1 (R13SP3) should be

[13] The purpose of ARMADA is to allow users not experienced with MATLAB to use the program and to offer a free analysis tool which needs only the MATLAB Component Runtime to run and not necessarily MATLAB installed on the user's machine.

installed on the user's machine and the Statistics Toolbox should be present. To export ARMADA results to .mat files, the user should click on **File** → **Export Settings** → **MATLAB Workspace**. The following window will appear:

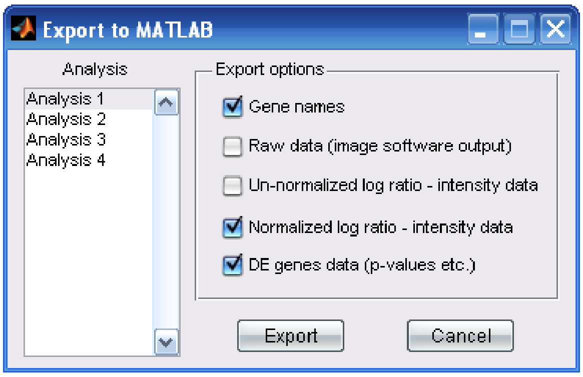

From there, the user can select several data types to be exported to the .mat file which then can be opened from within MATLAB for further process. The following table describes the data exporting choices of the export to MATLAB preferences window:

**Option**	**Description**
Gene names	Exports the chip manufacturer's gene identification which are determined from the input files (the user should see 2.5 for further details).
Raw data (image software output)	Exports the raw data provided with the input files in structure format. Each input file is a structure with the following fields (some might be missing, depending on the type of the input files) which are described here very briefly, as the user can find information on these fields in section 2.5:

**Field name**	**Short description**
Header	The header of the input file.
Blocks	Array subgrid blocks.
ArrayRow	Row meta-coordinates.
ArrayColumn	Column meta-coordinates.
Row	Row coordinates.
Column	Column coordinates.
ColumnNames	File column names.
Number	Gene numbering (slide positions).
GeneNames	Gene identifiers.
ch1Intensity	Channel 1 signal mean.
ch2Intensity	Channel 2 signal mean.
ch1IntensityMedian	Channel 1 signal median.
ch2IntensityMedian	Channel 2 signal median.
ch1IntensityStd	Channel 1 signal standard deviation.
ch2IntensityStd	Channel 2 signal standard deviation.
ch1Background	Channel 1 background mean.
ch2Background	Channel 2 background mean.

	ch1BackgroundMedian	Channel 1 background median.
	ch2BackgroundMedian	Channel 2 background median.
	ch1BackgroundStd	Channel 1 background standard deviation.
	ch2BackgroundStd	Channel 2 background standard deviation.
	IgnoreFilter	Spot flags
	Indices	MATLAB indices created if meta-coordinates are provided and used to create array images.
	Shape	MATLAB matrix defining subgrid block orientation.
Un-normalized log ratio – intensity data	Un-normalized log2 ratio and intensity data for all arrays in the Analysis Object.	
Normalized log ratio – intensity data	Normalized log2 ratio and intensity data for all arrays in the Analysis Object.	
DE genes data (p-values etc.)	Statistics and data for differentially expressed genes.	

It should be noted that the option boxes in the export to MATLAB preferences window are available only if the corresponding procedures have been performed (e.g. DE genes data will not be available if statistical operations have not been performed). Also, the user may select different data to be exported for each Analysis Object.

After making the necessary selections, the user should click **Export** and will be prompted to select a storage location for the .mat file. The .mat file which is created with the above procedure consists of a structure of length equal to the Analysis Objects in the project from within the .mat file was created. Each structure in the structure matrix has the following fields (displayed below in tree format):

```
Analysis
|------GeneNames
|------RawData
|------UnNormalized
|               |---Ratio
|               |---Intensity
|
|------Normalized
|               |---Ratio
|               |---Intensity
|
|------DEGenesStats
```

The user can easily correlate the field names with the options described in the table above. Each leaf of the above tree (apart from the GeneNames field which is a cell array of strings and the RawData field which is a cell array of structures with fields describe in the table above) is a MATLAB object of class 'dataset'. For more information on datasets and how they can be handled the user should consult http://www.mathworks.com/access/helpdesk/help/toolbox/stats/ under the Organizing Data → Statistical Arrays → Dataset Arrays section. MATLAB includes internal functions to handle dataset objects and convert them to simple matrices which can be then used with any MATLAB toolbox.

7. Other Tools

This section presents some additional analysis tools implemented in ANDFROMEDA. These tools are the principal component analysis tool which allows pattern discovery between genes belonging to different experimental conditions, the Gap statistic which allows the determination of the number of clusters in a dataset using one of the supported clustering algorithms, the Batch Programmer module which allows to perform multiple analysis steps in an automated way and the Annotator module which allows the easy annotation of the gene and cluster lists created by ARMADA.

7.1. The Principal Component Analysis tool

Principal Component Analysis (PCA) is a statistical pattern analysis technique for determining the key variables in a multidimensional data set that explain the differences in the observations and is very useful for analysis simplification and visualization of multidimensional data sets. Given m observations (samples or arrays) on n variables (genes) which form an $m \times n$ data matrix, the goal of PCA is the reduction of the data matrix dimensionality by finding r new variables, where r is less than n. These r new variables are termed principal components and together they account for as much of the variance in the original n variables as possible while remaining mutually uncorrelated. For more information on PCA for gene expression datasets derived from microarray experiments, the user should consult (17). The user can conduct PCA in ARMADA by selecting an Analysis from the Analysis Object list and click on **Tools** → **Principal Component Analysis**. The following window will appear:

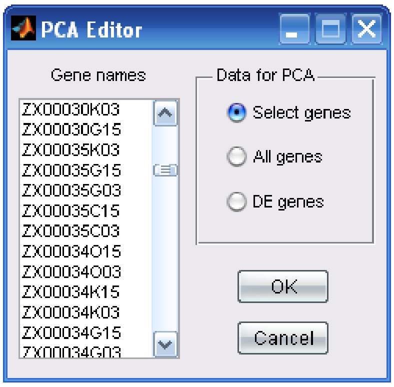

From there, the user is able to choose to perform PCA on the gene selected from the list on the left, to perform PCA on all genes using their normalized gene expression values after the trust factor filtering step (the user should see 4.1) or to perform PCA on the differentially expressed genes selected after the application of a statistical test. After making the necessary selections, the user should click **OK** and the following figure will appear[14]:

---

[14] The PCA module uses a slightly altered version of the `mapcaplot` function of the Bioinformatics Toolbox of MATLAB.

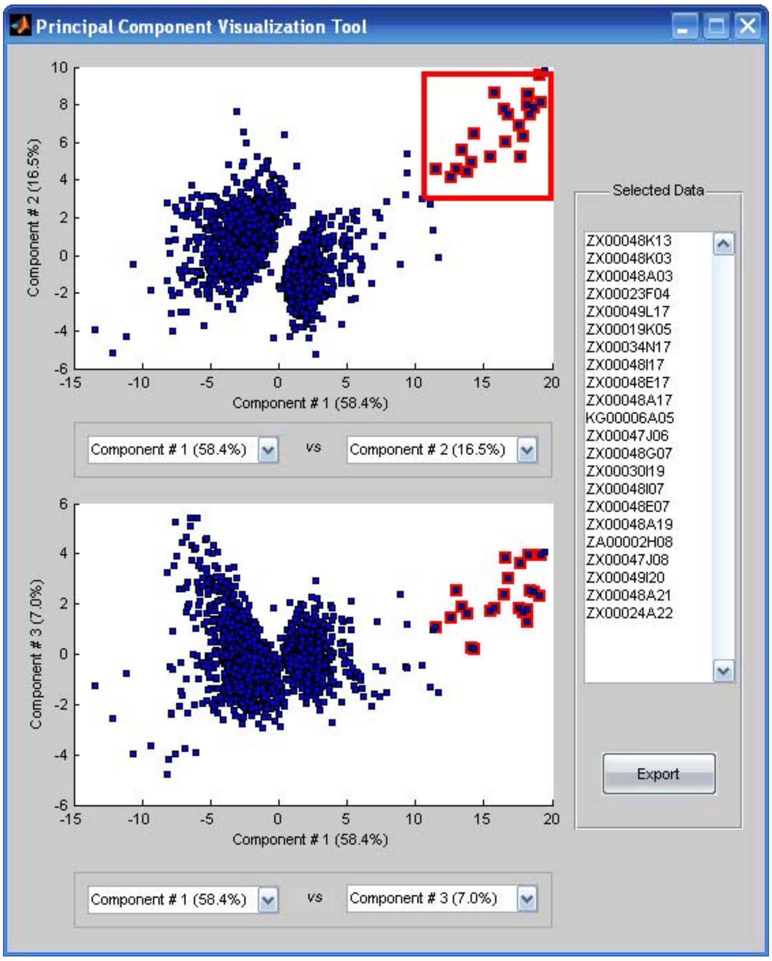

This figure contains two main panels: the upper panel presents the projections of the data matrix on the 2-dimensional plane defined by the first two principal components, which account for the largest and the 2nd larger percentage of the variance observed in the data matrix (the data matrix has been defined using the PCA preferences window above) respectively. Each point inside the diagram in the upper panel corresponds to a gene. The percentage of the data variance which each principal component ‘caught’ can be seen inside the parenthesis on each axis label. The bottom panel presents the projection of the data matrix on the 2-dimesional plane defined by the 1st and 3rd principal components respectively. The user can use the popup lists in order to change the data projection plane by changing the principal components displayed. The user can also click on each data point (gene) to view its label and also select data (as in MA or volcano plots). The names of the selected genes are displayed in the list on the right part of the figure and user can export the selected genes coupled with their expression values in text tab delimited format by clicking the **Export** button. In addition, the user can move the rectangular area (‘window’) over other data points and the list on the right will be updated with the genes that are inside the moving window each time.

The main goal of PCA is to transform the original data in such a way so as to reveal any possible patterns that can help the researcher to distinguish among different experimental conditions. In the example used to create the above figure, it is obvious that the first 2 principal components (presented in the upper panel) are able to account for a large percentage of variance in the dataset

and this can also be seen by the shape of the projected data where there are two distinguishable clouds of data points. Genes furthest from the whole swarm center can be thought of as genes that their expression is different and can separate among different experimental configurations. The PCA tool becomes available after the statistical selection procedure.

7.2. The Gap Statistic tool

A major problem when trying to discover groups in data without the help of a response variable (e.g. when trying to discover groups of similarly deregulated genes without having a prior idea on how many are these groups) is how to estimate the optimal number of these groups or 'clusters'. One way to partially solve this problem is the Gap statistic introduced by Tibshirani *et al.* in 2001 and has been applied in microarray data. The main idea is to use the pairwise inter-cluster distances to define a within-cluster dispersion measure with the original data and a background distribution which reflects 'randomness' and then use statistical measures to compare the within-cluster dispersion measures of the original data distribution with the dispersion in the random case. To ensure the random characteristics of the background distribution, the latter is estimated by averaging several instances of the randomly generated data (*reference* data). For more information and details about the algorithm that estimates the number of clusters in a data matrix using the Gap statistic the user should see (18).

When the user does not have a prior knowledge in how many clusters can the dataset be grouped at, one choice is to use the Gap statistic implementation of ARMADA and based on the estimate returned, perform clustering using the same algorithm and parameters as those used by the Gap statistic tool. To use the Gap statistic tool, the user should select an Analysis from the Analysis Object list and click **Tools** → **Gap Statistic** and the following window will appear:

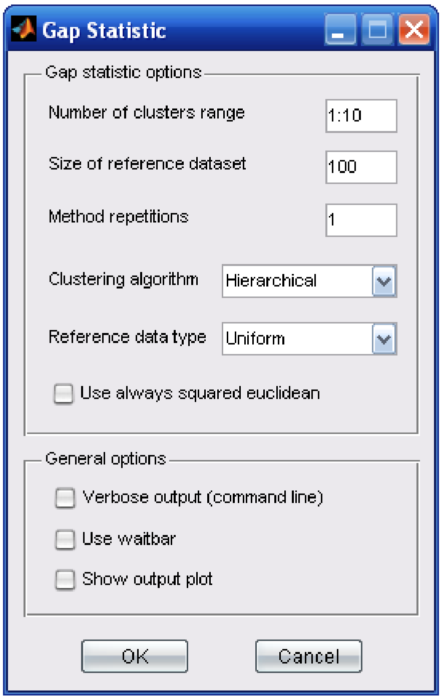

By using this window, the user can set several parameters that will be used for the estimation of the optimal number of clusters. The following table describes each of the user options in the above preferences window:

Gap statistic options

<table>
<tr><th>Option</th><th colspan="2">Description</th></tr>
<tr><td>Number of clusters range</td><td colspan="2">The range of number of clusters form which the optimal number of clusters should be estimated.</td></tr>
<tr><td>Size of reference dataset</td><td colspan="2">How many reference datasets should be randomly created and averaged for the estimation of the background distribution.</td></tr>
<tr><td>Method repetitions</td><td colspan="2">Due to the stochastic nature of the algorithm (randomness of background distribution) the optimal number of times may differ each time. By allowing several repetitions of the whole estimation, the program returns the number of clusters that was found to be optimal in the majority of the repetitions (the most frequent).</td></tr>
<tr><td>Clustering algorithm</td><td colspan="2">The clustering algorithm to be used to cluster data and estimate their within-cluster dispersion measures. It can be one of the clustering algorithms supported by ARMADA (Hierarchical, k-means, Fuzzy C-Means). After selecting the desired algorithm, the respective clustering preferences window will open (the user should see 4.3) allowing parameter setting.</td></tr>
<tr><td>Reference data type</td><td colspan="2">Reference dataset generation method. This option determines the method and the data source from which each reference dataset will be created. The user can select one of the following:</td></tr>
<tr><td></td><td>Uniform</td><td>The reference dataset is created based on uniformly distributed data with ranges taken from the columns (samples, arrays) of the original data matrix.</td></tr>
<tr><td></td><td>Uniform - PCA based</td><td>The reference dataset is created based on uniformly distributed data which were derived by taking into account the shape of the original data, using the principal components of the original data matrix.</td></tr>
<tr><td></td><td>Bootstrap[15]</td><td>The reference dataset is created by bootstrapping the original data matrix.</td></tr>
<tr><td></td><td>Bootstrap - PCA based</td><td>The reference dataset is created by bootstrapping the data matrix derived by taking into account the shape of the original data, using the principal components of the original data matrix.</td></tr>
</table>

---

[15] The bootstrap is an iterative resampling procedure which is based on creating new data by drawing with replacement from an initial dataset. For more information on the bootstrap, the user should see (19. Efron, B. and Tibshirani, R. (1993) *An introduction to the bootstrap*. Chapman & Hall/CRC.

General options	Use always squared euclidean	Whether to always use the squared euclidean distance to calculate the within cluster pairwise distances (as the authors of (18) propose or to use the metric used during the clustering process (sometimes seems to work better).
	Verbose output (command line)	Display output messages on the operating system command line (or MATLAB's command window if ARMADA used under MATLAB) showing different stages of progress.
	Use waitbar	Display a bar showing progress of the calculations.
	Show output plot	If checked, will generate a figure with two panels: the upper panel shows the within-cluster dispersion range (for the original dataset) against the range of the number of clusters. The bottom panel displays the Gap curve which is the values of the Gap statistic against the range of the number of clusters. The user should also see (18).

When choosing the clustering algorithm, each corresponding preferences window contains a field for specifying the number of clusters (e.g. the field **Number of centroids (k)** in the k-means clustering preferences window). This choice is ignored as the number of clusters range is given in the **Number of clusters range** field. After setting the desired parameters, the user should click **OK**. The tool will start the estimation process of the optimal number of clusters (might take some time).

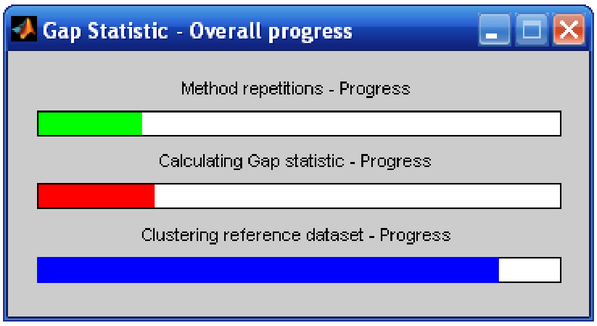

It is recommended to also perform a graphical inspection (by checking the box **Show output plot** in the Gap statistic preferences window) as the algorithm might fall in local minima and not return the true best number of clusters. Ideally, the optimal number of clusters should be the point in the horizontal axis where the within-cluster dispersion range drops steeply and then remains relatively stable across different numbers of clusters. This should also be the point where the Gap curve rises before and drops steeply after. After completing the process, ARMADA will display the following message informing about the result:

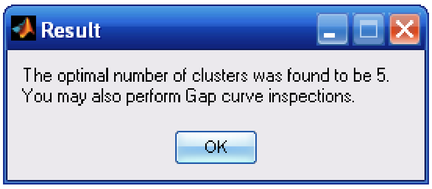

The figure below displays the output plot of the Gap statistic module:

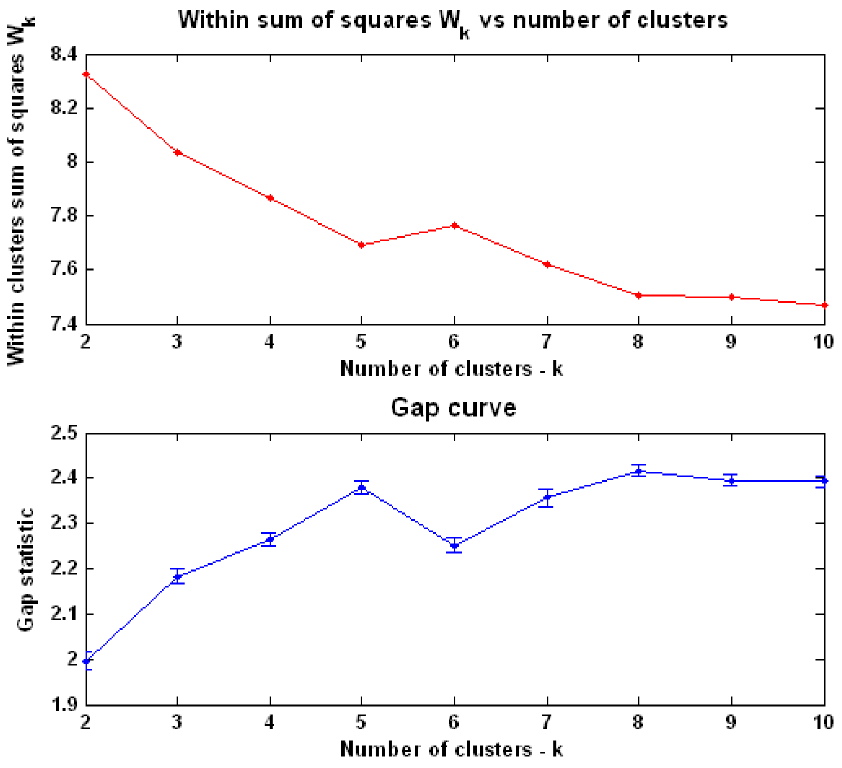

It can be seen in the upper panel that the within-cluster dispersion measure W_k (red line) drops steeply until the number of clusters reaches 5 and then it rises again. Similarly, the Gap curve (blue line) rises until the number of clusters is 5 and then drops. However, it can be observed that W_k drops again until the number of clusters becomes 8 and for a larger number of clusters, presents small changes. Correspondingly, the Gap curve rises until the number of clusters is 8 and then drops very slightly. This fact can give a clue that the algorithm fell on a local minimum and returned 5 as the optimal number of clusters while there might be another optimal solution. This fact does not mean that 5 is not a correct solution but rather that there are more than one possible solutions.

7.3. The Batch Programmer

Many times, depending on the nature of the experiment, it is required to perform several rounds of statistical selection procedures (e.g. when the experiment includes lots of possible contrasts) in order to extract different results corresponding to each case. Moreover, it is possible that the analyst would like to follow different analysis workflows concerning the statistical or the clustering procedures in order to compare different methods or combine the results. As all these cases can take a quite considerable amount of time to be performed, this section presents how multiple analysis steps can be programmed to a batch process through a simple interface provided with ARMADA. In order to use the batch programming module, the user should firstly import data in a new project with one of the ways described in section 2.5. After that, the user is able to program a batch procedure at any time of the analysis. To launch the Batch Programmer, the user should click **Tools → Batch Programmer**. The following window will appear:

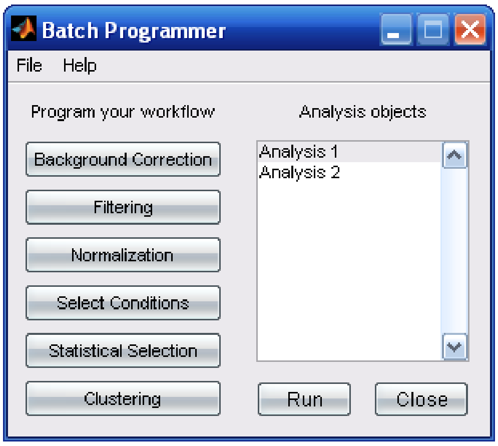

In order to program a batch procedure, the user should first create a new batch file by clicking **File → New batch**. The user will be prompted to select a location for the batch settings file to be created. After this step, the button **Background Correction** will be activated. By hitting the **Background Correction** button, the background correction preferences window will open (section 3.2) and the user should select the preferred background correction method. After setting this, the **Filtering** button will be activated. As with background correction, the filtering preferences window (section 3.3) will open and the user must set the desired parameters. Similarly, the **Normalization** button which will open the normalization preferences window (section 3.4). At this point it should be mentioned that the preprocessing steps are common for the entire dataset.

After properly setting the preprocessing steps, the user can define several Analysis objects through the **Select Conditions** button (section 3.1) and then for each object, define the desired statistical selection workflow and the clustering to be performed (optional). The **Statistical Selection** button will open a preferences window similar to the one of section 4.1 with some differences (figure below):

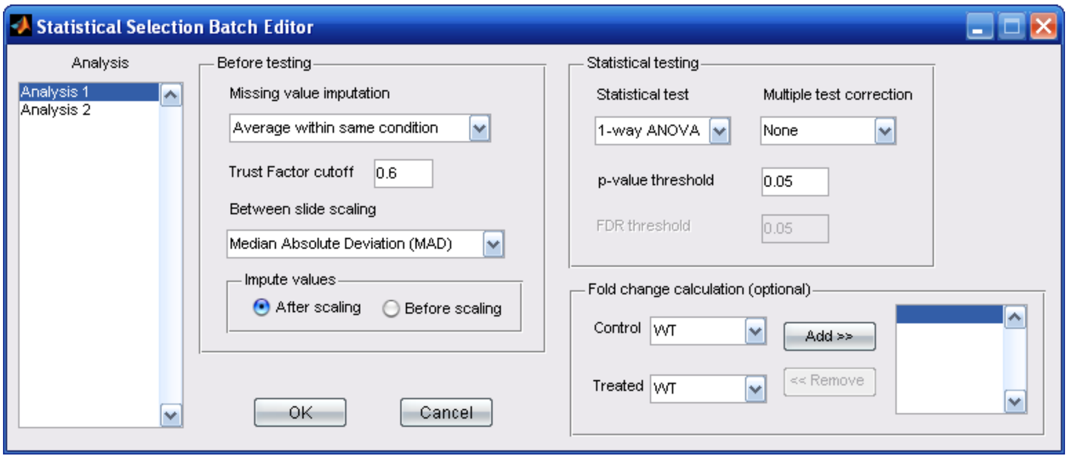

All options are the same as in section 4.1 plus that the user can define pairs of conditions for each Analysis object so that fold changes can be calculated. After making the necessary selections, the user should click **OK**. If **OK** is pressed without making any selections, the default parameters (what is displayed in the window) will be used for the batch process. If the user does not wish to perform statistical tests, **Cancel** should be pressed instead.

The **Clustering** button will open the following window:

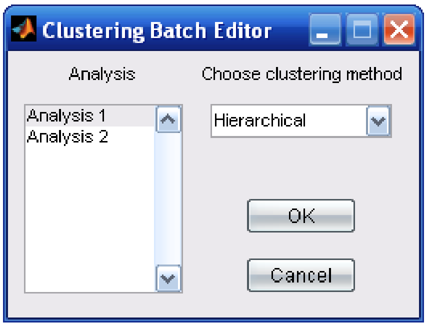

For each Analysis object, the user can define a different clustering algorithm. By selecting an algorithm from the **Choose clustering method** list, the corresponding preferences window will open (the user should see section 4.3) so that parameters can be set (or defaults left). After making the necessary selections, the user should click **OK**. If **OK** is pressed without making any selections, the default parameters (what is displayed in the window, e.g. hierarchical clustering with default parameters) will be used for the batch process. If the user does not wish to perform clustering, **Cancel** should be pressed instead.

At this point, the user can save all previous settings for the defined batch process by clicking **File** → **Save batch** and also save the settings under a different name by clicking **File** → **Save batch as…** It should be mentioned that simply saving a batch will not save the results produced by a batch procedure but it saves only the batch settings. In order to save the results after a batch process is complete, the user should click **File** → **Save batch as…** and in the field **Save as type:** should choose **ADROMEDA Project Files (*.apj)**. In this way, the results are exported as an ARMADA project which can be opened from ARMADA in order to perform data exploration and exporting.

After having set all the obligatory parameters (at least background correction and filtering), the **Run** button will be enabled. In order to start the batch process, the user should click the **Run** button. During the batch process running, the Batch Programmer displays several output messages in the operating system command line or the MATLAB's command window (if ARMADA runs under MATLAB). By clicking **File** → **Exit** or the **Close** button, the Batch Programmer is terminated.

7.4. The Annotator

The ARMADA output files containing gene lists or gene clusters contain only the GeneIDs as gene identifiers. More annotation elements can be easily added to these files using the Annotator module. In order to use the Annotator module, the user should have a complete annotation file for the microarrays used. Such files should be in spreadsheet like format and contain the annotation elements in different columns. One of the columns in the annotation file must be either a slide position (if it is not already contained in the annotation elements, it can be easily created by assigning unique numbers to each spot using e.g. MS Excel) or the same textual identifier as the one imported in ARMADA (usually the chip manufacturer's gene identifier). For more information, the

user should see Appendix A on input file formats. Generally, such annotation files are provided by the chip manufacturer or can be created using public repositories. To launch the Annotator, the user should click **Tools** → **Annotator** and the following window will appear:

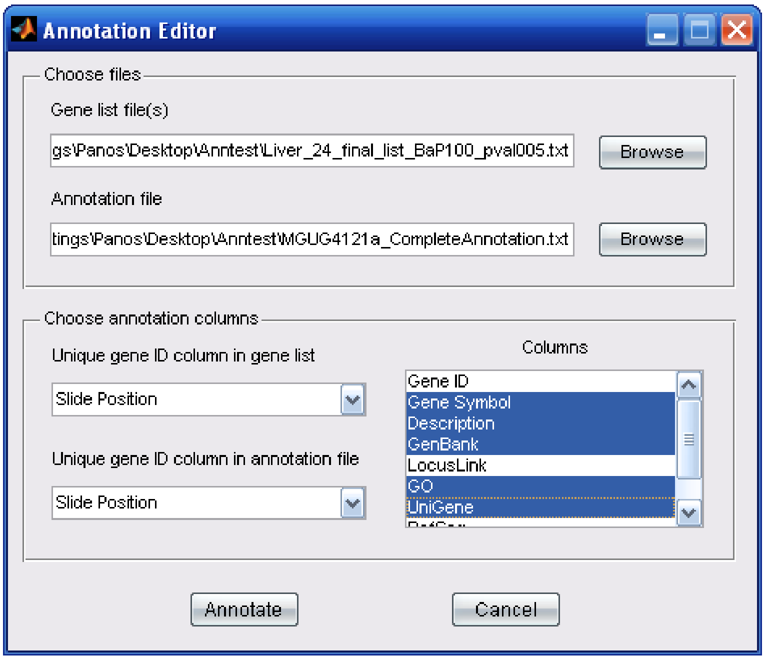

In the **Choose files** panel the user should enter the required files location. In the **Gene list file(s)** field, the user should provide the exact location of the file(s) to be annotated. By clicking the **Browse** button, this task can be completed easily. In the **Annotation file** field, the user should provide the location of the annotation file which can also be done easily with the help of the **Browse** button. Attention should be paid if multiple files are provided in the **Gene list file(s)** field; all of them should be coming from analyses using the same microarray chip (e.g. from the same project) as they all use the specified annotation file, else the program will generate an error. The gene list and annotation files can be either in text tab delimited or Excel format or a mix (e.g. the annotation file is an Excel file and the gene lists are in tab delimited format).

After selecting the necessary files, their column headers are used to fill the **Unique gene ID column in gene list**, the **Unique gene ID column in annotation file** and the **Columns** lists in the **Choose annotation columns** panel. The **Columns** list contains the column headers from the annotation file so the user can choose. The user should choose then the appropriate columns (should contain the same data type, else an error will be generated) and then from the **Columns** list, the user should choose the desired annotation elements to be added to the gene lists. After having made at least one selection from <u>all</u> the lists in the **Choose annotation columns** panel (even if having to reselect the default values) the **Annotate** button will be enabled. The user should click on the **Annotate** button and the Annotator will add annotation elements to the provided files. This process might take some time depending on the number of files to be annotated, their type and their size.

References

1. de Jong, S. and van der Meer, F. (2002) *Imaging spectrometry: basic principles and prospective applications*. Kluwer Academic.
2. Yang, Y.H., Dudoit, S., Luu, P., Lin, D.M., Peng, V., Ngai, J. and Speed, T.P. (2002) Normalization for cDNA microarray data: a robust composite method addressing single and multiple slide systematic variation. *Nucleic acids research*, **30**, e15.
3. Cleveland, W.S., Grosse, E. and Shyu, W.M. (1992) In Chambers, J. M. and Hastie, T. J. (eds.), *Statistical Models in S*. Wadsworth & Brooks/Cole Dormand, J.R.
4. Tseng, G.C., Oh, M.K., Rohlin, L., Liao, J.C. and Wong, W.H. (2001) Issues in cDNA microarray analysis: quality filtering, channel normalization, models of variations and assessment of gene effects. *Nucleic acids research*, **29**, 2549-2557.
5. Troyanskaya, O., Cantor, M., Sherlock, G., Brown, P., Hastie, T., Tibshirani, R., Botstein, D. and Altman, R.B. (2001) Missing value estimation methods for DNA microarrays. *Bioinformatics (Oxford, England)*, **17**, 520-525.
6. Bolstad, B.M., Irizarry, R.A., Astrand, M. and Speed, T.P. (2003) A comparison of normalization methods for high density oligonucleotide array data based on variance and bias. *Bioinformatics (Oxford, England)*, **19**, 185-193.
7. Dudoit, S., Yang, Y.H., Callow, M.J. and Speed, T.P. (2002) Statistical methods for identifying differentially expressed genes in replicated cDNA microarray experiments. *Statistica Sinica*, **12**, 111-140.
8. Benjamini, Y. and Hochberg, Y. (1995) Controlling the False Discovery Rate: a Practical and Powerful Approach to Multiple Testing. *J R Statist Soc*, **57**, 289-300.
9. Storey, J.D. and Tibshirani, R. (2003) Statistical significance for genomewide studies. *Proceedings of the National Academy of Sciences of the United States of America*, **100**, 9440-9445.
10. Speed, T.P. (ed.) (2003) *Statistical analysis of gene expression microarray data*. Chapman & Hall/CRC.
11. Jain, A. and Dubes, R. (1988) *Algorithms for clustering data*. Prentice-Hall, Englewood Cliffs.
12. Dembele, D. and Kastner, P. (2003) Fuzzy C-means method for clustering microarray data. *Bioinformatics (Oxford, England)*, **19**, 973-980.
13. Guyon, I. and Elisseef, A. (2003) An introduction to variable and feature selection. *Journal of Machine Learning Research*, **3**, 1157-1182.
14. Vapnik, V.N. (1995) *Statistical Learning Theory*. Wiley.
15. Furey, T.S., Cristianini, N., Duffy, N., Bednarski, D.W., Schummer, M. and Haussler, D. (2000) Support vector machine classification and validation of cancer tissue samples using microarray expression data. *Bioinformatics (Oxford, England)*, **16**, 906-914.
16. Tukey, J.W. (1977) *Exploratory data analysis*. Addison-Wesley, Reading, MA.
17. Raychaudhuri, S., Stuart, J.M. and Altman, R.B. (2000) Principal components analysis to summarize microarray experiments: application to sporulation time series. *Pac Symp Biocomput*, 455-466.
18. Tibshirani, R., Walther, G. and Hastie, T. (2001) Estimating the number of clusters in a data set via the gap statistic. *J R Statist Soc*, **63**, 411-423.
19. Efron, B. and Tibshirani, R. (1993) *An introduction to the bootstrap*. Chapman & Hall/CRC.

Appendix A: Input file formats

This appendix describes some of the input file formats for ARMADA and provides some links for further user information.

A.1.Raw data – Image analysis software output and tab delimited files

A.1.1. QuantArray file format

QuantArray files contain two main sections: the file header section and the file data section. Below there is one example from each section:

File header section:

User Name	Administrator									
Computer	ARRAYSCANNER									
Date	Wed Apr 06 11:52:47 2005									
Experiment	wt_1_1r									
Experiment Path	C:\Program Files\Packard BioChip\Administrator\ExperimentSets\wt_1_1r									
Protocol	C:\nikosdata\Microarray Experiments VART_VH\QuantArray Protocols\wt_1_1r.pro									
Version	3									
Begin Protocol Info										
Units	Microns									
Array Rows	12									
Array Columns	4									
Rows	21									
Columns	21									
Array Row Spacing	4500									
Array Columns Spacing	4500									
Spot Rows Spacing	200									
Spot Columns Spacing	200									
Spot Diameter	150									
Interstitial	0	0 is off, 1 is first one missing, 2 is second one missing								
Spots Per Array	441									
Total Spots	21168									
Data is not crosstalk corrected.										
Data is background subtracted.										
Quantification Method	Histogram									
Quality Confidence Calculation	Minimum									
End Protocol Info										
Begin Tolerance and Weight										
Measurement	Minimum	Maximum	Weight							
End Tolerance and Weight										
Begin Image Info										
Channel	Image	Fluorophor	Barcode	Units	X Units Per P	Y Units Per F	X Offset	Y Offset	Status	
ch1	C:\nikosdata\Microarray Experiments VART_VH\NI			Microns	10	10	0	0	Control Image	
ch2	C:\nikosdata\Microarray Experiments VART_VH\NI			Microns	10	10	0	0		
End Image Info										
Begin Measurements										
Number	Array Row	Array Column	Row	Column	Name	ch1 Ratio	ch1 Percent	ch2 Ratio	ch2 Percent	Ignore Filter
1	1	1	1	1	CNTRL13L01	1	69.76344	0.433416	30.23656	0
2	1	1	1	2	CNTRL13H13	1	62.457754	0.601082	37.542246	0
3	1	1	1	3	CNTRL13H01	1	70.265147	0.423181	29.734853	0

File data section:

Begin Data																			
Number	Array Row	Array Column	Row	Column	Name	X Location	Y Location	ch1 Intensity	ch1 Backgro	ch1 Intensity	ch1 Backgro	ch1 Diameter	ch1 Area	ch1 Footprint	ch1 Circularit	ch1 Spot Uni	ch1 Bkg. Uni	ch1 Signal N	ch1 Cont
1	1	1	1	1	CNTRL13L01	770	500	12921.821	2248.8059	3041.969	284.53973	147.55473	6700	50.099575	0.875874	0.828156	0.983597	45.413065	
2	1	1	1	2	CNTRL13H13	970	500	9662.4629	4399.8657	562.49719	419.45032	205.29062	6700	50.099575	0.873555	0.966888	0.978821	23.036013	
3	1	1	1	3	CNTRL13H01	1170	500	10498.328	3365.3879	781.38586	400.77405	204.98027	6700	50.099575	0.847978	0.95256	0.978836	26.19513	
4	1	1	1	4	CNTRL13D13	1370	500	8002.2983	2796.0298	692.31842	320.39124	209.43523	6700	50.099575	0.852023	0.957108	0.980789	24.976646	
5	1	1	1	5	CNTRL13D01	1570	500	9017.3584	4482.2686	611.18689	379.53854	217.48761	6700	50.099575	0.858544	0.968292	0.978531	23.758742	
6	1	1	1	6	CNTRL12P13	1770	500	9482.3877	5593.9253	474.96661	317.22812	213.94618	6700	50.099575	0.842386	0.972397	0.981506	29.891385	
7	1	1	1	7	CNTRL12P01	1970	500	12125.851	6803.5522	889.65894	373.46616	210.19377	6700	50.099575	0.817628	0.950897	0.980148	32.468405	
8	1	1	1	8	CNTRL12L13	2170	500	11146.836	7209.4629	491.19296	258.88745	219.09163	6700	50.099575	0.807135	0.970184	0.985779	43.056687	
9	1	1	1	9	CNTRL12L01	2370	500	11596.164	7158.3433	495.09836	337.72617	219.672	6700	50.099575	0.839631	0.973618	0.982162	34.335995	
10	1	1	1	10	CNTRL12H13	2570	500	12259.836	7814.5073	681.32837	320.99799	215.72417	6700	50.099575	0.894478	0.966522	0.981445	38.192875	
11	1	1	1	11	CNTRL12H01	2770	500	12355.91	7936.9106	431.58249	328.43378	220.39533	6700	50.099575	0.871332	0.975143	0.98288	37.620705	
12	1	1	1	12	CNTRL12D13	2970	500	12702.746	8223.9854	526.5022	359.03543	217.63391	6700	50.099575	0.89513	0.972717	0.980637	35.380202	
13	1	1	1	13	CNTRL12D01	3170	500	13819.344	8108.6719	754.2569	364.35492	211.25116	6700	50.099575	0.844908	0.957352	0.977417	37.928248	
14	1	1	1	14	CNTRL11P13	3370	500	10274.687	4831.3584	593.13532	424.8197	193.14742	6700	50.099575	0.782242	0.967316	0.976959	24.185993	
15	1	1	1	15	CNTRL11P01	3570	500	9496.1641	5096.7461	737.14728	331.1965	212.3033	6700	50.099575	0.883919	0.966187	0.982941	28.672296	
16	1	1	1	16	CNTRL11L13	3770	500	8902.6563	5183.4775	488.15201	343.66885	214.6888	6700	50.099575	0.859011	0.974533	0.981689	25.904751	
17	1	1	1	17	CNTRL11L01	3970	500	7916.2539	4486.4028	340.56451	276.11966	216.75458	6700	50.099575	0.814753	0.97879	0.985382	28.66965	
18	1	1	1	18	CNTRL11H13	4170	500	7507.5073	3863.8359	423.10931	258.10379	222.26494	6700	50.099575	0.814878	0.974594	0.985474	29.087164	
19	1	1	1	19	CNTRL11H01	4370	500	5296.1792	2441	330.9722	248.04997	206.21884	6700	50.099575	0.852154	0.98111	0.986099	21.351259	
20	1	1	1	20	CNTRL11D13	4570	500	4452.582	1834.6418	375.99268	212.16736	218.50971	6700	50.099575	0.864272	0.980972	0.987	20.986178	
21	1	1	1	21	CNTRL11D01	4770	500	4059.1792	1575.9552	332.78253	198.21785	209.89069	6700	50.099575	0.875343	0.981781	0.98996	20.478374	
22	1	1	2	1	ZA00003D13	760	730	4600.5371	1608.0299	639.64075	202.92952	205.75525	6700	22.156874	0.934221	0.964981	0.9888	22.670615	
23	1	1	2	2	ZA00003D01	960	730	11968.373	2781.8955	1667.2172	311.36792	163.5177	6700	22.156874	0.827513	0.902557	0.983002	38.438042	
24	1	1	2	3	ZA00002P13	1160	730	13039.836	4738.0449	1183.7947	559.46295	170.56825	6700	22.156874	0.895738	0.932327	0.968094	23.307774	
25	1	1	2	4	ZA00002P01	1360	730	11631.687	4266.7314	724.36023	446.43207	200.74371	6700	22.156874	0.9142	0.960052	0.975891	26.054774	
26	1	1	2	5	ZA00002L13	1560	730	11275.955	3380.2837	1380.069	377.3172	185.41164	6700	22.156874	0.861331	0.923889	0.979614	29.884551	
27	1	1	2	6	ZA00002L01	1760	730	11175.91	4078.1641	990.36328	418.87253	165.45288	6700	22.156874	0.787832	0.948288	0.977585	26.680934	
28	1	1	2	7	ZA00002H13	1960	730	12930.104	5692.6865	1148.3503	337.69244	140.7085	6700	22.156874	0.870585	0.933029	0.9814	38.289588	
29	1	1	2	8	ZA00002H01	2160	730	11374.09	6303.1641	802.45978	368.63205	200.10844	6700	22.156874	0.847684	0.956223	0.980865	30.854859	
30	1	1	2	9	ZA00002D13	2360	730	11531.388	6767.8657	642.56561	426.70816	204.04642	6700	22.156874	0.828643	0.960739	0.977463	27.024062	

Most QuantArray files have the following column headers in the file data section:

Number, Array Row, Array Column, Row, Column, Name, X Location, Y Location, ch1 Intensity, ch1 Background, ch1 Intensity Std Dev, ch1 Background Std Dev, ch1 Diameter, ch1 Area, ch1 Footprint, ch1 Circularity, ch1 Spot Uniformity, ch1 Bkg. Uniformity, ch1 Signal Noise Ratio, ch1 Confidence, ch2 Intensity, ch2 Background, ch2 Intensity Std Dev, ch2 Background Std Dev, ch2 Diameter, ch2 Area, ch2 Footprint, ch2 Circularity, ch2 Spot Uniformity, ch2 Bkg. Uniformity, ch2 Signal Noise Ratio, ch2 Confidence, Ignore Filter.

The user should make sure that at least the column names containing main image quantitation information and spot flags should have the names mentioned above (e.g. ch1 Intensity, ch2 Intensity, ch1 Background, ch1 Background Std Dev, IgnoreFilter etc.) For more information on the necessary quantitation inputs, the user should see section 2.5.2.

A.1.2. ImaGene file format

ImaGene files usually come in pairs, one file for each channel. ARMADA recognizes the correspondence to each channel (channel 1 or Cy3 or 'Green' and channel 2 or Cy5 or 'Red') by their filenames. The file corresponding to each channel should contain the string 'Cy3' or 'Cy5' (depending on the channel) somewhere on its filename (<u>not</u> the file extension). The user should also check section 2.5.1. Below there is an example of an ImaGene file section for one channel (files for the other channel are the same but they contain different values for the main image quantitation types):

Begin Header													
	version	6.0.1											
	Date	Thu Jan 26 11:10:20 CET 2006											
	Image File	E:\ARRAY\Yvonne\Agilent\Mouse lung\images\27772-Cy3 control1 exp1.tif											
	Page	0											
	Page Name												
	Inverted	FALSE											
	Begin Field Dimensions												
		Field	Metarows	Metacols	Rows	Cols							
		A	1	1	215	105							
	End Field Dimensions												
	Begin Measurement parameters												
		Segmentation I	auto										
		Signal Low	0										
		Signal High	0										
		Background Lo	0										
		Background Hi	0										
		Background Bu	2										
		Background W	5										
	End Measurement parameters												
	Begin Alerts												
		Control Type	Minimum thres	If tested	Percentage allo	If failed	Maximum thres	If tested	Percentage allo	If failed	CVthreshold	If tested	If failed
		BLANK	0	FALSE	1.00%	FALSE	500	FALSE	0.10%	FALSE	1	FALSE	FALSE
		POSITIVE	1000	FALSE	0.10%	FALSE	100000	FALSE	1.00%	FALSE	1	FALSE	FALSE
	End Alerts												
	Begin Quality Flags												
		Begin Flagging Settings											
			Empty Spots	TRUE	Threshold:	2							
			Poor Spots	TRUE									
			Begin Poor Spots Parameters										
				Background co	FALSE	Threshold:	0.9995						
				Background te:	TRUE								
				Signal contami	FALSE	Threshold:	0.9995						
				Signal contami	FALSE								
				Ignored percen	TRUE	Threshold:	25						
				Open perimete	TRUE	Threshold:	25						
				Shape regularit	TRUE	Threshold:	0.6						
				Area To Perime	FALSE	Threshold:	0.65						
				Offset flag	TRUE	Threshold:	60						
			End Poor Spots Parameters										
			Negative Spot:	TRUE									
		End Flagging Settings											
		Begin Flagged spots											
			# of Empty Spots: 233										
			# of Poor Spots: 20										
			# of Negative Spots: 0										
			# of Manually Flagged Spots: 1075										
		End Flagged spots											
	End Quality Flags												
End Header													
Begin Raw Data													

	Field	Meta Row	Meta Column	Row	Column	Gene ID	Flag	Signal Mean	Background Me	Signal Median	Background Me	Signal Mode	Background Mo	Signal Area	Background Ar	Signal Total	Background To	Signal Stdev	Bac
	A	1	1	1	1	BrightCorner	1	6312.0761	211.8941	6492	194	6425.7416	167.0144	144	255	908939	54033	2677.8837	
	A	1	1	1	2	BrightCorner	1	6150.6499	215.7006	6252	193	6159.6513	191.6896	140	294	861091	63416	2582.7304	
	A	1	1	1	3	(-)3xSLv1	1	287.8791	189.8994	274	184	273.0799	154.6724	91	338	26197	64186	67.2122	
	A	1	1	1	4	A_51_P185156	0	408.2833	189.8307	403.5	182	400.258	151.0588	120	319	48994	60556	89.2805	
	A	1	1	1	5	A_51_P153113	0	321.6902	182.3016	309	172	311.054	144.7799	113	305	36351	55602	87.074	
	A	1	1	1	6	A_51_P113182	0	296.8275	182.7348	284.5	176.5	299.9655	156.7735	116	298	34432	54455	66.071	
	A	1	1	1	7	A_51_P335050	0	310.787	184.5632	295	177	290.9062	151.3007	108	332	33565	61275	79.8019	
	A	1	1	1	8	A_51_P287810	0	1498.5312	192.2307	1493	179	1588.4509	158.2453	128	312	191812	59976	378.2059	
	A	1	1	1	9	A_51_P256510	0	283.6	179.9329	275	177	277.054	172	105	313	29778	56319	57.9077	
	A	1	1	1	10	A_51_P347103	0	342.3145	182.1224	334.5	175	337.4473	152.4862	124	294	42447	53544	82.0096	

Most ImaGene files have the following data column names:

Field, Meta Row, Meta Column, Row, Column, Gene ID, Flag, Signal Mean, Background Mean, Signal Median, Background Median, Signal Mode, Background Mode, Signal Area, Background Area, Signal Total, Background Total, Signal Stdev, Background Stdev, Shape Regularity, Ignored Area, Spot Area, Ignored Median, Area To Perimeter, Open Perimeter, XCoord, YCoord, Diameter, Position offset, Offset X, Offset Y, Expected X, Expected Y, CM-X, CM-Y, CM Offset, CM Offset-X, CM Offset-Y, Min Diam, Max Diam, Control, Failed Control, Background contamination present, Signal contamination present, Ignored % failed, Open perimeter failed, Shape regularity failed, Perim-to-area failed, Offset failed, Empty spot, Negative spot, Selected spot, Saturated spot.

The user should make sure that at least the column names containing main image quantitation information and spot flags should have the names mentioned above (e.g. Signal Mean, Background Mean, Flag, etc.) For more information on the necessary quantitation inputs, the user should see section 2.5.2. For more information on ImaGene headers, the user should also check http://www.biodiscovery.com/index/imagene.

A.1.3. GenePix file format

GenePix contains quantitation data in one file. Below there is an example of GenePix output:

ATF	1																		
24	43																		
Type=GenePix Results 1.2																			
DateTime=2001/01/18 12:41:32																			
Settings=C:\Axon\Params\9KMO5(011100).gps																			
GalFile=C:\Axon\Params\9KAxonM05All.txt																			
Scanner=GenePix 4000A																			
Comment=																			
PixelSize=10																			
ImageName=635 nm□532 nm																			
FileName=E:\Sean de la Roton\A2 m05-104-1.17 tif.tif□E:\Sean de la Roton\A2 m05-104-1.17 tif.tif																			
PMTVolts=770□780																			
NormalizationFactor:RatioOfMedians=1.05442																			
NormalizationFactor:RatioOfMeans=1.02011																			
NormalizationFactor:MedianOfRatios=1.00515																			
NormalizationFactor:MeanOfRatios=0.715029																			
NormalizationFactor:RegressionRatio=1.18375																			
JpegImage=E:\Sean de la Roton\A1 m05-129-1-17.jpg																			
RatioFormulation=W1/W2 (635 nm/532 nm)																			
Barcode=																			
ImageOrigin=2760, 11790																			
JpegOrigin=3190, 12380																			
Creator=GenePix Pro 3.0.0.98																			
Temperature=1.53816																			
LaserPower=1.23966□2.11066																			
LaserOnTime=27234□28019																			
Block	Column	Row	Name	ID	X	Y	Dia.	F635 Median	F635 Mean	F635 SD	B635 Median	B635 Mean	B635 SD	% > B635+1	% > B635+2	F635 % Sat.	F532 Median	F532 Mean	F53
1	1	1	Mus musculu	834712	3410	12580	80	4122	4442	1673	1487	1895	1628	73	42	0	3265	3716	
1	2	1	ESTs	761302	3650	12580	80	8574	8474	1012	1510	1617	896	100	100	0	7103	7091	
1	3	1	ESTs	761213	3870	12570	120	1705	1783	590	1124	1212	499	60	17	0	923	1252	
1	4	1	ESTs, Moder	804482	4120	12580	90	2844	2997	900	1168	1249	631	92	76	0	2951	2948	
1	5	1	myosin Ib	761018	4380	12580	80	1779	1925	701	1202	1279	424	57	32	0	1559	1611	
1	6	1	ESTs, Highly	805218	4620	12620	170	3441	4833	4571	1184	1392	1162	75	49	0	452	1754	
1	7	1	ESTs, Moder	761038	4850	12580	110	2307	2312	656	1095	1275	1221	47	2	0	2041	2066	
1	8	1	ESTs	774996	5150	12550	140	924	983	316	900	1138	2617	0	0	0	232	252	
1	9	1	ESTs, Highly	779143	5340	12580	90	2403	2902	2179	1170	1639	4145	5	1	0	2029	1856	
1	10	1		780127	5600	12580	100	2527	2381	887	1098	1331	1228	55	6	0	2264	1969	
1	11	1	speckle-type	791503	5880	12540	140	1070	1208	541	1039	1367	1177	3	1	0	285	397	
1	12	1	ESTs, Weakl	959839	6120	12540	140	1105	1480	2614	999	1193	1078	7	1	0	253	332	
1	13	1	ESTs	775407	6370	12550	100	1918	2062	1263	864	1190	1645	18	3	0	1647	1610	
1	14	1	ESTs, Weakl	778381	6620	12550	100	6424	6962	3644	939	1043	489	100	100	0	4152	4362	
1	15	1	ESTs	779847	6860	12550	110	15322	15976	8029	1015	1553	2213	93	87	0	8855	8999	
1	16	1		779824	7100	12550	100	7927	8574	4423	998	1717	2396	88	77	0	4483	4779	
1	17	1	syntrophin, a	318957	7340	12550	110	3206	3201	1693	888	1033	748	77	68	0	1893	1982	
1	18	1	ESTs	329367	7580	12530	140	937	1038	403	840	1076	1237	2	0	0	357	458	
1	19	1	Mus musculu	316187	7900	12560	130	5766	9601	11361	1116	1693	2260	90	50	1	577	1559	
1	20	1	ESTs	313529	8060	12540	120	2267	2709	1815	985	1155	767	74	38	0	1107	1263	
1	21	1	ESTs, Weakl	329563	8320	12540	110	2424	2654	1574	907	993	517	87	68	0	1289	1582	

Most GenePix files have the following headers:

Block, Column, Row, Name, ID, X, Y, Dia., F635 Median, F635 Mean, F635 SD, B635 Median, B635 Mean, B635 SD, % > B635+1SD, % > B635+2SD, F635 % Sat., F532 Median, F532 Mean, F532 SD, B532 Median, B532 Mean, B532 SD, % > B532+1SD, % > B532+2SD, F532 % Sat.,

Ratio of Medians, Ratio of Means, Median of Ratios, Mean of Ratios, Ratios SD, Rgn, Ratio, Rgn R^2, F Pixels, B Pixels, Sum of Medians, Sum of Means, Log Ratio, F635 Median - B635, F532 Median - B532, F635 Mean - B635, F532 Mean - B532, Flags.

The user should make sure that at least the column names containing main image quantitation information and spot flags should have the names mentioned above (e.g. F635 Mean, F635 Median, B635 Mean, Flags, etc.). It has been observed that in some GenePix files, the wavelengths used for the two channels are slightly different. In ARMADA, the two channels must be named with the column names above for GenePix. For example, 532 must correspond to Cy3 or 'Green' and 635 must correspond to Cy5 or 'Red'. For more information on the necessary quantitation inputs, the user should see section 2.5.2. For more information on GenePix headers, the user should also check http://www.moleculardevices.com/pages/software/gn_genepix_file_formats.html#gpr and also http://www.moleculardevices.com/pages/software/gn_gpr_format_history.html.

A.1.4. Text tab delimited files

ARMADA can process other types of raw data which are derived from not yet supported image analysis software (e.g. ArrayVision, Imaging Research Inc.) as long as they have a minimum of quantitation types (section 2.5.2) for both channels and any software dependent headers have been removed so that the file contains only a number of columns with the first row of each column containing the name of the quantitation type. The user can also import text tab delimited files from other sources such as public microarray databases (e.g. ArrayExpress, www.ebi.ac.uk/arrayexpress/ or Gene Expression Omnibus, http://www.ncbi.nlm.nih.gov/geo/).

A.2. Processed data

The user can import already processed data in ARMADA. Such data can be already calculated (but not normalized) expression (natural or $\log_2$ ratio) values or ratio-intensity pairs which can be imported to ARMADA for normalization, and they can also be normalized data (ratios or ratio-intensity pairs) which can be imported to conduct statistical tests. Depending on the input data, some plots might be unavailable.

Concerning the file format of processed data, these files should have only one column with gene ids (accession numbers, manufacturer's ids etc.) and all other columns should be numeric. If only ratio values are available, the file should have as many ratio columns as the number of microarrays in the experiment. The column name should correspond to a unique array identifier string so that it can be used as array unique identifier. For example, if the experiment consists of 20 arrays, the file should have 20+1=21 columns. In the case of ratio-intensity pairs, the file should have (apart from the gene ids column) twice the number of columns as the number of microarrays in the experiment. For example, if the experiment consists of 20 arrays, the file should have (apart from the gene ids column) 20+20+1=41 columns. The user should make sure that there are no extra columns in such

files as they will generate an error. In the case of importing ratio-intensity pairs, the user should make sure that all ratio columns have their intensity pair column. Again, all columns should have a unique name. Additionally, any missing data (missing cells) in the file columns, should be empty or contain the string 'NaN'. Any other string (such as 'NULL') will generate an error. The user can easily replace any other strings with a text editor or spreadsheet software such as MS Excel. Below, there is an example of processed data containing only normalized ratio values:

Reporter name	MBA:MEXP:3759/Normalized	MBA:MEXP:3760/Normalized	MBA:MEXP:3761/Normalized	MBA:MEXP:3762/Normalized	MBA:MEXP:3764/Normalized	MBA:MEXP:3763/Normalized
A2bp1	NaN	NaN	NaN	NaN	NaN	NaN
A2m	NaN	NaN	NaN	NaN	NaN	NaN
Aabp3	1.3828324	1.02274	1.313356	0.14139101	0.21289681	0.15157567
Aadac	NaN	NaN	NaN	NaN	NaN	NaN
Aanat	1.068118	1.0280066	0.90227824	1.1375744	1.0218862	0.8776638
Aatk	0.7039054	0.5187531	0.54615927	0.60616165	0.6087247	0.76790816
Abca1	1.1137171	0.89766896	1.0167725	1.1950728	1.121247	0.96731037
Abca2	1.0917857	0.9480024	0.93968105	0.9698576	0.9271849	0.9192679
Abca4	0.8601027	0.74625766	0.8129847	0.8905621	0.910623	0.6339242
Abca7	1.1535889	1.1185063	1.0396652	0.9910633	1.0259838	0.8701742
Abca8	NaN	NaN	NaN	NaN	NaN	NaN
Abcb10	0.9933904	0.6052491	0.78872824	0.87024313	0.9813563	1.0724393
Abcb11	0.9040716	1.3788409	0.9544007	0.98535085	0.9789672	0.8867802
Abcb1a	0.6597014	0.51996917	0.70184016	0.74078214	0.5194845	1.1298094
Abcb1b	0.99493974	1.031255	1.0593528	0.93215114	0.9361901	0.6081227
Abcb2	1.0212958	0.8207918	0.9988448	0.9787848	0.9318093	0.8729674
Abcb3	0.25131285	0.28731525	0.23878798	0.7716271	0.6258987	1.0946274
Abcb4	NaN	NaN	NaN	NaN	NaN	NaN
Abcb6	0.9235208	1.1582047	0.933917	1.0521237	1.0382375	0.88397783
Abcb7	0.9666409	0.78462976	0.8835252	0.72148424	0.70432127	0.6726146
Abcb9	0.23963909	0.30902624	0.26789376	0.56929696	0.47183698	0.58035475
Abcc1a	NaN	NaN	NaN	NaN	NaN	NaN
Abcc1b	0.3641862	0.51209795	0.45298058	0.31001368	0.29066816	0.24840271
Abcc2	NaN	NaN	NaN	NaN	NaN	NaN
Abcc3	0.5527132	0.70345503	0.5788742	1.7148654	1.1241817	1.8294444
Abcc5a	1.349616	0.98534876	1.0953488	1.3140831	1.7990783	1.7097591
Abcc6	NaN	NaN	NaN	NaN	NaN	NaN
Abcc9	NaN	NaN	NaN	NaN	NaN	NaN
Abcd1	1.0631837	0.7791386	1.0002424	1.5899274	1.1865817	1.6702635
Abcd2	NaN	NaN	NaN	NaN	NaN	NaN
Abcd3	1.0074773	1.0837747	1.0866295	1.3982096	0.907019	0.80726904
Abcd4	0.9015269	0.8946808	0.8703065	0.78884095	0.740609	0.6657123
Abce1	0.9475926	0.62172276	0.74286866	0.45811826	0.7299897	0.44573998
Abcf1	0.50011426	0.3760132	0.48036027	0.47371346	0.45426187	0.74983424
Abcf2	0.60555005	0.45683172	0.61108875	0.40908033	0.48863953	0.9063739
Abcg1	0.2799792	0.34214392	0.3352024	3.8893406	3.5225077	4.3020043
Abcg2	2.0225651	1.2336484	1.7648885	1.1054775	1.2486987	0.6887799
Abcg3	NaN	NaN	NaN	NaN	NaN	NaN
Abcg5	NaN	NaN	NaN	NaN	NaN	NaN
Abcg8	0.9828715	0.7399547	1.052918	0.8763301	0.7645741	0.91664845
Abl1	0.9229394	1.0389895	0.87565833	1.2906467	1.0031981	1.0011867
Ablim1	NaN	NaN	NaN	NaN	NaN	NaN
Abp1	NaN	NaN	NaN	NaN	NaN	NaN
Abt1	0.7731711	0.6483241	0.65794396	0.71175885	0.6947127	0.78653383
Abtb1	0.784732	0.81680393	0.6719965	1.2096801	0.8036048	2.0502825
Acadl	1.229991	0.4709537	0.9617183	0.8761441	2.213873	1.8743774
Acadm	1.1487359	0.719995	0.68005455	1.2773688	1.271536	1.1581745

For more information on importing processed data to ARMADA, the user should also consult section 2.5.3.

A.3. Files used for classification

There are three types of files that can be used as input to classification methods supported in ARMADA: i) files containing new samples to be classified, ii) files containing class prior probabilities to be used with DA classifiers and iii) kernel function parameter files to be used with SVM classifiers. In all cases, the files can be either text tab delimited or Excel files. The following sub-sections give examples of these files.

A.3.1. New sample files

The 1st column of these files should contain as many rows as the number of features (genes) used to train the classifier and each row of the 1st column should contain names for each feature. The 1st row should contain sample names. An instance follows:

GeneID	New_1	New_2	New_3	New_4	New_5	New_6
AFFX-Murf	179.1	195	681	183	67	135
AFFX-hum	4796.7	13842	13201	15404	13159	14220
AFFX-PheX	97.8	-15	-22	-44	115	74
AFFX-HUM	24768.1	5184	33506	34303	28086	29899
AFFX-HUM	364.6	171	1495	2692	259	382
31317_r_at	1825.2	2942	3638	5092	1826	3200
31324_at	478.8	275	419	850	149	372
31326_at	-433.5	-524	-934	-1638	-439	-1003
31331_at	75.9	92	-178	-122	-22	-34
31375_at	229.3	196	765	852	-239	59
31386_at	611.1	-215	871	959	-21	-109
31397_at	162.9	116	383	108	-16	-2
31399_at	238.1	880	1118	1269	496	707
31417_at	330.9	816	1011	749	417	487
31429_at	1694.8	1736	1906	2979	1018	1112
31491_s_a	14.2	101	318	523	132	121
31514_at	512.6	232	1254	2358	883	710
31515_at	-2862.5	-4026	-3710	-5900	-2295	-4154
31534_at	17.2	-767	-1179	-1111	-227	-525

A.3.2. External class prior files for DA classification

The 1st column of these files should contain as many rows as the number of classes in the training dataset and each row should contain one class name. The second column should contain as many rows as the number of classes and each row should have a number between 0 and 1 corresponding to the prior class probability. The sum of the probabilities should be 1. An instance follows:

One	0.1	
Two	0.2	
Three	0.7	

A.3.3. External kernel parameters files for SVM tuning

These files should contain as many columns as the number of parameters that each kernel type accepts. For example, a file with polynomial kernel parameters should contain 3 columns with arithmetic data, while a file with RBF kernel parameters should contain 1 column. There are no headers. An instance follows:

1	0	3
1	0	4
1	0	5
2	0	3
2	0	4
2	0	5
3	0	3
3	0	4
3	0	5

The following table presents the proper order of the columns in the kernel parameters files so as ARMADA interprets them correctly:

**Kernel/Column**	**Column 1**	**Column 2**	**Column 3**
**Polynomial**	Gamma	Coefficient	Degree
**Sigmoid (MLP)**	Gamma	Coefficient	
**RBF**	Gamma		

Appendix B: MATLAB's figure controls

This section is addressed to users not familiar with MATLAB and explains briefly several features that are available for figure control and exploration. It contains several parts of MATLAB's help concerning figures and the user should also check for further information the website http://www.mathworks.com/access/helpdesk/help/techdoc/matlab.html under the section 'Graphics' which explains several features more thoroughly.

B.1. Figures

MATLAB offers a very strong interface for plotting, handling, exploring and exporting figures. The following pictures are taken from MATLAB's help and present a typical figure explaining briefly several toolbars and controls:

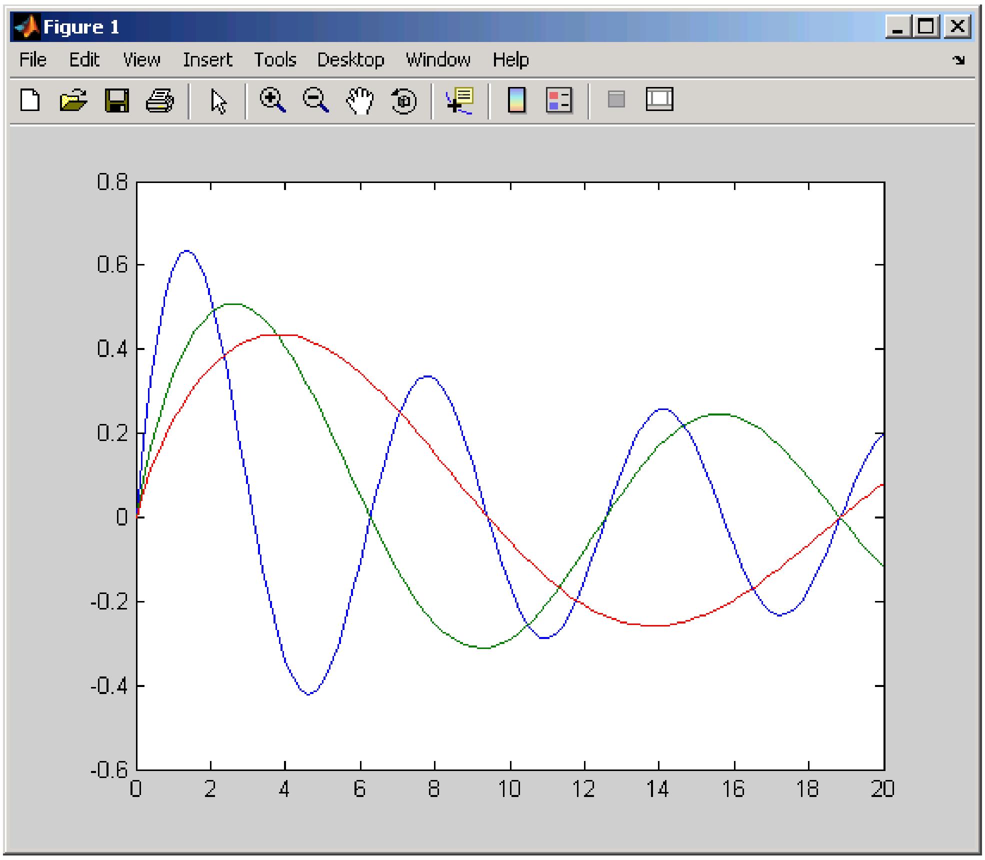

Some of the components and tools of figure windows are called out below:

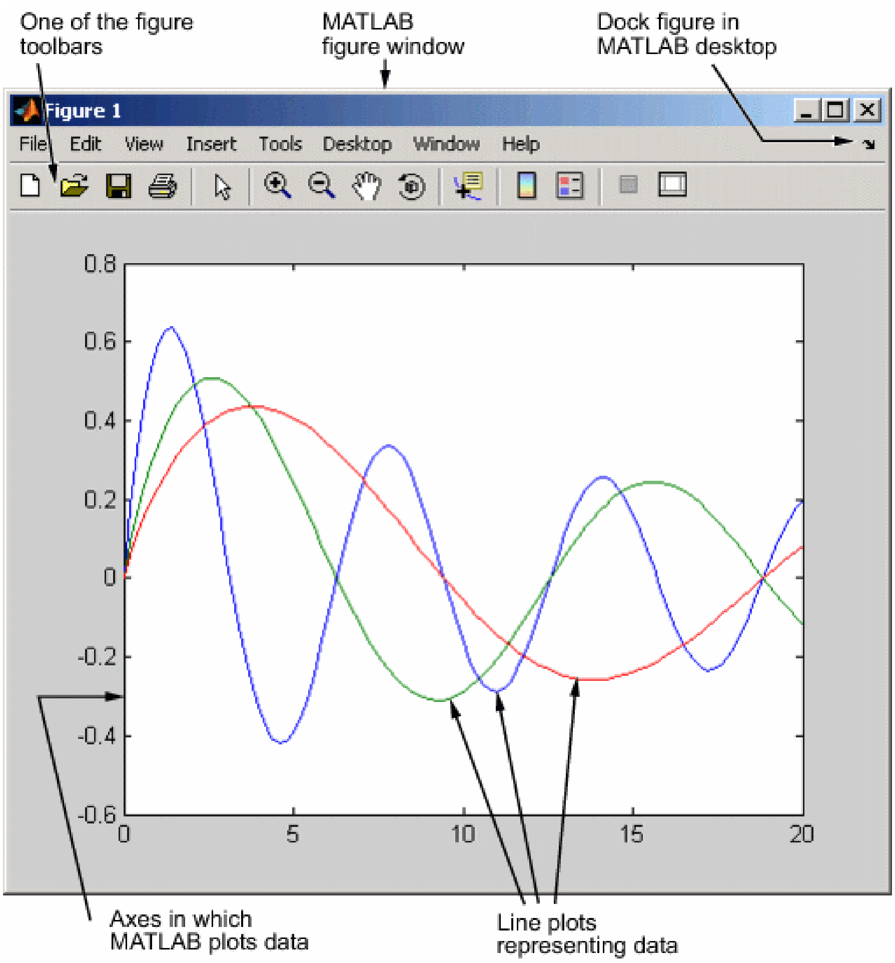

It should be noted that figure docking in MATLAB desktop is available only when MATLAB is present, else, figures will be docked to a common figures window and will be accessible from there as different windows.

B.2. Figure toolbars

Figure toolbars provide shortcuts to access commonly used features. These include operations such as saving and printing, plus tools for interactive zooming, panning, rotating, querying, and editing plots. The following picture shows the features available from this toolbar:

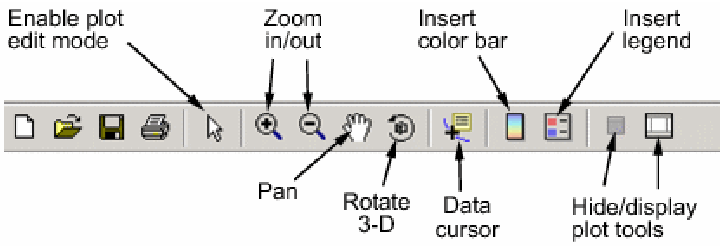

Note that two other toolbars can be enabled from the **View** menu: **Camera Toolbar** which is used for manipulating 3-D views:

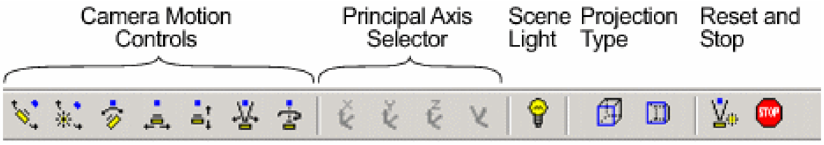

and **Plot Edit Toolbar** which is used for annotation and setting object properties:

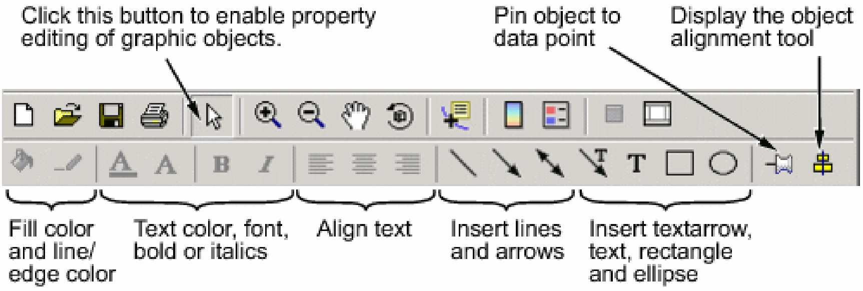

Generally, MATLAB's figure interface offers a lot of possibilities for figure manipulation. Through the figure's several menus, the user can add annotation components (textboxes, arrows etc.) to figures, as well change titles and axes titles, change colors, colormaps, draw elements etc. The user is also able to export figures in many available formats. For more thorough information and examples on MATLAB's figure interfaces and possibilities, the user should check http://www.mathworks.com/access/helpdesk/help/techdoc/matlab.html under 'Graphics'.

Appendix C: Multiple testing correction issues

Hypothesis testing in statistics involves two kinds of errors: the Type I error occurs when the null hypothesis is incorrectly rejected. In the context of microarray experiments, type I errors are committed when a gene is declared differentially expressed while it is not. On the other hand, Type II errors occur when the null hypothesis is not rejected while it is false, that is, in the context of microarrays, when the test fails to identify a differentially expressed gene. When conducting multiple testing, the probability of Type I errors is increased proportionally to the number of tests. This is allowable when the number of tests is small, but in the case of microarrays where thousands of tests are performed, a large number of false positives is undesirable. For example, if an experiment involves testing over 10000 genes and the p-value threshold to determine differential expression is set to 0.01, then at least 100 false positives are expected. Such unwelcome results necessitate the correction of statistical scores to adjust for multiple hypothesis testing.

There exist two main categories of multiple testing correction methods: the Family Wise Error Rate (FWER) and the False Discovery Rate (FDR) methods. FWER procedures correct for multiple testing by adjusting p-values to account for multiple testing. For example the Bonferroni procedure adjusts p-values by dividing with the number of hypotheses n to be tested ($p_{adj}=p/n$). FWER methods are unsuitable for microarray data mostly because they are too conservative: after correcting for multiple testing, no single gene may meet the threshold for statistical significance. In contrast, FDR methods, instead of adjusting p-values, they seek to minimize the proportion of errors committed by falsely rejecting null hypotheses. As they are less stringent than FWER methods they are considered more suitable for microarray data. However, a common drawback with both of them is that they do not assume general variable dependence which is usually the case for microarrays because genes are involved in complicated interaction networks and pathways.

Appendix D: Distance metrics and linkage algorithms

This appendix describes the linkage algorithms used in hierarchical clustering (section 4.3.1) and the distance metrics used in several processes in ARMADA. The descriptions below are based on MATLAB's help.

D.1. Distance metrics

Let X denote a $m \times n$ data matrix whose m rows can be thought as m vectors each consisting of n elements (dimensions). The following table defines the various distances between two row vectors x_i and x_j of the matrix X:

**Distance**	**Definition**
Euclidean	$d_{ij} = \sqrt{(x_i - x_j)(x_i - x_j)^T}$.
Standardized Euclidean	$d_{ij} = \sqrt{(x_i - x_j) D^{-1} (x_i - x_j)^T}$, where D is the diagonal matrix with diagonal elements given by υ_r^2 which denotes the variance of the variable x_j over the m objects.
Mahalanobis	$d_{ij} = \sqrt{(x_i - x_j) V^{-1} (x_i - x_j)^T}$, where V is the sample covariance matrix.
City Block (Manhattan)	$d_{ij} = \sum_{r=1}^{n} \lvert x_{ir} - x_{jr} \rvert$.
Minkowski	$d_{ij} = \left\{ \sum_{r=1}^{n} \lvert x_{ir} - x_{jr} \rvert^{p} \right\}^{\frac{1}{p}}$. It can be easily seen that for the special case of p=1, the Minkowski metric gives the City Block metric, and for the special case of p=2, the Minkowski metric gives the Euclidean distance.
Cosine	$d_{ij} = \left(1 - \frac{x_i x_j^T}{(x_i^T x_i)^{\frac{1}{2}} (x_j^T x_j)^{\frac{1}{2}}} \right)$.
Correlation	$d_{ij} = 1 - \frac{(x_i - \overline{x_i})(x_j - \overline{x_j})^T}{\sqrt{(x_i - \overline{x_i})(x_i - \overline{x_i})^T} \sqrt{(x_j - \overline{x_j})(x_j - \overline{x_j})^T}}$, where $\overline{x_i} = \frac{1}{n} \sum_{r=1}^{n} x_{ir}$ and $\overline{x_j} = \frac{1}{n} \sum_{r=1}^{n} x_{jr}$.
Hamming	$d_{ij} = \left(\frac{\#(x_{ir} \neq x_{jr})}{n} \right)$.
Jaccard	$d_{ij} = \frac{\#\left[(x_{ir} \neq x_{jr}) \wedge \left((x_{ir} \neq 0) \vee (x_{jr} \neq 0) \right) \right]}{\#\left[(x_{ir} \neq 0) \vee (x_{jr} \neq 0) \right]}$.

D.2. Linkage algorithms

These linkage algorithms are based on different ways of measuring the distance between two clusters of objects. If n_i is the number of objects in cluster i and n_j is the number of objects in cluster j, and x_{ir} is the rth object in cluster i, the definitions of these various measurements is presented in the following table:

Algorithm	Definition
Single	Single linkage is also called nearest neighbor and uses the smallest distance between objects in the two clusters. It is defined as: $l(i,j) = min\left(d\left(x_{ir}, x_{js}\right)\right),\ r \in \left(r,...,n_i\right),\ s \in \left(1,...,n_j\right)$.
Complete	Complete linkage is also called furthest neighbor and uses the largest distance between objects in the two clusters. It is defined as: $l(i,j) = max\left(d\left(x_{ir}, x_{js}\right)\right),\ r \in \left(r,...,n_i\right),\ s \in \left(1,...,n_j\right)$.
Average	Average linkage uses the average distance between all pairs of objects in cluster i and cluster j. It is defined as: $l(i,j) = \frac{1}{n_i n_j}\sum_{r=1}^{n_i}\sum_{s=1}^{n_j} d\left(x_{ir}, x_{js}\right)$.
Centroid	Centroid linkage uses the Euclidean distance between the centroids of the two clusters. It is defined as: $l(i,j) = \left\lVert \overline{x_i} - \overline{x_j} \right\rVert_2$, where $\overline{x_i} = \frac{1}{n_i}\sum_{r=1}^{n_i} x_{ir}$, $\overline{x_j} = \frac{1}{n_j}\sum_{r=1}^{n_j} x_{jr}$.
Median	Median linkage uses the Euclidean distance between weighted centroids of the two clusters. It is defined as: $l(i,j) = \left\lVert \widetilde{x_i} - \widetilde{x_j} \right\rVert_2$, where $\widetilde{x_i}$ and $\widetilde{x_j}$ are weighted centroids for the clusters i and j. If cluster i was created by combining clusters p and q, then $\widetilde{x_i}$ is defined recursively as: $\widetilde{x_i} = \frac{1}{2}\left(\widetilde{x_p} + \widetilde{x_q}\right)$ and $\widetilde{x_j}$ is defined similarly.
Ward	Ward's linkage uses the incremental sum of squares; that is, the increase in the total within-cluster sum of squares as a result of joining clusters i and j. The within-cluster sum of squares is defined as the sum of the squares of the distances between all objects in the cluster and the centroid of the cluster. The equivalent distance is given by $l(i,j) = \sqrt{n_i n_j \frac{\left\lVert \overline{x_i} - \overline{x_j} \right\rVert_2}{\left(n_i + n_j\right)}}$ where $\lVert\ \rVert_2$ is the Euclidean distance and $\overline{x_i}, \overline{x_j}$ as in the Centroid linkage respectively.
