# Supplementary figures and images for: Gene ARMADA: an integrated multi-analysis platform for microarray data implemented in MATLAB
Source: BMC Bioinformatics. 2009 Oct 27;10:354. doi: 10.1186/1471-2105-10-354 (PMC2771024; doi:10.1186/1471-2105-10-354)

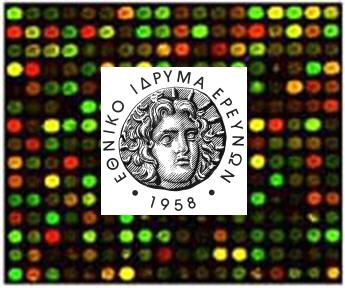

Supplement: Additional file 1 — Source code of Gene ARMADA. Gene ARMADA's MATLAB source code. The file should be extracted using a suitable program (e.g. Winzip, WinRAR, gzip or 7-Zip) and the extracted files and folders should be placed in MATLAB's path. The program can then start by typing ARMADA in MATLAB's command line. [file 1471-2105-10-354-S1.ZIP › ARMADA/logo.jpg]
